# Global, regional, and national prevalence and trends of gynecological diseases among women of childbearing age from 1990 to 2021: An analysis of the global burden of disease study 2021

**DOI:** 10.1371/journal.pone.0329336

**Published:** 2025-08-01

**Authors:** Xiaofeng He, Jiao Su, Kunbo Wang, Yuanhao Liang, Long Wang

**Affiliations:** 1 Institute of Evidence-Based Medicine, Heping Hospital Affiliated to Changzhi Medical College, Changzhi, China; 2 Department of biochemistry, Changzhi Medical College, Changzhi, China; 3 Xiangya School of medicine, Central South University, Changsha, China; 4 Clinical Experimental Center, Jiangmen Central Hospital, Jiangmen, China; 5 Department of Neurosurgery, Heping Hospital Affiliated to Changzhi Medical College, Changzhi, China; Dipartimento di Scienze Mediche e Chirugiche (DIMEC), Orsola Hospital, ITALY

## Abstract

**Purpose:**

The global prevalence and trends of gynecological diseases (GDs) among women of childbearing age (WCBA) remain unclear and may be underestimated. This study aims to evaluate the prevalence of GDs at global, regional, and national levels and assess changes from 1990 to 2021.

**Methods:**

Data on the annual prevalence of major GDs, including uterine fibroids, polycystic ovarian syndrome (PCOS), female infertility, endometriosis, genital prolapse, premenstrual syndrome (PMS), and other GDs, were obtained from the Global Burden of Diseases, Injuries, and Risk Factors Study (GBD) 2021. The study analyzed women aged 15–49 years across 204 countries and territories from 1990 to 2021. Estimated annual percentage changes (EAPC) in the age-standardized prevalence rate (ASPR) were calculated to quantify temporal trends, by age and socio-demographic index (SDI).

**Results:**

In 2021, the global prevalence of GDs among WCBA was estimated at 1.21 billion cases, corresponding to a ASPR of 62,091.73 cases per 100,000 population (95% UI: 62,088.24 to 62,095.23). While the ASPR for GDs remained stable from 1990 to 2021 (EAPC = 0% [95% CI: –0.03 to 0.02]), the number of prevalent cases doubled over the same period. In 2021, the most prevalent GD globally was PMS, followed by uterine fibroids, PCOS, and female infertility. Conditions such as uterine fibroids, PCOS, and female infertility showed a significant upward trend in ASPR over time. Additionally, the ASPR of most GDs generally decreased with rising SDI, except for PCOS, which exhibited an increasing trend with higher SDI. The prevalence of GDs also increased with age, peaking in the 40–44 years age group. However, a shift in the burden of GDs toward younger women was observed, with significant increases in prevalence rates for uterine fibroids, PCOS, female infertility, and PMS in women aged 20–29 years.

**Conclusions:**

GDs among WCBA remain a global concern, underscoring the urgent need for targeted interventions, especially for younger populations and in regions with limited healthcare infrastructure. Prioritizing early intervention, addressing environmental risk factors, and removing barriers to healthcare access will mitigate the long-term impact of these conditions on women’s health and overall well-being.

## Introduction

Gynecological diseases (GDs)—including endometriosis, uterine fibroids, and female infertility—pose a significant but often overlooked threat to women’s health worldwide [1–5]. Women of childbearing age (WCBA), defined as those aged 15–49, are especially vulnerable to these conditions [5]. For instance, endometriosis affects around 10% of WCBA globally, yet access to standardized and effective care remains limited [6]. These diseases not only impact reproductive health but also lead to substantial psychological and socioeconomic burdens [7–9]. Despite their serious health implications, most existing studies focus on individual conditions, specific populations [10–12], or all age groups combined [5], rather than addressing GDs specifically in WCBA.

The changing landscape of risk factors—such as delayed childbearing [13], increasing rates of metabolic syndrome [14,15], and greater exposure to environmental endocrine disruptors [16]—further highlights the need for a systematic assessment of trends over time [17]. In addition, the burden of GDs differs significantly across regions and socio-demographic groups, making it crucial to understand geographical disparities and temporal changes in prevalence [5]. As global attention to women’s health grows [18], accurate and comprehensive data on GD prevalence are urgently needed.

Methodological inconsistencies and the often mild or asymptomatic nature of early-stage GDs contribute to their underestimation in routine health statistics [19,20]. The Global Burden of Diseases, Injuries, and Risk Factors Study (GBD) 2021 provides a valuable opportunity to address these gaps by applying standardized case definitions, using the American College of Obstetricians and Gynecologists (ACOG) criteria where applicable [1]. This study aims to improve the epidemiological understanding of GDs by estimating their prevalence at global, regional, and national levels and analyzing trends over recent decades.

## Methods

### Overview

The estimates presented in this study were obtained from the GBD 2021 [1]. With each new release of the GBD, data are updated, and methodological improvements are implemented; therefore, estimates for the entire time series replace those from previous GBD cycles [21]. This section outlines the key methodological steps used in generating the estimates reported in this study. More detailed descriptions of the methods can be found in the Supporting information and the GBD 2021 methods appendices (https://www.healthdata.org/gbd/methods-appendices-2021/gynaecological-diseases).

The GBD 2021 study was approved by the Institutional Review Board committee at the University of Washington. Informed consent was waived due to the use of deidentified data (https://www.healthdata.org/research-analysis/gbd).

### Definitions

All estimates in this study pertain to women of childbearing age (WCBA), defined as those aged 15–49 years according to World Health Organization (WHO) criteria [22,23]. The estimates are presented globally, by region, and across 5–year age groups (15–19, 20–24, 25–29, 30–34, 35–39, 40–44, and 45–49) for the years 1990–2021. Regional estimates are based on geographic classifications, including 21 GBD world regions (S1 Table in S1 File) and 204 countries or territories. Additionally, countries and territories were categorized into five quintiles based on the Socio-demographic Index (SDI): low, low-middle, middle, high-middle, and high (S2 Table in S1 File). The SDI is a composite measure of social development, calculated as the geometric mean of three standardized indicators: the total fertility rate among women under 25 years, the average years of schooling for individuals aged 15 and older, and lag-distributed income per capita [1]. All rates are reported per 100,000 people per year, with 95% uncertainty intervals (UIs) derived from the 25th and 975th values of 1,000 draws, which were propagated through each estimation step [1].

In the GBD 2021 study, GDs were defined using standardized diagnostic criteria, with the ACOG guidelines serving as the reference standard where applicable. The analysis focused on the prevalence of key GDs, including uterine fibroids, polycystic ovarian syndrome (PCOS), female infertility, endometriosis, genital prolapse, premenstrual syndrome (PMS), and a category of other GDs. The “other GDs” category encompasses conditions such as inflammatory disease of the cervix uteri, diseases of Bartholin’s gland, other inflammatory disorders of the vagina and vulva, vulvovaginal ulceration and inflammation, and non-inflammatory disorders of the ovary, fallopian tube, and broad ligament [1]. All diseases were classified according to the corresponding codes of International Classification of Diseases, 10th Revision (ICD-10), as adopted in the GBD study. A detailed mapping of these classifications is provided in S3 Table in S1 File.

The GBD methodology integrates data from diverse sources—including vital registration, surveys, hospital records, and disease registries—using advanced statistical models such as DisMod-MR 2.1, a Bayesian meta-regression tool [1]. This tool enables the synthesis of heterogeneous data while adjusting for known biases and inconsistencies (e.g., differences in case definitions or reporting practices across countries and time periods). As part of the GBD estimation process, internal validation techniques are applied, including out-of-sample predictive validity checks and data quality scoring, to assess the robustness of model estimates.

### Data source and data collection

The GBD, led by the Institute for Health Metrics and Evaluation (IHME), is a collaborative research initiative that estimates global trends in population, fertility, morbidity, and mortality [21]. GBD 2021 integrates a wide range of data sources, including surveys, censuses, vital statistics, and other health-related records, covering 204 countries and territories from 1990 to 2021. In GBD 2021, prevalence estimates for GDs were generated using the Bayesian meta-regression tool DisMod-MR 2.1. This modeling framework synthesizes data from population surveys, cohort studies, health system administrative records, and registry microdata, ensuring internal consistency across different regions, age groups, and time periods [1]. To minimize the impact of data heterogeneity, standardization and calibration steps were applied during the estimation process.

This study utilized annual estimates of region-, country-, and age-specific prevalence numbers and rates for GDs among women aged 15–49 years from 1990 to 2021. These estimates were obtained from GBD 2021 through the Global Health Data Exchange (GHDx) query tool (http://ghdx.healthdata.org/gbd-results-tool).

### Statistical analysis

This study calculated the age-standardized prevalence rate (ASPR) of GDs per 100,000 population employing the following formula:


$$\text{ASPR}=\frac{\sum_{i=1}^{A}a_{i}w_{i}}{\sum_{i=1}^{A}w_{i}}\;\times 100,000$$

In this formula, $a_{i}$ represents the age-specific rate for the $i^{th}$ age subgroup, while $w_{i}$ denotes the corresponding population count for that age subgroup *i*, sourced from the GBD Study Population Estimates (1950–2021) [24]. *A* represents the total number of age groups. The global age-standardized population used for these calculations was obtained from the World Standards database, developed by the WHO (https://seer.cancer.gov/stdpopulations/world.who.html). ASPR is the prevalence rate of a condition standardized to a global age structure to allow for comparisons across populations with different age distributions [25].

To assess temporal trends in the prevalence of infertility, we calculated the estimated annual percentage change (EAPC) in the ASPR [26]. EAPC is a summary measure used to quantify the average annual change in age-standardized rates over a specified period, derived from a log-linear regression model [27]. This was done by fitting a regression model to the natural logarithm of the ASPR, expressed as y=α+βx+ε, where y=ln(ASPR) and x represents the calendar year. The EAPC was then computed using the formula 100×(exp(β)−1), with the corresponding 95% confidence interval (CI) derived from the linear regression model. An ASPR was considered to show an increasing or decreasing trend over time if the EAPC and its corresponding 95% CI were entirely above or below zero, respectively. If the 95% CI included zero, the change in ASPR was regarded as statistically insignificant.

Additionally, a Locally Weighted Scatterplot Smoothing (LOWESS) model was used to examine the correlation between the ASPR and the SDI across 21 regions [28]. LOWESS is a non-parametric regression technique that fits a smooth curve through the data points, allowing for visualization of potential nonlinear trends without assuming a specific model structure [29]. Spearman correlation analyses were conducted to calculate the correlation coefficient (*r*) and corresponding *p*-values, assessing the strength and significance of the relationship between ASPR and SDI.

All statistical analyses and mapping were performed using R software, version 4.2.3 (R Foundation for Statistical Computing), with significance set at *P* < 0.05.

### Ethics approval and consent to participate

The GBD 2021 study was approved by the Institutional Review Board committee at the University of Washington. Informed consent was waived due to the use of deidentified data (https://www.healthdata.org/research-analysis/gbd).

## Results

### The prevalence of overall GDs

**Fig 1:**
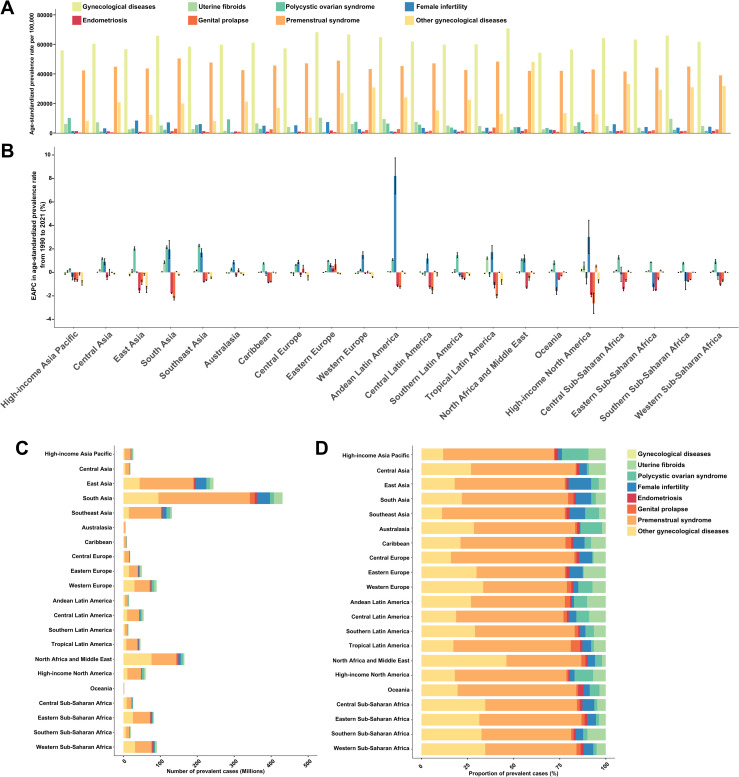
Prevalence of gynecological diseases (GDs) among women of childbearing age in 2021, along with their changes from 1990 to 2021, and the contribution of each GD to the overall prevalence, by 21 GBD regions. **(A)** Age-standardized prevalence rate of GDs in 2021. **(B)** Estimated annual percentage changes in the age-standardized prevalence rate of GDs from 1990 to 2021. **(C)** Number of prevalent cases of GDs in 2021. **(D)** Proportions of prevalent cases attributed to each GD in 2021. GBD = Global Burden of Diseases, Injuries, and Risk Factors Study.

**Fig 2:**
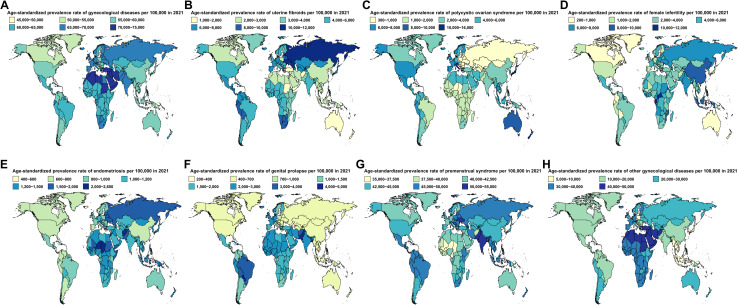
Age-standardized prevalence rates of gynecological diseases across 204 countries and territories in 2021. **(A)** Overall gynecological diseases. **(B)** Uterine fibroids. **(C)** Polycystic ovarian syndrome. **(D)** Female infertility. **(E)** Endometriosis. **(F)** Genital prolapses. **(G)** Premenstrual syndrome. **(H)** Other gynecological diseases.

Globally, an estimated 1.21 billion prevalent cases of GDs among WCBA were reported in 2021, corresponding to an ASPR of 62,091.73 cases per 100,000 population (95% UI: 62,088.24 to 62,095.23) (Table 1). Among the 21 GBD regions, North Africa and the Middle East had the highest ASPR (70,779.89; 95% UI: 70,766.79 to 70,792.99), while South Asia recorded the highest number of prevalent cases, reaching 321.52 million (Fig 1A and 1C). At the national level, Iran had the highest ASPR (72,483.08; 95% UI: 72,448.66 to 72,517.51), followed by Turkey (71,747.88; 95% UI: 71,712.42 to 71,783.35) (Fig 2A).

**Table 1:** The global prevalence of gynecological diseases among women aged 15-49 years in 1990 and 2021, along with the trends and changes observed between these years.

Characteristics	1990		2021		1990 − 2021	
	Number of cases (Millions)	Age-standardized rate per 100,000 population (95% UI)	Number of cases (Millions)	Age-standardized rate per 100,000 population (95% UI)	Percentage change in absolute number (%)	Estimated annual percentage changes (95% CI)
Global	797.24	61465.62 (61461.29 to 61469.96)	1211.53	62091.73 (62088.24 to 62095.23)	51.96	0 (−0.03 to 0.02)
Socio-demographic index						
High	135.99	59434.76 (59424.75 to 59444.77)	150.68	60445.48 (60435.72 to 60455.25)	10.8	0.03 (0 to 0.07)
High-middle	170.37	62292.96 (62283.52 to 62302.41)	192.01	60967.85 (60959.05 to 60976.65)	12.7	−0.14 (−0.18 to −0.11)
Middle	260.87	60846.11 (60838.5 to 60853.71)	385.01	61651.2 (61645.03 to 61657.37)	47.59	0 (−0.03 to 0.02)
Low-middle	164.05	63258.39 (63248.44 to 63268.34)	319.35	64212.15 (64205.07 to 64219.24)	94.66	0.03 (0.02 to 0.05)
Low	65.24	62657.05 (62641.29 to 62672.81)	163.57	63468.3 (63458.27 to 63478.34)	150.7	0.04 (0.03 to 0.05)
Cause						
Uterine fibroids	48.08	4137.13 (4135.95 to 4138.31)	85.18	4352.34 (4351.41 to 4353.26)	77.16	0.15 (0.12 to 0.18)
Polycystic ovarian syndrome	34.81	2637.38 (2636.49 to 2638.27)	65.77	3372.56 (3371.75 to 3373.38)	88.95	0.73 (0.7 to 0.77)
Female infertility	59.69	4630.6 (4629.42 to 4631.78)	110.09	5636.81 (5635.76 to 5637.86)	84.44	0.69 (0.52 to 0.86)
Endometriosis	19.08	1466.85 (1466.18 to 1467.52)	21.05	1077.83 (1077.37 to 1078.29)	10.28	−1.03 (−1.08 to −0.98)
Genital prolapse	22.99	2076.14 (2075.29 to 2077)	32.02	1634.39 (1633.82 to 1634.96)	39.29	−0.96 (−1.04 to −0.88)
Premenstrual syndrome	593.8	45110.81 (45107.12 to 45114.49)	889.97	45637.68 (45634.68 to 45640.68)	49.88	0.01 (−0.01 to 0.03)
Other gynecological diseases	258.71	21150.47 (21147.87 to 21153.08)	400.68	20492.62 (20490.61 to 20494.62)	54.88	−0.22 (−0.28 to −0.15)
Age group (years)						
15–19	98.81	38668.32 (27868.77 to 53436.55)	117.91	38830.68 (28166.47 to 53561.76)	19.33	0 (−0.03 to 0.02)
20–24	124.9	51161.1 (39527.5 to 62534.61)	160.17	54525.06 (42391.28 to 66676.86)	28.24	0.16 (0.13 to 0.19)
25–29	130.82	59437.28 (49130.68 to 69608.01)	179.03	61523.52 (50768.29 to 71685.06)	36.85	0.07 (0.05 to 0.09)
30–34	127.78	67215.51 (56104.53 to 78224.69)	202.22	67648.29 (56222.15 to 79338.92)	58.25	0 (−0.02 to 0.02)
35–39	131.28	75687.25 (65970.5 to 84752.56)	211.38	76088.31 (66533.03 to 85395.85)	61.01	−0.01 (−0.03 to 0.02)
40–44	108.51	77383.5 (67474.07 to 85863.19)	191.53	77199.86 (67362.31 to 85711.91)	76.5	−0.05 (−0.08 to −0.02)
45–49	75.13	66020.61 (57607.51 to 75437.31)	149.3	63359.63 (54931.02 to 73353.73)	98.72	−0.2 (−0.23 to −0.16)
GBD regions						
High-income Asia Pacific	26.03	56723.22 (56701.35 to 56745.1)	21.69	55851.42 (55827.13 to 55875.73)	−16.66	−0.11 (−0.19 to −0.04)
Central Asia	9.62	60105.26 (60065.59 to 60144.95)	14.79	60358.38 (60327.53 to 60389.25)	53.71	0.01 (0 to 0.02)
East Asia	189.17	58540.29 (58531.74 to 58548.84)	192.68	56712.17 (56703.94 to 56720.4)	1.85	−0.23 (−0.29 to −0.17)
South Asia	158.06	64627.48 (64617.17 to 64637.78)	321.52	65798.91 (65791.68 to 65806.13)	103.42	0.05 (0.04 to 0.07)
Southeast Asia	67.15	57618.39 (57604.19 to 57632.6)	107.35	58424.14 (58413.08 to 58435.19)	59.86	0.04 (0.04 to 0.05)
Australasia	3.27	60673.58 (60607.69 to 60739.51)	4.39	59739.13 (59682.83 to 59795.47)	34.38	−0.05 (−0.05 to −0.04)
Caribbean	5.45	60852.48 (60800.33 to 60904.67)	7.33	61038.18 (60993.99 to 61082.39)	34.55	0 (0 to 0.01)
Central Europe	17.85	57614.53 (57587.73 to 57641.35)	15.2	57206.52 (57176.9 to 57236.16)	−14.84	−0.02 (−0.05 to 0)
Eastern Europe	38.24	68143.62 (68121.87 to 68165.36)	34.53	68189.3 (68165.66 to 68212.95)	−9.7	−0.01 (−0.02 to −0.01)
Western Europe	65.79	68339.59 (68323.05 to 68356.14)	63.65	66643.17 (66626.55 to 66659.79)	−3.26	−0.09 (−0.09 to −0.08)
Andean Latin America	5.71	63543.7 (63489.89 to 63597.55)	11.27	64813.03 (64775.12 to 64850.96)	97.3	0.06 (0.05 to 0.06)
Central Latin America	24.4	61279.73 (61254.47 to 61305)	42.16	61918.1 (61899.4 to 61936.8)	72.8	0.03 (0.02 to 0.04)
Southern Latin America	7.3	59972.29 (59928.64 to 60015.98)	10.52	59786.57 (59750.41 to 59822.75)	44.17	−0.03 (−0.04 to −0.02)
Tropical Latin America	23.67	60891.43 (60866.4 to 60916.47)	36.9	59981.3 (59961.88 to 60000.73)	55.87	−0.09 (−0.11 to −0.08)
North Africa and Middle East	50.53	70395.98 (70375.89 to 70416.06)	112.46	70779.89 (70766.79 to 70792.99)	122.58	0 (−0.01 to 0.01)
High-income North America	39.82	52827.14 (52810.65 to 52843.63)	48.12	56500.05 (56484.04 to 56516.06)	20.85	0.2 (0.13 to 0.27)
Oceania	0.8	54130.31 (54007.82 to 54253.05)	1.85	54277.23 (54198.37 to 54356.19)	130.43	0.01 (0 to 0.02)
Central Sub-Saharan Africa	7.2	63225.61 (63177.47 to 63273.77)	19.55	64196.44 (64167.11 to 64225.77)	171.63	0.04 (0.01 to 0.06)
Eastern Sub-Saharan Africa	24.65	62167.93 (62142.07 to 62193.81)	63.32	63253.37 (63237.18 to 63269.57)	156.9	0.06 (0.05 to 0.07)
Southern Sub-Saharan Africa	8.21	66313.92 (66266.79 to 66361.08)	14.18	65917.23 (65882.77 to 65951.71)	72.72	−0.01 (−0.02 to 0.01)
Western Sub-Saharan Africa	24.34	61480.14 (61454.45 to 61505.83)	68.07	61694.34 (61679.16 to 61709.54)	179.63	0 (−0.02 to 0.02)

**Fig 3:**
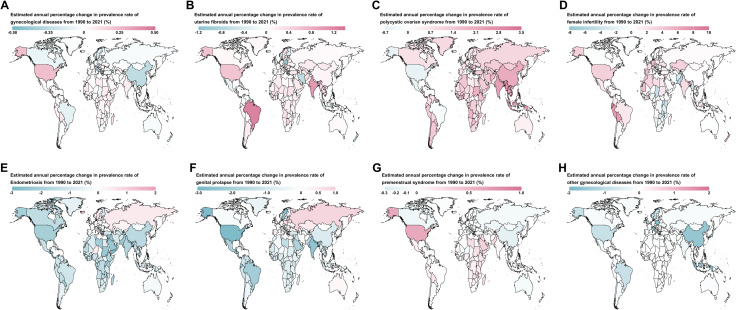
Estimated annual percentage change in age-standardized prevalence rates of gynecological diseases across 204 countries and territories in 2021. **(A)** Overall gynecological diseases. **(B)** Uterine fibroids. **(C)** Polycystic ovarian syndrome. **(D)** Female infertility. **(E)** Endometriosis. **(F)** Genital prolapses. **(G)** Premenstrual syndrome. **(H)** Other gynecological diseases.

Between 1990 and 2021, the global number of prevalent cases increased by 51.96%, while the ASPR remained stable (EAPC = 0; 95% CI: –0.03 to 0.02) (Table 1). At the regional level, the most significant ASPR increase was observed in High-income North America, with an average annual rise of 0.2% (95% CI: 0.13 to 0.27) (Fig 1B). Among the 204 countries and territories analyzed, 106 exhibited an increasing ASPR trend (Fig 3A, S4 Table in S1 File), with Taiwan experiencing the most pronounced rise (EAPC = 0.3; 95% CI: 0.23 to 0.37), followed by the Philippines (EAPC = 0.28 [95%CI: 0.22 to 0.34]).

### The prevalence of specific GDs

In 2021, the global number of prevalent cases among WCBA was estimated at 85.18 million for uterine fibroids, 65.77 million for PCOS, 110.09 million for female infertility, 21.05 million for endometriosis, 32.02 million for genital prolapse, 889.97 million for PMS, and 400.68 million for other GDs (Table 1). Correspondingly, their ASPR per 100,000 population were as follows: uterine fibroids, 4,352.34 (95% UI: 4,351.41 to 4,353.26); PCOS, 3,372.56 (95% UI: 3,371.75 to 3,373.38); female infertility, 5,636.81 (95% UI: 5,635.76 to 5,637.86); endometriosis, 1,077.83 (95% UI: 1,077.37 to 1,078.29); genital prolapse, 1,634.39 (95% UI: 1,633.82 to 1,634.96); PMS, 45,637.68 (95% UI: 45,634.68 to 45,640.68); and other GDs, 20,492.62 (95% UI: 20,490.61–20,494.62). Additionally, PMS accounted for the highest proportion of all prevalent cases of GDs across the 21 GBD regions, except for North Africa and the Middle East (Fig 1D). From 1990 to 2021, the global ASPR for uterine fibroids, PCOS, and female infertility showed an increasing trend. In contrast, the global ASPR for endometriosis, genital prolapse, and other GDs declined, while the global ASPR for PMS remained stable (Table 1).

In 2021, regionally, the highest ASPR per 100,000 population for each GDs was reported as follows: uterine fibroids in Eastern Europe (10268.73 [95% UI: 10260.39 to 10277.07]) (S5 Table in S1 File), PCOS in High-income Asia Pacific (10140.46 [95% UI: 10130.07 to 10150.87]) (S6 Table in S1 File), female infertility in East Asia (8420.38 [95% UI: 8417.34 to 8423.42]) (S7 Table in S1 File), endometriosis in Oceania (1921.6 [95% UI: 1906.74 to 1936.56]) (S8 Table in S1 File), genital prolapse in Tropical Latin America (3614.03 [95% UI: 3609.37 to 3618.71]) (S9 Table in S1 File), PMS in South Asia (50420.87 [95% UI: 50414.57 to 50427.18]) (S10 Table in S1 File), and other GDs in North Africa and the Middle East (48113.93 [95% UI: 48103.11 to 48124.76]) (S11 Table in S1 File). From 1990 to 2021, among the 21 regions, the highest ASPR were observed in Tropical Latin America for uterine fibroids (EAPC = 1.21 [95% CI: 1.12 to 1.31]), Southeast Asia for PCOS (EAPC = 2.3 [95% CI: 2.19 to 2.4]), Andean Latin America for female infertility (EAPC = 8.21 [95% CI: 6.69 to 9.76]), Eastern Europe for endometriosis (EAPC = 0.33 [95% CI: 0.15 to 0.5]), Eastern Europe for genital prolapse (EAPC = 0.68 [95% CI: 0.28 to 1.09]), High-income North America for PMS (EAPC = 0.53 [95% CI: 0.45 to 0.62]), and Eastern Sub-Saharan Africa for other gynecological diseases (EAPC = 0.03 [95% CI: 0.01 to 0.04]).

Among the 204 countries and territories, the highest ASPR per 100,000 population for each GDs in 2021 were as follows: Latvia for uterine fibroids (11729.95 [95% UI: 11630.98 to 11829.72]) (Fig 2B, S12 Table in S1 File), Italy for PCOS (15308.01 [95% UI: 15285.15 to 15330.9]) (Fig 2C, S13 Table in S1 File), Central African Republic for female infertility (11739.3 [95% UI: 11678.7 to 11800.17]) (Fig 2D, S14 Table in S1 File), Niger for endometriosis (2520.75 [95% UI: 2505.72 to 2535.86]) (Fig 2E, S15 Table in S1 File), Paraguay for genital prolapse (4480.37 [95% UI: 4448.92 to 4512]) (Fig 2F, S16 Table in S1 File), Pakistan for PMS (51547.67 [95% UI: 51529.23 to 51566.12]) (Fig 2G, S17 Table in S1 File), and Yemen for other gynecological diseases (49967.45 [95% UI: 49915.67 to 50019.28]) (Fig 2H, S18 Table in S1 File). Between 1990 and 2021, the fastest increases in ASPR were observed in Brazil for uterine fibroids (EAPC = 1.23 [95% CI: 1.14 to 1.33]) (Fig 3B), Maldives for PCOS (EAPC = 3.39 [95% CI: 3.11 to 3.66]) (Fig 3C), Ecuador for female infertility (EAPC = 9.35 [95% CI: 7.3 to 11.44]) (Fig 3D), Iceland for endometriosis (EAPC = 1.19 [95% CI: 0.92 to 1.46]) (Fig 3E), Russia for genital prolapse (EAPC = 0.88 [95% CI: 0.45 to 1.31]) (Fig 3F), United States for PMS (EAPC = 0.61 [95% CI: 0.52 to 0.71]) (Fig 3G), and Taiwan for other gynecological diseases (EAPC = 1.18 [95% CI: 0.87 to 1.5]) (Fig 3H).

### The correlation between ASPR of GDs and SDI

**Fig 4:**
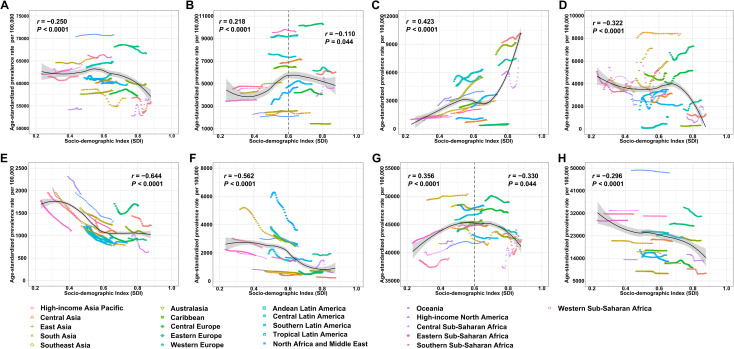
Age-standardized prevalence rates of gynecological diseases across 21 GBD regions, by SDI, from 1990 to 2021. **(A)** Overall gynecological diseases. **(B)** Uterine fibroids. **(C)** Polycystic ovarian syndrome. **(D)** Female infertility. **(E)** Endometriosis. **(F)** Genital prolapses. **(G)** Premenstrual syndrome. **(H)** Other gynecological diseases. The solid line represents the expected values based on SDI and prevalence rates across all locations. Each region is represented by 32 points, showing the observed age-standardized prevalence rate for each year from 1990 to 2021. The shaded area indicates the 95% confidence interval (CI) of the expected values. Points above the solid line indicate a higher-than-expected prevalence, while those below the line represent a lower-than-expected prevalence. GBD = Global Burden of Diseases, Injuries, and Risk Factors Study. SDI = Socio-demographic Index.

At the regional level, the ASPR of overall GDs remained stable with increasing SDI initially, but began to decline when the SDI reached 0.55 (Fig 4A). Between 1990 and 2021, regions such as North Africa and the Middle East, Eastern Europe, and Western Europe showed higher-than-expected ASPRs for GDs based on their SDI.

For specific GDs, the ASPR of uterine fibroids and PMS both initially increased with rising SDI but began to decline once the SDI reached 0.60 (Fig 4B and 4G). In contrast, the ASPR for PCOS increased exponentially with higher SDI (Fig 4C). However, the ASPRs for female infertility, endometriosis, genital prolapse, and other GDs all showed a decreasing trend as SDI increased (Fig 4D, 4E, 4F, and 4H).

### Age-patterns in the prevalence of GDs

**Fig 5:**
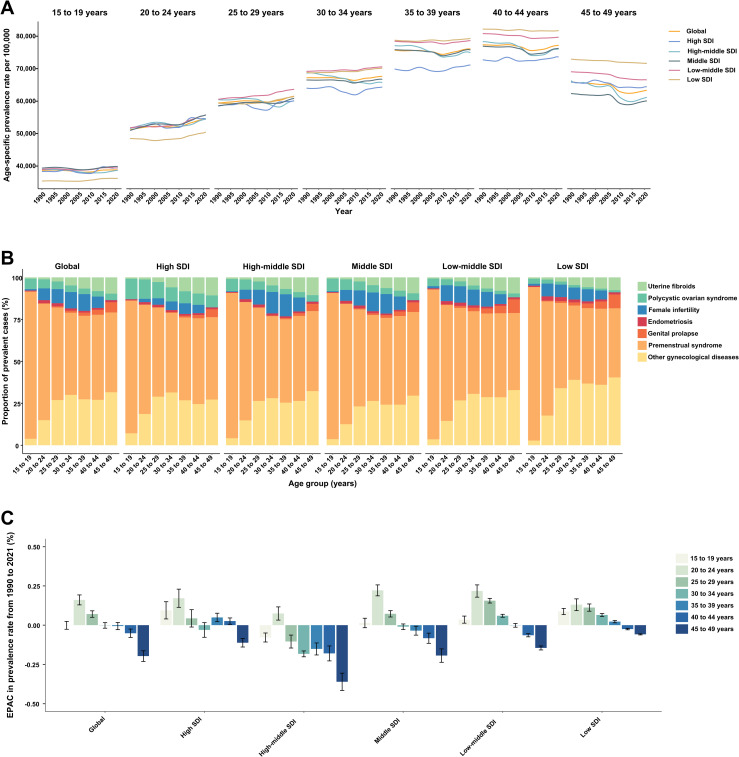
Prevalence and trends of gynecological diseases (GDs) among women of childbearing age, by age group and SDI. **(A)** Prevalence rates of GDs globally and across the 5 SDI regions from 1990 to 2021, stratified by age group. **(B)** Proportions of prevalent cases attributed to each GD within each age group across the globe and the 5 SDI regions in 2021. **(C)** Estimated annual percentage changes in the prevalence rate of GDs from 1990 to 2021, categorized by age group and SDI. SDI = Socio-demographic index.

Among WCBA, the prevalence rate of overall GDs generally increased with age, peaking in the 40–44 years age group (Fig 5A). Interestingly, for women aged 15–19 and 20–24 years, regions with a middle SDI had the highest prevalence rate of overall GDs. In contrast, for women aged 25–29 and 30–34 years, the highest prevalence rate was observed in low-middle-SDI regions. For women aged 35–39, 40–44, and 45–49 years, the highest prevalence rates were found in low-SDI regions. Globally, as well as across the five SDI quintiles, the contribution of PMS and PCOS to overall GDs decreased with age, whereas the contribution of uterine fibroids increased with age (Fig 5B).

**Fig 6:**
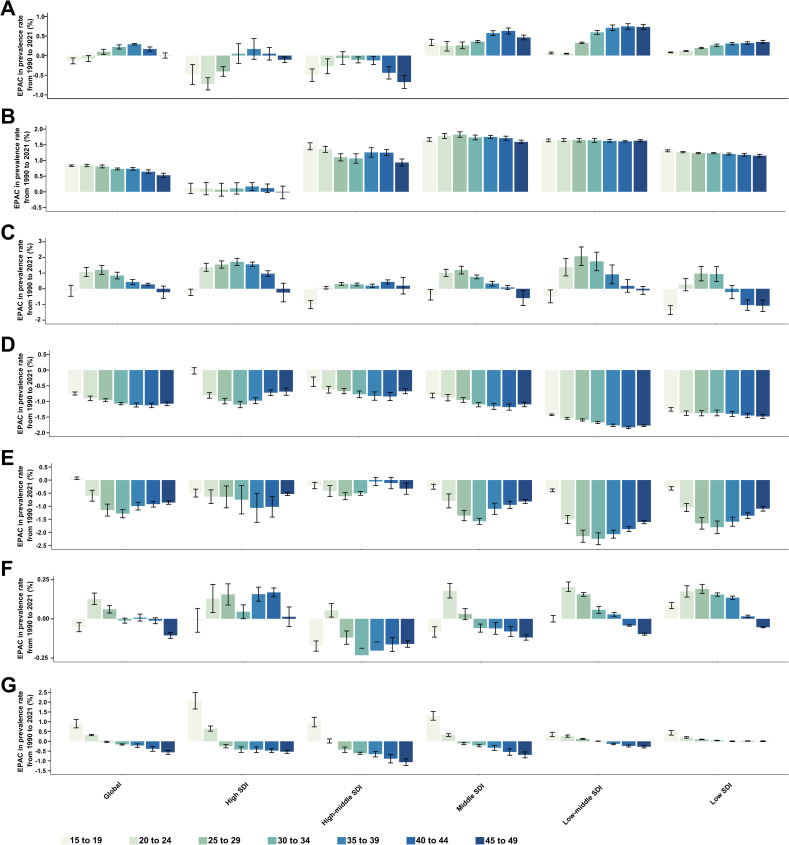
Estimated annual percentage changes in the prevalence rate of specific gynecological diseases from 1990 to 2021, categorized by age group and SDI. **(A)** Uterine fibroids. **(B)** Polycystic ovarian syndrome. **(C)** Female infertility. **(D)** Endometriosis. **(E)** Genital prolapses. **(F)** Premenstrual syndrome. **(G)** Other gynecological diseases. SDI = Socio-demographic index.

From 1990 to 2021, the most significant increase in prevalence rates for overall GDs occurred in the 20–24 years age group, while the largest decrease was observed in the 45–49 years age group (Fig 5C). These trends indicate that the burden of GDs is increasingly affecting younger WCBA. Specifically, the fastest increase in prevalence rates was seen for uterine fibroids in women aged 40–44 years, PCOS in women aged 15–19 years, female infertility in women aged 25–29 years, PMS in women aged 20–24 years, and other GDs in women aged 15–19 years (Fig 6). In contrast, the prevalence rates of endometriosis and genital prolapse either decreased or remained stable across all age groups among women of childbearing age (Fig 6D and 6E).

## Discussion

This study offers a comprehensive analysis of the global prevalence and temporal trends of GDs among WCBA, using data from the GBD 2021. The key findings are as follows: First, ASPR of GDs remained stable, but the total number of GD cases increased by 51.96% from 1990 to 2021, reflecting population growth and aging. Second, (PMS was the most prevalent GD globally, followed by uterine fibroids, PCOS, and female infertility. Third, the ASPR of uterine fibroids, PCOS, and female infertility increased over time, particularly in low- and low-middle SDI regions, while rates for endometriosis, genital prolapse, and PMS remained stable or declined. Fourth, the association between GD prevalence and SDI showed a threshold effect: prevalence remained stable up to a certain SDI level, then declined with higher SDI. Fifth, uterine fibroids and PMS increased initially but plateaued in middle-SDI regions, whereas female infertility, endometriosis, and genital prolapse showed a consistent decline with rising SDI. Finally, GD prevalence peaked in the 40–44 age group, but a concerning shift toward younger women (ages 20–29) was observed, particularly for uterine fibroids, PCOS, female infertility, and PMS. These findings underscore the growing and uneven burden of GDs worldwide and highlight the urgent need for targeted public health strategies to address age- and region-specific disparities in women’s reproductive health.

### Temporal trend of overall GDs

Notably, although the global ASPR of GDs remained relatively stable from 1990 to 2021, the absolute number of prevalent cases increased substantially. This divergence is largely attributable to global population growth and shifts in age structure, particularly the expansion of the population of WCBA in low- and middle-income countries [30]. ASPR adjusts for changes in population age composition and allows for valid comparisons over time and across regions, serving as an indicator of true changes in disease risk [25]. In contrast, absolute case numbers are directly influenced by demographic trends, including increases in total population size and aging. As reported by the GBD 2021 study, such demographic shifts continue to drive the rising burden of non-fatal conditions worldwide, even when age-standardized rates appear stable or decline [31]. Therefore, our findings underscore the importance of considering both absolute and relative measures when interpreting disease burden and formulating policy responses for women’s reproductive health.

### Temporal trend of specific GDs

#### Premenstrual syndrome.

Our results show that PMS affected 45.6% of WCBA worldwide in 2021, aligning with previous reports indicating that PMS impacts nearly half of reproductive women globally [32]. The prevalence rate among WCBA was significantly higher compared to the general female population (27.9%) [10]. Additionally, PMS accounted for the largest proportion of GDs in most regions, with the number of prevalent cases nearly doubling from 1990 to 2021, despite the ASPR remained stable over this period. The stable ASPR for PMS suggests that, although the absolute number of cases has likely increased due to population growth, the prevalence rate of PMS relative to age has not changed significantly. Notably, the pathophysiology of PMS is primarily linked to cyclical hormonal fluctuations rather than modifiable risk factors such as smoking or obesity [33]. Although lifestyle and environmental factors like diet, physical activity, and stress levels have evolved over the past decades, these changes may have influenced the severity of PMS symptoms rather than its overall prevalence [34–36]. Addressing these modifiable risk factors through weight management, smoking cessation, and lifestyle changes could potentially help alleviate PMS symptoms and improve women’s overall reproductive health.

#### Polycystic ovarian syndrome.

PCOS is the most common endocrine disorder among WCBA, affecting approximately 10–18% of this population [37]. Among major GDs, PCOS showed the fastest increase in ASPR, particularly in middle- and low-middle-SDI regions, including East, South, and Southeast Asia. Several studies from these areas confirm this rising trend. For example, two national surveys in China showed that the prevalence of PCOS among women aged 20–44 increased by two-thirds between 2010 and 2020 [38]. A similar increase has been observed in India [39]. This rise may be partially attributed to lifestyle changes associated with urbanization, such as diets high in processed foods, reduced physical activity, and increased stress [40]. These factors are associated with higher rates of metabolic disorders, particularly insulin resistance—a key feature of PCOS [41]. Demographic changes, including delayed childbearing and increased reproductive health challenges among career-oriented women, may also contribute to the upward trend in PCOS prevalence [42,43]. Since PCOS is closely associated with infertility [44], these trends may be compounding its impact. Our findings, along with prior studies, also highlight a rising prevalence of female infertility, especially in high-SDI regions, where the increase has been most rapid [4,45]. Notably, ASPR for PCOS tends to rise with increasing SDI levels. While high-SDI regions may show higher rates due to better detection and healthcare access, underdiagnosis may still be common in low-SDI regions due to limited healthcare availability [46].

#### Uterine fibroids.

From 1990 to 2021, we also observed an overall global increase in the ASPR of uterine fibroids. However, this trend varied by development level: rates decreased in high- and high-middle-SDI regions, while increasing in middle-, low-middle-, and low-SDI regions. In high-SDI regions, widespread use of advanced diagnostic tools (e.g., ultrasound, MRI) and access to minimally invasive treatments (e.g., laparoscopic myomectomy, uterine artery embolization) may contribute to reducing long-term prevalence [47–50]. Additionally, hormonal therapies and intrauterine devices (IUDs) are commonly used to manage or suppress fibroid growth [51]. In contrast, limited access to these diagnostic and therapeutic options in lower-SDI regions may lead to persistent or recurrent fibroids, increasing disease burden [52,53]. Urbanization in developing regions has also brought lifestyle shifts—such as sedentary behavior, processed diets, and rising obesity—that are recognized risk factors for fibroids [54]. While historically higher fertility rates in low-SDI areas offered some protection, declining birth rates may reduce this effect [55,56]. Moreover, growing exposure to environmental toxins (e.g., phthalates, pesticides) in industrializing areas may interfere with hormonal regulation and promote fibroid development [57].

#### Endometriosis.

From 1990 to 2021, the ASPR of endometriosis declined globally and in most GBD regions, except in Eastern Europe. This overall decrease likely reflects improvements in healthcare access, diagnostic accuracy, and preventive care [58]. In contrast, the upward trend in Eastern Europe may be attributed to several region-specific challenges. Following post-Soviet industrialization, weak environmental regulations led to high exposure to endocrine-disrupting chemicals—such as polycyclic aromatic hydrocarbons—which have been linked to the development of endometriosis [59]. The economic upheavals of the 1990s also disrupted funding for women’s health services, reducing access to diagnostic tools and treatments [60]. Additionally, the migration of skilled gynecologists and radiologists to Western Europe further strained local healthcare systems, resulting in poorer quality care [61]. Lifestyle factors such as high rates of smoking, alcohol use, and industrial pollution further contribute to hormonal imbalances and inflammation, both of which are associated with endometriosis [62,63]. Cultural attitudes in some Eastern European countries may also play a role, as societal norms that downplay menstrual or pelvic pain can lead to delayed diagnosis and untreated cases [64]. Notably, endometriosis is a complex, chronic condition associated not only with reproductive health concerns such as infertility and dysmenorrhea, but also with a wide range of non-reproductive symptoms, including chronic pelvic pain, gastrointestinal disturbances, fatigue, and dyspareunia [65]. These symptoms significantly impair physical, emotional, sexual, and social well-being, and contribute to a substantial disease burden that extends beyond fertility-related issues.

#### Genital prolapse.

The epidemiological trends of genital prolapse closely resemble those of endometriosis. Advances in minimally invasive surgical techniques, such as laparoscopic or robotic-assisted pelvic surgery, have led to significant improvements in both surgical outcomes and recovery times for women with genital prolapse [66]. Improved imaging tools such as pelvic ultrasound and MRI have enhanced early detection and more accurate assessment of prolapse severity, allowing for timely and effective interventions [67]. The development of urogynecology as a specialized field has also expanded access to expert care in regions with stronger healthcare infrastructure [68]. Efforts to improve maternal health—especially in preventing childbirth-related injuries that contribute to pelvic floor dysfunction—have helped reduce prolapse rates [69]. Measures such as minimizing prolonged labor, limiting unnecessary episiotomies, and managing traumatic deliveries have played a key role. Additionally, promoting postnatal pelvic floor exercises, such as Kegel exercises, may further lower the risk and severity of prolapse [70]. As rates of vaginal delivery decline in countries with effective family planning, the incidence of pelvic organ prolapse is likely to continue decreasing [71].

### Implications for younger women

This study emphasizes that the high and persistent burden of gynecological diseases among WCBA calls for urgent policy attention [72]. Health systems, particularly in low- and middle-income countries, should prioritize the integration of gynecological diseases prevention, early detection, and treatment services into primary and reproductive health care programs [73,74]. Targeted screening, affordable access to diagnostics and medications, and public awareness campaigns could help reduce diagnostic delays and treatment gaps. Furthermore, policy-makers should allocate sufficient resources to women’s health, establish standardized care pathways for common gynecological diseases such as PCOS, endometriosis, and uterine fibroids, and promote research and data monitoring to inform evidence-based interventions [75].

The prevalence of GDs among WCBA increases with age; however, the most significant rise from 1990 to 2021 was observed in younger age groups. Specifically, uterine fibroids, PCOS, female infertility, and PMS were most prevalent in women aged 20–29 years. Considering the observed increasing burden of GDs among younger women, particularly in low- and middle-SDI regions, targeted public health strategies are urgently needed. In these regions, improving access to primary gynecological care—through investments in community-level services, early screening programs, and health insurance coverage—can facilitate timely diagnosis and management [76]. School- and university-based reproductive health education should be implemented to improve awareness, reduce stigma, and encourage early health-seeking behaviors among adolescent and young adult women [77]. In high-SDI settings, age-appropriate interventions should prioritize modifiable risk factors such as obesity, sedentary behavior, and delayed childbearing [78]. Preventive measures, including lifestyle counseling and routine screening for hormonal and reproductive disorders, should be integrated into women’s health services starting in early adulthood.

### Limitations

The current study has several limitations. First, many gynecological diseases, such as endometriosis, PCOS, and uterine fibroids, often present with mild or nonspecific symptoms in their early stages and may go undiagnosed for long periods [79,80]. As a result, the true burden of these conditions is likely underestimated, especially in settings with limited access to gynecological care or routine screening. Second, there are substantial regional disparities in data availability and quality. In particular, low-SDI countries frequently lack comprehensive surveillance systems and high-quality administrative health records, resulting in large data gaps [81]. Although the GBD 2021 study uses robust Bayesian modeling methods (e.g., DisMod-MR 2.1) to generate estimates in data-sparse regions, the accuracy of these modeled outputs depends on the availability and representativeness of the primary data [1]. Inconsistencies in diagnostic criteria, coding practices, and reporting standards across countries may further affect the comparability of results [82]. Therefore, while our study provides a comprehensive overview of GD burden globally, the estimates—particularly in low-resource settings—should be interpreted with caution.

## Conclusion

In conclusion, GDs continue to impose a substantial and increasing health burden on WCBA worldwide, with significant disparities observed across regions and socio-demographic levels. Policymakers and healthcare systems must prioritize the development of accessible, equitable, and culturally sensitive services, especially in low- and middle-income regions where access remains limited. Enhancing public awareness, expanding routine screening programs, and investing in women’s health research are critical steps toward mitigating this burden. Strengthening primary care and referral systems for timely intervention can play a pivotal role in improving outcomes and quality of life.

## Supporting information

S1 File S1 Table. 21 GBD world regions and 204 countries and territories within each region. S2 Table. Socio-demographic Index (SDI) quintiles for 204 countries and territories estimated in GBD 2021. S3 Table. Case definitions and mapping of International Classification of Diseases (ICD) codes to gynecological diseases in GBD 2021. S4 Table. The global prevalence of gynecological diseases among women aged 15–49 years in 1990 and 2021, along with the trends and changes observed between these years, by country and territories. S5 Table. The global prevalence of uterine fibroids among women aged 15–49 years in 1990 and 2021, along with the trends and changes observed between these years. S6 Table. The global prevalence of polycystic ovarian syndrome among women aged 15–49 years in 1990 and 2021, along with the trends and changes observed between these years. S7 Table. The global prevalence of female infertility among women aged 15–49 years in 1990 and 2021, along with the trends and changes observed between these years. S8 Table. The global prevalence of endometriosis among women aged 15–49 years in 1990 and 2021, along with the trends and changes observed between these years. S9 Table. The global prevalence of genital prolapses among women aged 15–49 years in 1990 and 2021, along with the trends and changes observed between these years. S10 Table. The global prevalence of premenstrual syndrome among women aged 15–49 years in 1990 and 2021, along with the trends and changes observed between these years. S11 Table. The global prevalence of other gynecological diseases among women aged 15–49 years in 1990 and 2021, along with the trends and changes observed between these years. S12 Table. The global prevalence of uterine fibroids among women aged 15–49 years in 1990 and 2021, along with the trends and changes observed between these years, by country and territories. S13 Table. The global prevalence of polycystic ovarian syndrome among women aged 15–49 years in 1990 and 2021, along with the trends and changes observed between these years, by country and territories. S14 Table. The global prevalence of female infertility among women aged 15–49 years in 1990 and 2021, along with the trends and changes observed between these years, by country and territories. S15 Table. The global prevalence of endometriosis among women aged 15–49 years in 1990 and 2021, along with the trends and changes observed between these years, by country and territories. S16 Table. The global prevalence of genital prolapses among women aged 15–49 years in 1990 and 2021, along with the trends and changes observed between these years, by country and territories. S17 Table. The global prevalence of premenstrual syndrome among women aged 15–49 years in 1990 and 2021, along with the trends and changes observed between these years, by country and territories. S18 Table. The global prevalence of other gynecological diseases among women aged 15–49 years in 1990 and 2021, along with the trends and changes observed between these years, by country and territories.
(PDF)

## References

[1] GBD 2021 Diseases and Injuries Collaborators. Global incidence, prevalence, years lived with disability (YLDs), disability-adjusted life-years (DALYs), and healthy life expectancy (HALE) for 371 diseases and injuries in 204 countries and territories and 811 subnational locations, 1990-2021: a systematic analysis for the Global Burden of Disease Study 2021. Lancet. 2024;403(10440):2133–61. doi: 10.1016/S0140-6736(24)00757-8 38642570 PMC11122111

[2] Taylor HS, Kotlyar AM, Flores VA. Endometriosis is a chronic systemic disease: clinical challenges and novel innovations. Lancet. 2021;397(10276):839–52. doi: 10.1016/S0140-6736(21)00389-5 33640070

[3] Harmon QE. The burden of uterine fibroids: a search for primary and secondary prevention. Fertil Steril. 2019;111(1):50–1. doi: 10.1016/j.fertnstert.2018.10.031 30611415

[4] Cox CM, Thoma ME, Tchangalova N, Mburu G, Bornstein MJ, Johnson CL, et al. Infertility prevalence and the methods of estimation from 1990 to 2021: a systematic review and meta-analysis. Hum Reprod Open. 2022;2022(4):hoac051. doi: 10.1093/hropen/hoac051 36483694 PMC9725182

[5] Cao Y, Guo Y, Long Z, Wu Y, Pei B, Ye J, et al. The global burden of gynecological diseases from 1990 to 2019. Am J Prev Med. 2024;67(5):698–704. doi: 10.1016/j.amepre.2024.06.022 38945179

[6] The Lancet. Endometriosis: addressing the roots of slow progress. Lancet. 2024;404(10460):1279. doi: 10.1016/S0140-6736(24)02179-2 39368831

[7] Vannuccini S, Clifton VL, Fraser IS, Taylor HS, Critchley H, Giudice LC, et al. Infertility and reproductive disorders: impact of hormonal and inflammatory mechanisms on pregnancy outcome. Hum Reprod Update. 2016;22(1):104–15. doi: 10.1093/humupd/dmv044 26395640 PMC7289323

[8] Dong Y, Chen M, Wu Y. Effects of social norms and message framing on reducing the stigma of gynecological diseases: a cognitive-affective-behavioral model. Appl Psychol Health Well Being. 2023;15(4):1221–36. doi: 10.1111/aphw.12433 36539383

[9] Pynnä K, Räsänen P, Roine RP, Vuorela P, Sintonen H. Where does the money go to? Cost analysis of gynecological patients with a benign condition. PLoS One. 2021;16(7):e0254124. doi: 10.1371/journal.pone.0254124 34242306 PMC8270439

[10] Liu X, Li R, Wang S, Zhang J. Global, regional, and national burden of premenstrual syndrome, 1990-2019: an analysis based on the global burden of disease study 2019. Hum Reprod. 2024;39(6):1303–15. doi: 10.1093/humrep/deae081 38689567

[11] Motlagh Asghari K, Nejadghaderi SA, Alizadeh M, Sanaie S, Sullman MJM, Kolahi A-A, et al. Burden of polycystic ovary syndrome in the Middle East and North Africa region, 1990-2019. Sci Rep. 2022;12(1):7039. doi: 10.1038/s41598-022-11006-0 35488014 PMC9052181

[12] Lou Z, Huang Y, Li S, Luo Z, Li C, Chu K, et al. Global, regional, and national time trends in incidence, prevalence, years lived with disability for uterine fibroids, 1990-2019: an age-period-cohort analysis for the global burden of disease 2019 study. BMC Public Health. 2023;23(1):916. doi: 10.1186/s12889-023-15765-x 37208621 PMC10199532

[13] Dioikitopoulos E, Varvarigos D. Delay in childbearing and the evolution of fertility rates. J Popul Econ. 2023;36(3):1545–71. doi: 10.1007/s00148-022-00931-z

[14] Huang J, Huang JL, Withers M, Chien K-L, Trihandini I, Elcarte E, et al. Prevalence of metabolic syndrome in Chinese women and men: a systematic review and meta-analysis of data from 734 511 individuals. The Lancet. 2018;392:S14. doi: 10.1016/s0140-6736(18)32643-6

[15] Hirode G, Wong RJ. Trends in the prevalence of metabolic syndrome in the United States, 2011-2016. JAMA. 2020;323(24):2526–8. doi: 10.1001/jama.2020.4501 32573660 PMC7312413

[16] Johansson HKL, Svingen T, Fowler PA, Vinggaard AM, Boberg J. Environmental influences on ovarian dysgenesis - developmental windows sensitive to chemical exposures. Nat Rev Endocrinol. 2017;13(7):400–14. doi: 10.1038/nrendo.2017.36 28450750

[17] GBD 2021 Forecasting Collaborators. Burden of disease scenarios for 204 countries and territories, 2022-2050: a forecasting analysis for the global burden of disease study 2021. Lancet. 2024;403(10440):2204–56. doi: 10.1016/S0140-6736(24)00685-8 38762325 PMC11121021

[18] The Lancet. A broader vision for women’s health. Lancet. 2023;402(10399):347. doi: 10.1016/S0140-6736(23)01570-2 37516532

[19] Gupta S, Jose J, Manyonda I. Clinical presentation of fibroids. Best Pract Res Clin Obstet Gynaecol. 2008;22(4):615–26. doi: 10.1016/j.bpobgyn.2008.01.008 18372219

[20] Martire FG, Russo C, Selntigia A, Nocita E, Soreca G, Lazzeri L, et al. Early noninvasive diagnosis of endometriosis: dysmenorrhea and specific ultrasound findings are important indicators in young women. Fertil Steril. 2023;119(3):455–64. doi: 10.1016/j.fertnstert.2022.12.004 36493871

[21] Murray CJL. The global burden of disease study at 30 years. Nat Med. 2022;28(10):2019–26. doi: 10.1038/s41591-022-01990-1 36216939

[22] Cao F, Li D-P, Wu G-C, He Y-S, Liu Y-C, Hou J-J, et al. Global, regional and national temporal trends in prevalence for musculoskeletal disorders in women of childbearing age, 1990-2019: an age-period-cohort analysis based on the global burden of disease study 2019. Ann Rheum Dis. 2024;83(1):121–32. doi: 10.1136/ard-2023-224530 37666645

[23] Dai X, Tao Y, Zhou J, Zhou Y, Liang S, Ma X. Global burden and trends of severe periodontitis among women of childbearing age, 1990-2021. J Periodontol. 2025. doi: 10.1002/JPER.24-0615 39868976

[24] GBD 2021 Demographics Collaborators. Global age-sex-specific mortality, life expectancy, and population estimates in 204 countries and territories and 811 subnational locations, 1950-2021, and the impact of the COVID-19 pandemic: a comprehensive demographic analysis for the global burden of disease study 2021. Lancet. 2024;403(10440):1989–2056. doi: 10.1016/S0140-6736(24)00476-8 38484753 PMC11126395

[25] Naing NN. Easy way to learn standardization: direct and indirect methods. Malays J Med Sci. 2000;7(1):10–5. 22844209 PMC3406211

[26] Hankey BF, Ries LA, Kosary CL, Feuer EJ, Merrill RM, Clegg LX, et al. Partitioning linear trends in age-adjusted rates. Cancer Causes Control. 2000;11(1):31–5. doi: 10.1023/a:1008953201688 10680727

[27] Fay MP, Tiwari RC, Feuer EJ, Zou Z. Estimating average annual percent change for disease rates without assuming constant change. Biometrics. 2006;62(3):847–54. doi: 10.1111/j.1541-0420.2006.00528.x 16984328

[28] Cleveland WS. Robust locally weighted regression and smoothing scatterplots. J Am Statist Assoc. 1979;74(368):829–36. doi: 10.1080/01621459.1979.10481038

[29] Jacoby WG. Loess: a nonparametric, graphical tool for depicting relationships between variables. Elect Stud. 2000;19(4):577–613. doi: 10.1016/s0261-3794(99)00028-1

[30] Vollset SE, Goren E, Yuan C-W, Cao J, Smith AE, Hsiao T, et al. Fertility, mortality, migration, and population scenarios for 195 countries and territories from 2017 to 2100: a forecasting analysis for the global burden of disease study. Lancet. 2020;396(10258):1285–306. doi: 10.1016/S0140-6736(20)30677-2 32679112 PMC7561721

[31] Hambleton IR, Caixeta R, Jeyaseelan SM, Luciani S, Hennis AJM. The rising burden of non-communicable diseases in the Americas and the impact of population aging: a secondary analysis of available data. Lancet Reg Health Am. 2023;21:100483. doi: 10.1016/j.lana.2023.100483 37065858 PMC10090658

[32] A D-M, K S, A D, Sattar K. Epidemiology of premenstrual syndrome (PMS)-a systematic review and meta-analysis study. J Clin Diagn Res. 2014;8(2):106–9. doi: 10.7860/JCDR/2014/8024.4021 24701496 PMC3972521

[33] Yonkers KA, Simoni MK. Premenstrual disorders. Am J Obstet Gynecol. 2018;218(1):68–74. doi: 10.1016/j.ajog.2017.05.045 28571724

[34] Akseer N, Al-Gashm S, Mehta S, Mokdad A, Bhutta ZA. Global and regional trends in the nutritional status of young people: a critical and neglected age group. Ann N Y Acad Sci. 2017;1393(1):3–20. doi: 10.1111/nyas.13336 28436100

[35] Strain T, Flaxman S, Guthold R, Semenova E, Cowan M, Riley LM, et al. National, regional, and global trends in insufficient physical activity among adults from 2000 to 2022: a pooled analysis of 507 population-based surveys with 5·7 million participants. Lancet Glob Health. 2024;12(8):e1232–43. doi: 10.1016/S2214-109X(24)00150-5 38942042 PMC11254784

[36] Hashim MS, Obaideen AA, Jahrami HA, Radwan H, Hamad HJ, Owais AA, et al. Premenstrual syndrome is associated with dietary and lifestyle behaviors among university students: a cross-sectional study from Sharjah, UAE. Nutrients. 2019;11(8):1939. doi: 10.3390/nu11081939 31426498 PMC6723319

[37] Morin-Papunen L. Bariatric surgery in women with PCOS and obesity. Lancet. 2024;403(10443):2456–7. doi: 10.1016/S0140-6736(24)00811-0 38782002

[38] Yang R, Li Q, Zhou Z, Qian W, Zhang J, Wu Z, et al. Changes in the prevalence of polycystic ovary syndrome in China over the past decade. Lancet Reg Health West Pac. 2022;25:100494. doi: 10.1016/j.lanwpc.2022.100494 35669932 PMC9162959

[39] Ganie MA, Chowdhury S, Suri V, Joshi B, Bhattacharya PK, Agrawal S, et al. Evaluation of the prevalence, regional phenotypic variation, comorbidities, risk factors, and variations in response to different therapeutic modalities among indian women: proposal for the indian council of medical research-polycystic ovary syndrome (ICMR-PCOS) study. JMIR Res Protoc. 2021;10(8):e23437. doi: 10.2196/23437 34448720 PMC8433859

[40] Mu L, Zhao Y, Li R, Lai Y, Chang H-M, Qiao J. Prevalence of polycystic ovary syndrome in a metabolically healthy obese population. Int J Gynaecol Obstet. 2019;146(2):164–9. doi: 10.1002/ijgo.12824 31002378

[41] Gautam R, Maan P, Jyoti A, Kumar A, Malhotra N, Arora T. The role of lifestyle interventions in PCOS management: a systematic review. Nutrients. 2025;17(2):310. doi: 10.3390/nu17020310 39861440 PMC11767734

[42] Neels K, Murphy M, Ní Bhrolcháin M, Beaujouan É. Rising educational participation and the trend to later childbearing. Popul Dev Rev. 2017;43(4):667–93. doi: 10.1111/padr.12112 29398739 PMC5767733

[43] Frejka T, Jones GW, Sardon J-P. East Asian childbearing patterns and policy developments. Popul Dev Rev. 2010;36(3):579–606. doi: 10.1111/j.1728-4457.2010.00347.x 20882707

[44] Liu X, Zhang J, Wang S. Global, regional, and national burden of infertility attributable to PCOS, 1990-2019. Hum Reprod. 2024;39(1):108–18. doi: 10.1093/humrep/dead241 38011904

[45] Liang Y, Huang J, Zhao Q, Mo H, Su Z, Feng S, et al. Global, regional, and national prevalence and trends of infertility among individuals of reproductive age (15-49 years) from 1990 to 2021, with projections to 2040. Hum Reprod. 2025;40(3):529–44. doi: 10.1093/humrep/deae292 39752330

[46] Liu J, Wu Q, Hao Y, Jiao M, Wang X, Jiang S, et al. Measuring the global disease burden of polycystic ovary syndrome in 194 countries: global burden of disease study 2017. Hum Reprod. 2021;36(4):1108–19. doi: 10.1093/humrep/deaa371 33501984 PMC7970729

[47] Krishnan M, Narice B, Cheong YC, Lumsden MA, Daniels JP, Hickey M, et al. Surgery and minimally invasive treatments for uterine fibroids. Cochrane Database Syst Rev. 2024;6(6):CD015650. doi: 10.1002/14651858.CD015650 39804114 PMC11152210

[48] Hannemann B, McKnoulty M, Kothari A. Beware of a multi-fibroid uterus: The importance of ultrasound reporting in the early detection of uterine sarcomas. Australas J Ultrasound Med. 2016;19(4):154–9. doi: 10.1002/ajum.12030 34760461 PMC8409561

[49] Ceccaroni M, Ceccarello M, Raimondo I, Roviglione G, Clarizia R, Bruni F, et al. “A Space Odyssey” on laparoscopic segmental rectosigmoid resection for deep endometriosis: a seventeen-year retrospective analysis of outcomes and postoperative complications among 3050 patients treated in a referral center. J Minim Invasive Gynecol. 2023;30(8):652–64. doi: 10.1016/j.jmig.2023.04.005 37116746

[50] Raffone A, Raimondo D, Neola D, Travaglino A, Raspollini A, Giorgi M, et al. Diagnostic accuracy of ultrasound in the diagnosis of uterine leiomyomas and sarcomas. J Minim Invasive Gynecol. 2024;31(1):28-36.e1. doi: 10.1016/j.jmig.2023.09.013 37778636

[51] Mension E, Calaf J, Chapron C, Dolmans MM, Donnez J, Marcellin L, et al. An update on the management of uterine fibroids: personalized medicine or guidelines?. J Endometriosis Uterine Disord. 2024;7:100080. doi: 10.1016/j.jeud.2024.100080

[52] Frija G, Blažić I, Frush DP, Hierath M, Kawooya M, Donoso-Bach L, et al. How to improve access to medical imaging in low- and middle-income countries?. EClinicalMedicine. 2021;38:101034. doi: 10.1016/j.eclinm.2021.101034 34337368 PMC8318869

[53] Al-Hendy A, Myers ER, Stewart E. Uterine fibroids: burden and unmet medical need. Semin Reprod Med. 2017;35(6):473–80. doi: 10.1055/s-0037-1607264 29100234 PMC6193285

[54] Pavone D, Clemenza S, Sorbi F, Fambrini M, Petraglia F. Epidemiology and risk factors of uterine fibroids. Best Pract Res Clin Obstet Gynaecol. 2018;46:3–11. doi: 10.1016/j.bpobgyn.2017.09.004 29054502

[55] Laughlin SK, Herring AH, Savitz DA, Olshan AF, Fielding JR, Hartmann KE, et al. Pregnancy-related fibroid reduction. Fertil Steril. 2010;94(6):2421–3. doi: 10.1016/j.fertnstert.2010.03.035 20451187 PMC2927730

[56] GBD 2021 Fertility and Forecasting Collaborators. Global fertility in 204 countries and territories, 1950-2021, with forecasts to 2100: a comprehensive demographic analysis for the global burden of disease study 2021. Lancet. 2024;403(10440):2057–99. doi: 10.1016/S0140-6736(24)00550-6 38521087 PMC11122687

[57] Katz TA, Yang Q, Treviño LS, Walker CL, Al-Hendy A. Endocrine-disrupting chemicals and uterine fibroids. Fertil Steril. 2016;106(4):967–77. doi: 10.1016/j.fertnstert.2016.08.023 27553264 PMC5051569

[58] Saunders PTK, Whitaker LHR, Horne AW. Endometriosis: improvements and challenges in diagnosis and symptom management. Cell Rep Med. 2024;5(6):101596. doi: 10.1016/j.xcrm.2024.101596 38897171 PMC11228648

[59] Hemminki K, Veidebaum T. Environmental pollution and human exposure to polycyclic aromatic hydrocarbons in the east Baltic region. Scand J Work Environ Health. 1999;25 Suppl 3:33–9. 10546806

[60] Rechel B, McKee M. Health reform in central and eastern Europe and the former Soviet Union. Lancet. 2009;374(9696):1186–95. doi: 10.1016/S0140-6736(09)61334-9 19801097

[61] Toyin-Thomas P, Ikhurionan P, Omoyibo EE, Iwegim C, Ukueku AO, Okpere J, et al. Drivers of health workers’ migration, intention to migrate and non-migration from low/middle-income countries, 1970-2022: a systematic review. BMJ Glob Health. 2023;8(5):e012338. doi: 10.1136/bmjgh-2023-012338 37156560 PMC10174016

[62] Rehm J, Sulkowska U, Mańczuk M, Boffetta P, Powles J, Popova S, et al. Alcohol accounts for a high proportion of premature mortality in central and eastern Europe. Int J Epidemiol. 2007;36(2):458–67. doi: 10.1093/ije/dyl294 17251244

[63] Stefler D, Murphy M, Irdam D, Horvat P, Jarvis M, King L, et al. Smoking and mortality in Eastern Europe: results from the privmort retrospective cohort study of 177 376 individuals. Nicotine Tob Res. 2018;20(6):749–54. doi: 10.1093/ntr/ntx122 28575492

[64] Mahmood T, Bitzer J, Nizard J, Short M. The sexual reproductive health of women: unfinished business in the Eastern Europe and Central Asia region. Eur J Contracept Reprod Health Care. 2020;25(2):87–94. doi: 10.1080/13625187.2020.1718638 32148113

[65] Mabrouk M, Ferrini G, Montanari G, Di Donato N, Raimondo D, Stanghellini V, et al. Does colorectal endometriosis alter intestinal functions? A prospective manometric and questionnaire-based study. Fertil Steril. 2012;97(3):652–6. doi: 10.1016/j.fertnstert.2011.12.019 22260854

[66] Bulutlar E, Uluutku Bulutlar GB, Boz Izceyhan G, Aktaş Ö, Albayrak Denizli AB, Kiliççi Ç. Innovative minimally invasive technique for pelvic organ prolapse: V-Notes lateral suspension. J Obstet Gynaecol Res. 2025;51(2):e16232. doi: 10.1111/jog.16232 39915914

[67] Shek KL, Dietz HP. Assessment of pelvic organ prolapse: a review. Ultrasound Obstet Gynecol. 2016;48(6):681–92. doi: 10.1002/uog.15881 26865209

[68] Levy G, Lindo FM, Lozo S, Prodigalidad L, Brito LGO, Lo T-S, et al. A roadmap for training in urogynecology: IUGA international initiative. Int Urogynecol J. 2024;35(6):1131–5. doi: 10.1007/s00192-024-05789-1 38691126

[69] Meadows AR, Byfield R, Bingham D, Diop H. Strategies to promote maternal health equity: the role of perinatal quality collaboratives. Obstet Gynecol. 2023;142(4):821–30. doi: 10.1097/AOG.0000000000005347 37678899 PMC10510807

[70] Lawson S, Sacks A. Pelvic floor physical therapy and women’s health promotion. J Midwifery Womens Health. 2018;63(4):410–7. doi: 10.1111/jmwh.12736 29778086

[71] Schulten SFM, Claas-Quax MJ, Weemhoff M, van Eijndhoven HW, van Leijsen SA, Vergeldt TF, et al. Risk factors for primary pelvic organ prolapse and prolapse recurrence: an updated systematic review and meta-analysis. Am J Obstet Gynecol. 2022;227(2):192–208. doi: 10.1016/j.ajog.2022.04.046 35500611

[72] GBD 2019 Acute and Chronic Care Collaborators. Characterising acute and chronic care needs: insights from the global burden of disease study 2019. Nat Commun. 2025;16(1):4235. doi: 10.1038/s41467-025-56910-x 40335470 PMC12059133

[73] Chandrakumar DL, Aref-Adib M, Odejinmi F. Advancing women’s health: the imperative for public health screening of uterine fibroids for personalized care. Eur J Obstet Gynecol Reprod Biol. 2024;299:266–71. doi: 10.1016/j.ejogrb.2024.06.014 38917750

[74] Hatch B, Hoopes M, Darney BG, Marino M, Templeton AR, Schmidt T, et al. Impacts of the affordable care act on receipt of women’s preventive services in community health centers in medicaid expansion and nonexpansion states. Womens Health Issues. 2021;31(1):9–16. doi: 10.1016/j.whi.2020.08.011 33023807 PMC9206529

[75] Laily A, Nair I, Shank SE, Wettschurack C, Khamis G, Dykstra C, et al. Enhancing uterine fibroid care: clinician perspectives on diagnosis, disparities, and strategies for improving health care. Womens Health Rep (New Rochelle). 2024;5(1):293–304. doi: 10.1089/whr.2023.0113 38558944 PMC10979696

[76] Mariani G, Kasznia-Brown J, Paez D, Mikhail MN, H Salama D, Bhatla N, et al. Improving women’s health in low-income and middle-income countries. Part II: the needs of diagnostic imaging. Nucl Med Commun. 2017;38(12):1024–8. doi: 10.1097/MNM.0000000000000752 28953209 PMC5704652

[77] Vincent R, Krishnakumar K. School-based interventions for promoting sexual and reproductive health of adolescents in india: a review. J Psychosex Health. 2022;4(2):102–10. doi: 10.1177/26318318221089621

[78] Boutari C, Mantzoros CS. A 2022 update on the epidemiology of obesity and a call to action: as its twin COVID-19 pandemic appears to be receding, the obesity and dysmetabolism pandemic continues to rage on. Metabolism. 2022;133:155217. doi: 10.1016/j.metabol.2022.155217 35584732 PMC9107388

[79] De Corte P, Klinghardt M, von Stockum S, Heinemann K. Time to diagnose endometriosis: current status, challenges and regional characteristics-a systematic literature review. BJOG. 2025;132(2):118–30. doi: 10.1111/1471-0528.17973 39373298 PMC11625652

[80] Gibson-Helm M, Teede H, Dunaif A, Dokras A. Delayed diagnosis and a lack of information associated with dissatisfaction in women with polycystic ovary syndrome. J Clin Endocrinol Metab. 2017;102(2):604–12. doi: 10.1210/jc.2016-2963 27906550 PMC6283441

[81] Jayatilleke K. Challenges in implementing surveillance tools of high-income countries (HICs) in low middle income countries (LMICs). Curr Treat Options Infect Dis. 2020;12(3):191–201. doi: 10.1007/s40506-020-00229-2 32874140 PMC7453076

[82] Halbreich U, Backstrom T, Eriksson E, O’brien S, Calil H, Ceskova E, et al. Clinical diagnostic criteria for premenstrual syndrome and guidelines for their quantification for research studies. Gynecol Endocrinol. 2007;23(3):123–30. doi: 10.1080/09513590601167969 17454164

