# Supplementary material for: Global, regional, and national prevalence and trends of gynecological diseases among women of childbearing age from 1990 to 2021: An analysis of the global burden of disease study 2021
Source: PLoS One. 2025 Aug 1;20(8):e0329336. doi: 10.1371/journal.pone.0329336 (PMC12316229; doi:10.1371/journal.pone.0329336)
Supplement: S1 File — S2 Table. Socio-demographic Index (SDI) quintiles for 204 countries and territories estimated in GBD 2021. S3 Table. Case definitions and mapping of International Classification of Diseases (ICD) codes to gynecological diseases in GBD 2021. S4 Table. The global prevalence of gynecological diseases among women aged 15–49 years in 1990 and 2021, along with the trends and changes observed between these years, by country and territories. S5 Table. The global prevalence of uterine fibroids among women aged 15–49 years in 1990 and 2021, along with the trends and changes observed between these years. S6 Table. The global prevalence of polycystic ovarian syndrome among women aged 15–49 years in 1990 and 2021, along with the trends and changes observed between these years. S7 Table. The global prevalence of female infertility among women aged 15–49 years in 1990 and 2021, along with the trends and changes observed between these years. S8 Table. The global prevalence of endometriosis among women aged 15–49 years in 1990 and 2021, along with the trends and changes observed between these years. S9 Table. The global prevalence of genital prolapses among women aged 15–49 years in 1990 and 2021, along with the trends and changes observed between these years. S10 Table. The global prevalence of premenstrual syndrome among women aged 15–49 years in 1990 and 2021, along with the trends and changes observed between these years. S11 Table. The global prevalence of other gynecological diseases among women aged 15–49 years in 1990 and 2021, along with the trends and changes observed between these years. S12 Table. The global prevalence of uterine fibroids among women aged 15–49 years in 1990 and 2021, along with the trends and changes observed between these years, by country and territories. S13 Table. The global prevalence of polycystic ovarian syndrome among women aged 15–49 years in 1990 and 2021, along with the trends and changes observed between these years, by country and territori [file pone.0329336.s001.pdf]

Supplementary Materials

Table of contents

S1 Table. 21 GBD world regions and 204 countries and territories within each region 3

S2 Table. Socio-demographic Index (SDI) quintiles for 204 countries and territories estimated in GBD 2021 4

S3 Table. Case definitions and mapping of International Classification of Diseases (ICD) codes to gynecological diseases in GBD 2021 4

S4 Table. The global prevalence of gynecological diseases among women aged 15-49 years in 1990 and 2021, along with the trends and changes observed between these years, by country and territories 5

S5 Table. The global prevalence of uterine fibroids among women aged 15-49 years in 1990 and 2021, along with the trends and changes observed between these years..... 10

S6 Table. The global prevalence of polycystic ovarian syndrome among women aged 15-49 years in 1990 and 2021, along with the trends and changes observed between these years..... 11

S7 Table. The global prevalence of female infertility among women aged 15-49 years in 1990 and 2021, along with the trends and changes observed between these years 12

S8 Table. The global prevalence of endometriosis among women aged 15-49 years in 1990 and 2021, along with the trends and changes observed between these years..... 13

S9 Table. The global prevalence of genital prolapse among women aged 15-49 years in 1990 and 2021, along with the trends and changes observed between these years..... 14

S10 Table. The global prevalence of premenstrual syndrome among women aged 15-49 years in 1990 and 2021, along with the trends and changes observed between these years 15

S11 Table. The global prevalence of other gynecological diseases among women aged 15-49 years in 1990 and 2021, along with the trends and changes observed between these years 16

S12 Table. The global prevalence of uterine fibroids among women aged 15-49 years in 1990 and 2021, along with the trends and changes observed between these years, by country and territories 17

S13 Table. The global prevalence of polycystic ovarian syndrome among women aged 15-49 years in 1990 and 2021, along with the trends and changes observed between these years, by country and territories..... 22

S14 Table. The global prevalence of female infertility among women aged 15-49 years in 1990 and 2021, along with the trends and changes observed between these years, by country and territories 27

S15 Table. The global prevalence of endometriosis among women aged 15-49 years in 1990 and 2021, along with the trends and changes observed between these years, by country and territories 32

S16 Table. The global prevalence of genital prolapse among women aged 15-49 years in 1990 and 2021, along with the trends and changes observed between these years, by country and territories

.... 37

S17 Table. The global prevalence of premenstrual syndrome among women aged 15-49 years in 1990 and 2021, along with the trends and changes observed between these years, by country and territories 42

S18 Table. The global prevalence of other gynecological diseases among women aged 15-49 years in 1990 and 2021, along with the trends and changes observed between these years, by country and territories 47

S1 Table. 21 GBD world regions and 204 countries and territories within each region

GBD World Region (n=21)	Countries and territories (n=204)
Central Asia	Armenia, Azerbaijan, Georgia, Kazakhstan, Kyrgyzstan, Mongolia, Tajikistan, Turkmenistan, Uzbekistan
Central Europe	Albania, Bosnia and Herzegovina, Bulgaria, Croatia, Czech Republic, Hungary, Montenegro, North Macedonia, Poland (subnational), Romania, Serbia, Slovakia, Slovenia
Eastern Europe	Belarus, Estonia, Latvia, Lithuania, Moldova, Russia (subnational), Ukraine
Australasia	Australia, New Zealand (subnational Māori + non-Māori)
High-income Asia Pacific	Brunei, Japan (subnational), Singapore, South Korea
High-income North America	Canada, Greenland, United States (subnational)
Southern Latin America	Argentina, Chile, Uruguay
Western Europe	Andorra, Austria, Belgium, Cyprus, Denmark, Finland, France, Germany, Greece, Iceland, Ireland, Israel, Italy (subnational), Luxembourg, Malta, Monaco, Netherlands, Norway (subnational), Portugal, San Marino, Spain, Sweden (subnational), Switzerland, United Kingdom (subnational)
Andean Latin America	Bolivia, Ecuador, Peru
Caribbean	Antigua and Barbuda, Bahamas, Barbados, Belize, Bermuda, Cuba, Dominica, Dominican Republic, Grenada, Guyana, Haiti, Jamaica, Puerto Rico, Saint Kitts and Nevis, Saint Lucia, Saint Vincent and the Grenadines, Suriname, Trinidad and Tobago, US Virgin Islands
Central Latin America	Colombia, Costa Rica, El Salvador, Guatemala, Honduras, Mexico (subnational), Nicaragua, Panama, Venezuela
Tropical Latin America	Brazil (subnational), Paraguay
North Africa and Middle East	Afghanistan, Algeria, Bahrain, Egypt, Iran (subnational), Iraq, Jordan, Kuwait, Lebanon, Libya, Morocco, Oman, Palestine, Qatar, Saudi Arabia, Sudan, Syria, Tunisia, Türkiye, United Arab Emirates, Yemen
South Asia	Bangladesh, Bhutan, India (subnational), Nepal, Pakistan (subnational)
East Asia	China, North Korea, Taiwan (province of China)
Oceania	American Samoa, Cook Islands, Federated States of Micronesia, Fiji, Guam, Kiribati, Marshall Islands, Nauru, Niue, Northern Mariana Islands, Palau, Papua New Guinea, Samoa, Solomon Islands, Tokelau, Tonga, Tuvalu, Vanuatu
Southeast Asia	Cambodia, Indonesia (subnational), Laos, Malaysia, Maldives, Mauritius, Myanmar, Philippines (subnational), Seychelles, Sri Lanka, Thailand, Timor-Leste, Vietnam
Central sub-Saharan Africa	Angola, Central African Republic, Congo (Brazzaville), Democratic Republic of the Congo, Equatorial Guinea, Gabon
Eastern sub-Saharan Africa	Burundi, Comoros, Djibouti, Eritrea, Ethiopia (subnational), Kenya (subnational), Madagascar, Malawi, Mozambique, Rwanda, Somalia, South Sudan, Tanzania, Uganda, Zambia
Southern sub-Saharan Africa	Botswana, eSwatini, Lesotho, Namibia, South Africa (subnational), Zimbabwe
Western sub-Saharan Africa	Benin, Burkina Faso, Cape Verde, Cameroon, Chad, Côte d'Ivoire, Gambia, Ghana, Guinea, Guinea-Bissau, Liberia, Mali, Mauritania, Niger, Nigeria (subnational), São Tomé and Príncipe, Senegal, Sierra Leone, Togo

S2 Supplementary Table. Socio-demographic Index (SDI) quintiles for 204 countries and territories estimated in GBD 2021

**SDI Quintile**	**Locations included based on SDI values in 2021 from GBD 2021 results**
Low SDI	Afghanistan, Angola, Benin, Burkina Faso, Burundi, Central African Republic, Chad, Côte d'Ivoire, Democratic Republic of the Congo, Eritrea, Ethiopia, Gambia, Guinea, Guinea-Bissau, Haiti, Liberia, Madagascar, Malawi, Mali, Mozambique, Nepal, Niger<br>Papua New Guinea, Rwanda, Senegal, Sierra Leone, Solomon Islands, Somalia, South Sudan, Timor-Leste, Togo, Uganda, United Republic of Tanzania, Yemen
Low-middle SDI	Bangladesh, Bhutan, Bolivia (Plurinational State of), Cabo Verde, Cambodia, Cameroon, Comoros, Congo, Democratic People's Republic of Korea, Djibouti, Egypt, El Salvador, Eswatini, Ghana, Guatemala, Honduras, India, Kenya, Kiribati, Kyrgyzstan, Lao People's Democratic Republic, Lesotho, Marshall Islands, Mauritania, Micronesia (Federated States of), Morocco, Myanmar<br>Nicaragua, Nigeria, Pakistan, Samoa, Sao Tome and Principe, Sudan, Tajikistan, Tuvalu, Vanuatu, Venezuela (Bolivarian Republic of), Zambia, Zimbabwe
Middle SDI	Algeria, Belize, Botswana, Brazil, Colombia, Cuba, Dominican Republic, Ecuador, Equatorial Guinea, Fiji, Gabon, Grenada, Guyana, Indonesia, Iraq, Jamaica, Maldives, Mexico, Mongolia, Namibia, Nauru, Palestine, Paraguay, Peru, Philippines, Saint Lucia, Saint Vincent and the Grenadines, South Africa, Suriname, Syrian Arab Republic, Thailand, Tokelau, Tonga, Tunisia, Turkmenistan, Uzbekistan, Vietnam
High-middle SDI	Albania, American Samoa, Antigua and Barbuda, Argentina, Armenia, Azerbaijan, Bahrain, Barbados, Belarus, Bosnia and Herzegovina, Bulgaria, Chile, China, Cook Islands, Costa Rica, Croatia, Dominica, Georgia, Greece, Guam, Hungary, Iran (Islamic Republic of), Jordan, Kazakhstan, Lebanon, Libya, Malaysia, Malta, Mauritius, Montenegro, Niue, North Macedonia, Northern Mariana Islands, Oman, Palau, Panama, Portugal, Republic of Moldova, Romania, Saint Kitts and Nevis, Serbia, Seychelles, Spain, Sri Lanka, Trinidad and Tobago, Turkey, Ukraine, Uruguay
High SDI	Andorra, Australia, Austria, Bahamas, Belgium, Bermuda, Brunei Darussalam, Canada, Cyprus, Czechia, Denmark, Estonia, Finland, France, Georgia, Germany, Greenland, Iceland, Ireland, Israel, Italy, Japan, Kuwait, Latvia, Lithuania, Luxembourg, Monaco, Netherlands, New Zealand, Norway, Poland, Puerto Rico, Qatar, Republic of Korea, Russian Federation, San Marino, Saudi Arabia, Singapore, Slovakia, Slovenia, Sweden, Switzerland, Taiwan (Province of China), United Arab Emirates, United Kingdom, United States of America, United States Virgin Islands

S3 Table. Case definitions and mapping of International Classification of Diseases (ICD) codes to gynecological diseases in GBD 2021

**Gynecological diseases**	**Case definitions**	**ICD10**	**ICD9**
Uterine fibroids	The American College of Obstetricians and Gynecologists (ACOG) definition was used as the reference, identifying cases diagnosed through a pelvic exam followed by or in conjunction with ultrasonography, hysteroscopy, hysterosalpingography, sonohysterography, or laparoscopy.	D25-D26, D28.2	218-219, 219.1-219.9, 236.0
Polycystic ovarian syndrome	The diagnostic criteria used by experts include those from the National Institutes of Health (NIH), the Rotterdam criteria, and the Androgen Excess Society (AES). In GBD 2021, polycystic ovarian syndrome (PCOS) cases were identified based on any of these three diagnostic approaches.	E28.2	256.4
Female infertility	Infertility in GBD 2021 is defined as the absence of a live birth in a couple who have been in a union for at least five years while actively trying to conceive, or in a couple who have not had a live birth in at least five years since their last child, without the use of contraception during this period.	N97-N98.9	628-628.9, V26-V26.49, V26.51, V26.8-V26.9, V59.7-V59.74
Endometriosis	The GBD 2021 framework follows ACOG guidelines, defining cases as those diagnosed through a pelvic exam confirmed by laparoscopy or laparotomy.	N80-N80.9	617-617.9
Genital prolapse	Genital prolapse cases were defined according to ACOG criteria, which state that mild descent of the pelvic organs is not considered pathological unless symptoms such as pressure (with or without a bulge), sexual dysfunction, or disruption of normal lower urinary tract or bowel function are present.	N81-N81.9	618-618.9
Premenstrual syndrome	Premenstrual syndrome was defined based on ACOG guidelines, requiring the presence of at least one emotional or physical symptom occurring within the five days before menses, resolving within four days after the onset of menstruation, and not recurring until at least day 13 of the cycle, in each of the three prior menstrual cycles.	N85.0-N85.1, N92-N93.9, N94.3, N95.0	621.2-621.3, 621.31-621.32, 621.34, 625.4, 626, 626.2-626.9, 627.0-627.1
Other gynecological diseases	Other gynecological diseases include all gynecological disorders that are not related to menstruation or bleeding and do not fall under any of the specified gynecological disease categories in GBD 2021. This category includes breast disorders, inflammatory disease of the cervix uteri, diseases of Bartholin's gland, other inflammatory conditions of the vagina and vulva, vulvovaginal ulceration and inflammation in diseases classified elsewhere, non-inflammatory disorders of the ovary, fallopian tube, and broad ligament, other non-inflammatory disorders of the uterus, cervix, vagina, vulva, and perineum, as well as menopausal and other perimenopausal disorders.	B37.3-B37.49, N61-N64.9, N72-N72.0, N75-N77.8, N83-N84, N84.2-N85, N85.2-N86, N88-N91.5, N94-N94.2, N94.4-N95, N95.1-N95.9, R30-R39.9, R87-R87.9	112.1-112.2, 611-611.9, 616-616.9, 620-621.1, 621.30, 621.33, 621.35-622.0, 622.3-622.6, 622.8-625.3, 625.5-625.9, 626.0-626.1, 627, 627.2-627.9, 629-629.9, 788-788.29, 788.4-788.9, 788.99, 799.81, V07.4-V07.59, V13.2, V13.29, V18.7, V43.82, V45.71, V45.83, V47.5, V49.81, V72.3-V72.31

S4 Table. The global prevalence of gynecological diseases among women aged 15-49 years in 1990 and 2021, along with the trends and changes observed between these years, by country and territories

Countries	Number of cases (Thousands) in 1990	Age-standardized rate per 100,000 population (95% UI) in 2021	Number of cases (Thousands) in 2021	Age-standardized rate per 100,000 population (95% UI) in 2021	Estimated annual percentage changes (95% CI) from 1990 to 2021
Afghanistan	1346.19	69487.43 (69361.92 to 69613.15)	4483.07	69690.04 (69622.1 to 69758.03)	-0.01 (-0.02 to 0)
Albania	451.7	56167.29 (55997.5 to 56337.53)	346.77	56138.96 (55951.57 to 56326.85)	0 (-0.01 to 0.01)
Algeria	3678.08	70050.16 (69974.5 to 70125.89)	8069.66	70291.56 (70242.88 to 70340.27)	0 (-0.01 to 0.01)
American Samoa	6.21	54157.74 (52765.29 to 55582.51)	6.4	55470.83 (54107.36 to 56861.48)	0.08 (0.08 to 0.09)
Andorra	10.1	66764.04 (65456.12 to 68093.17)	13.88	66375.71 (65218.92 to 67550.49)	-0.01 (-0.01 to -0.01)
Angola	1353.15	63618.34 (63507.01 to 63729.83)	4614.6	64526.36 (64465.76 to 64587.01)	0.04 (0.03 to 0.05)
Antigua and Barbuda	9.81	61835.91 (60594.68 to 63099.01)	15.22	61957.23 (60972.06 to 62955.24)	0.01 (0.01 to 0.02)
Argentina	4738.23	59918.84 (59864.79 to 59972.94)	7153.74	59758.11 (59714.29 to 59801.95)	-0.01 (-0.01 to 0)
Armenia	518.26	61002.89 (60831.1 to 61175.11)	461.57	60277.51 (60099.91 to 60455.57)	-0.04 (-0.05 to -0.03)
Australia	2655.82	59232.66 (59161.31 to 59304.07)	3609.6	58741.14 (58680.01 to 58802.32)	-0.03 (-0.03 to -0.02)
Austria	1468.86	73518.17 (73398.73 to 73637.77)	1448.5	71091.89 (70974.39 to 71209.57)	-0.11 (-0.12 to -0.11)
Azerbaijan	1096.01	61193.79 (61071.98 to 61315.82)	1730	61535.87 (61443.18 to 61628.68)	-0.02 (-0.05 to 0)
Bahamas	43.09	61457.24 (60863.69 to 62055.73)	66.73	61792.64 (61324.04 to 62263.98)	0 (-0.01 to 0)
Bahrain	77.56	70100.33 (69570.14 to 70634.38)	231.96	70279.56 (69993.38 to 70566.65)	0 (-0.01 to 0)
Bangladesh	14335.99	62697.97 (62663.54 to 62732.42)	28744.79	63286.7 (63263.46 to 63309.94)	0.03 (0.02 to 0.03)
Barbados	41.8	61700.29 (61103.91 to 62301.54)	44.77	61777.9 (61202.1 to 62358.03)	0.01 (0 to 0.01)
Belarus	1700.38	66646.65 (66545.98 to 66747.44)	1481.5	66350.64 (66239.79 to 66461.66)	-0.03 (-0.03 to -0.02)
Belgium	1669.7	67685.48 (67582.48 to 67788.6)	1728.27	68065.02 (67962.61 to 68167.55)	0.01 (0 to 0.02)
Belize	22.66	58511.78 (57698.32 to 59335.69)	70.87	60261.86 (59814.82 to 60711.57)	0.08 (0.08 to 0.09)
Benin	616.08	61129.88 (60969.74 to 61290.38)	1778.62	59939.57 (59848.09 to 60031.17)	-0.06 (-0.07 to -0.05)
Bermuda	10.77	61603.34 (60433.33 to 62792.59)	8.67	61870.67 (60533.59 to 63232.77)	0.01 (0.01 to 0.02)
Bhutan	81.93	62017.4 (61565.9 to 62471.87)	127.41	62011.71 (61669.25 to 62355.74)	0 (0 to 0.01)
Bolivia (Plurinational State of)	911.84	62744.38 (62612.21 to 62876.79)	1986.62	64581.25 (64491.04 to 64671.56)	0.09 (0.09 to 0.1)
Bosnia and Herzegovina	669.14	57796.87 (57657.9 to 57936.11)	429.84	57108.85 (56934.86 to 57283.28)	-0.03 (-0.04 to -0.03)
Botswana	189.11	64466.98 (64160.32 to 64774.94)	445.97	65788.64 (65594.6 to 65983.15)	0.06 (0.04 to 0.08)
Brazil	23156.86	60977.92 (60952.58 to 61003.27)	35800.37	60039.95 (60020.21 to 60059.7)	-0.1 (-0.11 to -0.08)
Brunei Darussalam	32.99	49974.99 (49412.39 to 50543.62)	64.21	50823.25 (50429.38 to 51219.61)	0.04 (0.04 to 0.05)
Bulgaria	1196.43	56939.9 (56837.27 to 57042.66)	839.24	56673.3 (56546.99 to 56799.84)	-0.04 (-0.04 to -0.03)
Burkina Faso	1162.91	60442.28 (60328.94 to 60555.81)	3039.02	60482.99 (60412.1 to 60553.95)	0 (-0.01 to 0.02)
Burundi	722.36	61392.34 (61243.85 to 61541.15)	1815.05	62155.98 (62060.82 to 62251.28)	0.03 (0.02 to 0.05)
Cabo Verde	44.93	62850.93 (62225.95 to 63481.47)	93.16	62949.19 (62541.76 to 63358.82)	0.01 (0 to 0.02)
Cambodia	1315.87	54582.33 (54485.77 to 54679.03)	2520.42	56113.68 (56043.96 to 56183.48)	0.03 (0.01 to 0.05)
Cameroon	1325.62	61297.21 (61188.25 to 61406.34)	4536.1	62345.51 (62286.13 to 62404.94)	0.03 (-0.01 to 0.06)
Canada	4008.32	53652.99 (53600.11 to 53705.9)	4540.86	53207.89 (53158.45 to 53257.37)	-0.07 (-0.09 to -0.06)
Central African Republic	381.54	64305.83 (64094.02 to 64518.23)	855.5	66099.46 (65955.6 to 66243.59)	0.09 (0.09 to 0.1)
Chad	740.79	60984.4 (60840.55 to 61128.54)	2095.27	60693.62 (60607.18 to 60780.16)	-0.06 (-0.07 to -0.04)
Chile	2111.22	60035.75 (59953.82 to 60117.78)	2863.82	59874.49 (59804.91 to 59944.13)	-0.07 (-0.11 to -0.03)
China	182914	58588.84 (58580.14 to 58597.55)	185222.5	56602.1 (56593.71 to 56610.49)	-0.24 (-0.31 to -0.18)
Colombia	4885.16	58671.26 (58617.49 to 58725.07)	7755.23	59158.73 (59117.03 to 59200.45)	0.03 (0.02 to 0.04)
Comoros	61.37	63235.03 (62713.05 to 63760.71)	124.02	65389.04 (65022.52 to 65757.2)	0.16 (0.13 to 0.18)
Congo	327.83	64029.24 (63798.35 to 64260.86)	898.39	64866.76 (64731.58 to 65002.18)	0.02 (0 to 0.05)
Cook Islands	2.43	55260.25 (53038 to 57558.12)	2.41	56294.27 (54057.79 to 58603.21)	0.07 (0.06 to 0.07)

Costa Rica	447.64	59652.32 (59472.01 to 59833.13)	791.08	60485.25 (60351.7 to 60619.03)	0.04 (0.03 to 0.05)
Côte d'Ivoire	1512.9	61140.21 (61036.61 to 61243.96)	3910.06	62187.27 (62123.9 to 62250.7)	0.05 (0.03 to 0.06)
Croatia	715.79	58832.08 (58695.52 to 58968.88)	539.22	58345.22 (58186.57 to 58504.23)	-0.07 (-0.11 to -0.04)
Cuba	1854.31	62295.24 (62204.22 to 62386.36)	1569.16	61894.59 (61796.37 to 61992.94)	-0.03 (-0.03 to -0.02)
Cyprus	135.89	68607.38 (68242.74 to 68973.53)	256.82	68621.47 (68346.38 to 68897.6)	-0.05 (-0.1 to 0)
Czechia	1498.04	57356.05 (57263.26 to 57448.96)	1359.63	57013.5 (56912.94 to 57114.21)	0.04 (0.01 to 0.07)
Democratic People's Republic of Korea	3072.26	55809.07 (55745.85 to 55872.34)	3782.4	56886.62 (56829.1 to 56944.18)	0.07 (0.07 to 0.07)
Democratic Republic of the Congo	4946.34	62929.18 (62871.37 to 62987.04)	12655.84	63856.3 (63819.92 to 63892.69)	0.03 (0 to 0.06)
Denmark	872.29	65939.48 (65800.41 to 66078.79)	840.93	65568.36 (65427.1 to 65709.88)	0 (-0.02 to 0.01)
Djibouti	58.42	64485.17 (63935.93 to 65038.61)	209.5	65220.89 (64940.55 to 65502.21)	0.05 (0.02 to 0.08)
Dominica	9.74	60569.67 (59336.2 to 61824.07)	10.05	61614.92 (60415.43 to 62832.71)	0.06 (0.05 to 0.07)
Dominican Republic	1050.81	59465.26 (59346.08 to 59584.65)	1737.87	60689.3 (60598.81 to 60779.9)	0.06 (0.04 to 0.08)
Ecuador	1540.51	64443.19 (64337.85 to 64548.68)	3046.98	65162.65 (65089.28 to 65236.08)	0.04 (0.02 to 0.06)
Egypt	8492.79	69139.67 (69092.3 to 69187.07)	17582.14	69677.41 (69644.61 to 69710.22)	0.01 (0 to 0.01)
El Salvador	719.03	58219.61 (58079.61 to 58359.89)	1048.62	59528.61 (59414.15 to 59643.24)	0.1 (0.09 to 0.11)
Equatorial Guinea	58.41	63830.53 (63298.16 to 64366.64)	222.79	64779.79 (64503.03 to 65057.59)	0.05 (0.04 to 0.06)
Eritrea	463.67	62904.5 (62716.79 to 63092.68)	1027.99	64801.13 (64673.58 to 64928.9)	0.11 (0.1 to 0.12)
Estonia	256.62	66222.04 (65965.3 to 66479.58)	191.09	65891.84 (65589.13 to 66195.74)	-0.04 (-0.05 to -0.03)
Eswatini	112.32	63689.89 (63297.58 to 64084.33)	195.08	64689.05 (64394.71 to 64984.59)	0.05 (0.03 to 0.06)
Ethiopia	6622.09	62994.93 (62944.65 to 63045.25)	16586.17	63775.85 (63743.78 to 63807.93)	0.08 (0.06 to 0.09)
Fiji	106.99	56470.12 (56126.04 to 56815.97)	128.15	56340.5 (56032.05 to 56650.28)	0 (-0.01 to 0)
Finland	844.15	64948.99 (64808.7 to 65089.54)	756.12	65103.5 (64955.74 to 65251.54)	-0.01 (-0.08 to 0.06)
France	9763.85	67083.99 (67041.75 to 67126.26)	9665.25	67005.39 (66962.78 to 67048.02)	0.01 (0 to 0.01)
Gabon	131.17	65092.38 (64718.11 to 65468.6)	306.02	65794.53 (65557.19 to 66032.58)	0.05 (0.02 to 0.07)
Gambia	126.88	61934.8 (61568.51 to 62303.13)	351.17	62038.23 (61823.93 to 62253.2)	-0.01 (-0.03 to 0)
Georgia	836.95	61226.46 (61094.55 to 61358.59)	502.55	61596.66 (61423.53 to 61770.2)	0.01 (0 to 0.01)
Germany	13545.66	68294.22 (68257.53 to 68330.92)	11925.52	67828.31 (67789.28 to 67867.36)	-0.02 (-0.03 to -0.02)
Ghana	1998.48	61356.39 (61267.75 to 61445.14)	5416.11	61934.49 (61881.45 to 61987.56)	0.01 (0 to 0.02)
Greece	1687.76	66836.17 (66735.3 to 66937.15)	1494.39	66460.25 (66349.77 to 66570.88)	-0.02 (-0.02 to -0.01)
Greenland	7.64	52801.16 (51587.12 to 54038.47)	6.69	52540.61 (51283.3 to 53822.22)	-0.01 (-0.02 to -0.01)
Grenada	11.23	61011.32 (59841.51 to 62201.13)	15.69	61757.09 (60788.91 to 62737.48)	0.05 (0.05 to 0.06)
Guam	18.76	54729.78 (53931.48 to 55538.74)	19.89	55338.46 (54569.65 to 56115.72)	0.03 (0.02 to 0.04)
Guatemala	978.92	56729.29 (56612.36 to 56846.43)	2540.92	59540.62 (59466.2 to 59615.12)	0.13 (0.11 to 0.16)
Guinea	798.67	61964.31 (61825.49 to 62103.39)	1900.94	61735.3 (61644.24 to 61826.48)	0.02 (0.01 to 0.03)
Guinea-Bissau	131.11	61394.51 (61048.95 to 61741.77)	303.94	61755.66 (61528.57 to 61983.5)	0.03 (0.02 to 0.04)
Guyana	118.17	61409.31 (61044.14 to 61776.42)	123.99	62084.35 (61735.98 to 62434.28)	0.03 (0 to 0.06)
Haiti	866.35	59123.24 (58994.81 to 59251.92)	2096.04	60108.88 (60026.89 to 60190.96)	0.06 (0.05 to 0.06)
Honduras	572.84	57962.05 (57804.98 to 58119.48)	1659.03	60101.48 (60008.54 to 60194.54)	0.14 (0.12 to 0.15)
Hungary	1461.84	56689.06 (56596.35 to 56781.89)	1255.18	56692.07 (56589.03 to 56795.27)	-0.02 (-0.03 to -0.01)
Iceland	42.06	65273.55 (64648.08 to 65903.92)	52.77	65267.23 (64709.49 to 65828.82)	-0.02 (-0.04 to 0)
India	126272.4	64798.64 (64787.13 to 64810.16)	247704	66072.05 (66063.79 to 66080.31)	0.07 (0.04 to 0.09)
Indonesia	27523.91	59198.27 (59175.52 to 59221.02)	44906.25	59404.87 (59387.48 to 59422.27)	-0.02 (-0.04 to 0)
Iran (Islamic Republic of)	8278.67	71868.97 (71817.53 to 71920.44)	17719.74	72483.08 (72448.66 to 72517.51)	-0.01 (-0.03 to 0.01)
Iraq	2520.37	69033.73 (68944.39 to 69123.18)	7089.51	70149.91 (70097.79 to 70202.05)	0.04 (0.03 to 0.04)
Ireland	576.04	66354.76 (66182.81 to 66527.06)	790.18	66133.2 (65984.35 to 66282.34)	-0.01 (-0.01 to 0)
Israel	787.64	65652.97 (65506.56 to 65799.67)	1451.55	65298.96 (65192.6 to 65405.46)	-0.01 (-0.01 to -0.01)

Italy	10305.6	72000.65 (71956.61 to 72044.71)	8246.76	66614.04 (66566.96 to 66661.16)	-0.3 (-0.33 to -0.27)
Jamaica	346.99	61734.3 (61519.43 to 61949.84)	481.3	62442.53 (62265.64 to 62619.8)	0.04 (0.04 to 0.05)
Japan	19372.67	59839.48 (59812.45 to 59866.52)	14963.61	59222.83 (59191.79 to 59253.89)	-0.08 (-0.11 to -0.06)
Jordan	506.93	69041.73 (68838.7 to 69245.28)	2073.33	69925.75 (69829.77 to 70021.84)	0.02 (0.01 to 0.03)
Kazakhstan	2431.01	60344.4 (60267.09 to 60421.8)	2912.82	60310.83 (60241.11 to 60380.61)	-0.01 (-0.02 to 0)
Kenya	2907.79	62230.51 (62153.4 to 62307.71)	8082.86	64506.44 (64460.47 to 64552.43)	0.1 (0.08 to 0.12)
Kiribati	9.8	54536 (53427.03 to 55664.94)	17.48	55551.93 (54724.35 to 56389.89)	0.06 (0.06 to 0.07)
Kuwait	280.22	70090.39 (69816.65 to 70365.16)	1110.87	70162.89 (70026.51 to 70299.53)	0 (-0.01 to 0)
Kyrgyzstan	601.65	60457.95 (60296.66 to 60619.64)	1046.07	61185.8 (61067.99 to 61303.8)	0.03 (0.01 to 0.04)
Lao People's Democratic Republic	500.35	54136.08 (53980.96 to 54291.58)	1094.31	55918.02 (55812.32 to 56023.89)	0.13 (0.12 to 0.14)
Latvia	429.81	65345.77 (65149.97 to 65542.04)	268.75	65979.7 (65722.54 to 66237.73)	0.02 (-0.02 to 0.06)
Lebanon	505.68	70533.36 (70336.38 to 70730.79)	1091.43	70988.2 (70853.5 to 71123.12)	0.05 (0.04 to 0.05)
Lesotho	234.07	65460.35 (65191.02 to 65730.59)	317.39	66163.21 (65926.89 to 66400.27)	-0.01 (-0.04 to 0.01)
Liberia	311.56	61347.49 (61119.54 to 61576.21)	821.12	62539.23 (62401.51 to 62677.19)	0.03 (0 to 0.05)
Libya	553.06	69230.35 (69035.63 to 69425.55)	1425.11	70580.64 (70464.55 to 70696.88)	0.05 (0.05 to 0.06)
Lithuania	612.33	66156.46 (65990.63 to 66322.61)	394.98	66688.96 (66477.43 to 66901.07)	-0.04 (-0.09 to 0.01)
Luxembourg	67.84	68217.74 (67700.92 to 68737.98)	109.67	67968.08 (67558.71 to 68379.65)	-0.04 (-0.05 to -0.02)
Madagascar	1532.92	61922.15 (61818.97 to 62025.5)	4356.89	64098.04 (64035.94 to 64160.2)	0.11 (0.09 to 0.13)
Malawi	1279.22	61876.47 (61763.21 to 61989.91)	2858.2	62041.44 (61965.7 to 62117.27)	0.03 (0.01 to 0.06)
Malaysia	2441.48	55856.3 (55784.45 to 55928.25)	4799.13	56998.69 (56947.51 to 57049.92)	0.07 (0.06 to 0.08)
Maldives	24.85	56040.87 (55292.54 to 56797.97)	68.15	57805.9 (57366.51 to 58248.07)	0.02 (-0.02 to 0.06)
Mali	1073.55	60449.32 (60331.49 to 60567.35)	2950.58	60255.52 (60183.11 to 60328.01)	0.01 (0 to 0.02)
Malta	65.32	67605.98 (67083.88 to 68131.36)	66.09	67490.71 (66965.78 to 68019.29)	0 (-0.01 to 0.02)
Marshall Islands	5	54595.29 (52992.86 to 56244.8)	8.12	55628.08 (54419.08 to 56858.34)	0.06 (0.06 to 0.06)
Mauritania	262.91	60852.03 (60610.14 to 61094.77)	619.69	62368.15 (62208.22 to 62528.43)	0.09 (0.08 to 0.09)
Mauritius	166.59	56396.02 (56120.76 to 56672.46)	182.34	57097.33 (56834.07 to 57361.56)	0.04 (0.03 to 0.04)
Mexico	13202.27	63762.31 (63726.46 to 63798.18)	22510.61	64226.65 (64200.11 to 64253.2)	0.02 (-0.01 to 0.05)
Micronesia (Federated States of)	11.95	54577.9 (53552.63 to 55621.11)	14.17	55778.59 (54853.3 to 56716.13)	0.07 (0.07 to 0.07)
Monaco	4.83	66515.5 (64595.29 to 68488.59)	4.87	66092.8 (64198.86 to 68032.37)	-0.01 (-0.01 to -0.01)
Mongolia	283.35	59764.42 (59529.25 to 60000.4)	516.78	60143.59 (59978.98 to 60308.56)	-0.02 (-0.05 to 0)
Montenegro	88.21	56817.16 (56441.94 to 57194.37)	83.05	56442.32 (56054.84 to 56831.96)	-0.02 (-0.03 to -0.01)
Morocco	4075.77	69848.83 (69778.91 to 69918.8)	6860.76	70855.05 (70802.03 to 70908.11)	0.06 (0.05 to 0.07)
Mozambique	1869.26	63224.05 (63131.05 to 63317.17)	4481.46	64129.93 (64067.65 to 64192.26)	0.01 (-0.03 to 0.04)
Myanmar	5670.22	56324.16 (56276.05 to 56372.31)	8356.47	55653.58 (55615.78 to 55691.39)	-0.08 (-0.1 to -0.06)
Namibia	198.67	64352.15 (64054.36 to 64651.14)	415.43	65208.75 (65007.91 to 65410.09)	0.02 (0 to 0.04)
Nauru	1.28	54336.54 (51325.6 to 57499.43)	1.53	55172.43 (52403.42 to 58061.95)	0.05 (0.04 to 0.05)
Nepal	2720.9	62838.21 (62761.41 to 62915.1)	5493.41	61940.18 (61887.78 to 61992.62)	-0.02 (-0.05 to 0.01)
Netherlands	2671.52	66784.77 (66704.45 to 66865.17)	2468.84	66252.19 (66169.06 to 66335.41)	-0.02 (-0.02 to -0.01)
New Zealand	611.82	67878.5 (67708.18 to 68049.17)	781.55	64729.41 (64585.42 to 64873.65)	-0.13 (-0.17 to -0.09)
Nicaragua	486.1	57906.68 (57734.06 to 58079.77)	1064.86	59401.68 (59288.39 to 59515.15)	0.1 (0.09 to 0.11)
Niger	957.54	60953.96 (60826.58 to 61081.57)	2840.84	60853.4 (60778.16 to 60928.73)	0 (-0.03 to 0.02)
Nigeria	11263.4	61844.19 (61805.75 to 61882.66)	32535.61	61887.87 (61865.76 to 61910)	-0.02 (-0.06 to 0.02)
Niue	0.27	55436.44 (48968.46 to 62544.33)	0.21	55891.02 (48626.97 to 63974.01)	0.02 (0.02 to 0.03)
North Macedonia	288.87	57002.93 (56794.99 to 57211.46)	312.24	57107.59 (56903.75 to 57312.07)	0.01 (0.01 to 0.01)
Northern Mariana Islands	7.51	55456.14 (54151.36 to 56790.79)	6.36	55833.35 (54431.53 to 57263.96)	0.03 (0.03 to 0.04)
Norway	709.65	66759.97 (66604.44 to 66915.79)	820.44	66478.22 (66333.45 to 66623.26)	-0.16 (-0.27 to -0.05)

Oman	221.86	70253.11 (69949.84 to 70557.53)	732.37	70202.32 (70038.62 to 70366.35)	-0.01 (-0.01 to -0.01)
Pakistan	14644.62	65507.46 (65472.84 to 65542.08)	39455.1	66637.48 (66616.4 to 66658.58)	0 (-0.05 to 0.05)
Palau	2.24	55810.47 (53488.26 to 58217.44)	2.13	56071.97 (53635.48 to 58598.98)	0.01 (0 to 0.01)
Palestine	271.03	69296.03 (69019.86 to 69573.13)	860.66	70384.57 (70232.34 to 70537.07)	0.05 (0.05 to 0.06)
Panama	354.68	60375.69 (60171.86 to 60580.11)	639.86	60223.74 (60076.02 to 60371.75)	-0.01 (-0.02 to 0)
Papua New Guinea	492.37	53624.18 (53468.94 to 53779.81)	1385.11	53966.25 (53875.52 to 54057.11)	0.02 (0.01 to 0.03)
Paraguay	514.67	57332.78 (57171.67 to 57494.28)	1095.7	58454.57 (58344.51 to 58564.8)	0.05 (0.04 to 0.07)
Peru	3260.39	63347.72 (63276.66 to 63418.85)	6237.54	64711.74 (64660.88 to 64762.62)	0.06 (0.05 to 0.06)
Philippines	8955.55	59831.52 (59790.98 to 59872.09)	17903.92	61941.77 (61912.87 to 61970.67)	0.28 (0.22 to 0.34)
Poland	5590.96	58418.22 (58369.24 to 58467.23)	5257.63	57818.79 (57767.51 to 57870.1)	-0.07 (-0.12 to -0.03)
Portugal	1624.96	64603.07 (64503.68 to 64702.57)	1503.27	62433.52 (62330.14 to 62537.04)	-0.14 (-0.16 to -0.12)
Puerto Rico	580.85	61198.83 (61041.3 to 61356.67)	471.02	61871.02 (61693.08 to 62049.36)	0.03 (0.03 to 0.04)
Qatar	54.29	68728.56 (68124.27 to 69338.02)	412.63	69264.72 (69041.46 to 69488.66)	0.02 (0.01 to 0.02)
Republic of Korea	6143.45	49430.76 (49391.04 to 49470.5)	5884.32	49353.28 (49312.06 to 49394.52)	-0.12 (-0.34 to 0.11)
Republic of Moldova	754.85	66702.44 (66550.89 to 66854.27)	620.15	66735.24 (66563.01 to 66907.87)	0.01 (0 to 0.02)
Romania	3191.97	56939.95 (56877.16 to 57002.79)	2350.06	56126.11 (56052.09 to 56200.22)	0 (-0.05 to 0.04)
Russian Federation	25740.39	68387.99 (68361.29 to 68414.69)	24262.76	68243.36 (68215.13 to 68271.6)	-0.03 (-0.04 to -0.02)
Rwanda	912.72	60677.48 (60545.69 to 60809.53)	2109.91	62759.47 (62672.87 to 62846.16)	0.12 (0.11 to 0.13)
Saint Kitts and Nevis	5.79	61150.4 (59481.14 to 62864.42)	9.82	61964.62 (60741.11 to 63207.75)	0.04 (0.04 to 0.04)
Saint Lucia	19.6	61139.55 (60246.24 to 62044.44)	28.84	62058.46 (61339.75 to 62783.99)	0.05 (0.05 to 0.06)
Saint Vincent and the Grenadines	15.09	61415.03 (60379.48 to 62466.5)	17.25	61767.27 (60846.76 to 62698.67)	0.01 (0 to 0.01)
Samoa	19.13	55362.91 (54540.52 to 56195.89)	25.83	54959.81 (54281.01 to 55645.44)	-0.04 (-0.05 to -0.03)
San Marino	4.14	66728.45 (64704.43 to 68801.08)	4.85	66291.62 (64376.69 to 68252.27)	-0.01 (-0.01 to -0.01)
Sao Tome and Principe	14.02	61142.45 (60077.26 to 62223.76)	32.73	61833.82 (61154.91 to 62518.93)	0.05 (0.03 to 0.07)
Saudi Arabia	2056.11	69351.88 (69252.38 to 69451.51)	7402.87	70080.79 (70029.72 to 70131.9)	0.03 (0.03 to 0.04)
Senegal	945.35	60758.31 (60629.9 to 60886.96)	2251.24	61786.33 (61703.4 to 61869.36)	0.06 (0.05 to 0.07)
Serbia	1319.6	56313.83 (56217.74 to 56410.05)	1165.67	56102.65 (55999.51 to 56205.94)	0.02 (-0.01 to 0.04)
Seychelles	9.73	56091.41 (54926.16 to 57277.9)	13.72	56083.81 (55144.46 to 57035.56)	-0.01 (-0.03 to 0)
Sierra Leone	580.43	62298.9 (62130.29 to 62467.93)	1310.64	62574.71 (62462.82 to 62686.77)	0 (-0.02 to 0.02)
Singapore	476.2	50888.48 (50742.75 to 51034.54)	778.36	51122.15 (50999.82 to 51244.78)	0 (0 to 0.01)
Slovakia	801.01	60031.05 (59899.32 to 60163.01)	787.1	59706.83 (59570.31 to 59843.64)	-0.1 (-0.15 to -0.05)
Slovenia	287.49	57339.96 (57130.2 to 57550.32)	250.58	57204.82 (56972.62 to 57437.84)	0.02 (-0.04 to 0.08)
Solomon Islands	36.87	53411.82 (52834.72 to 53994.4)	91.25	54771.52 (54412.91 to 55132.03)	0.09 (0.08 to 0.09)
Somalia	984.46	61423.54 (61299.4 to 61547.89)	2791.21	62304.79 (62228.82 to 62380.84)	0.05 (0.04 to 0.05)
South Africa	6089.05	67098.91 (67043.82 to 67154.04)	10314.86	66302.23 (66261.63 to 66342.84)	-0.01 (-0.03 to 0.01)
South Sudan	721.36	61191.19 (61040.89 to 61341.84)	1330.02	61464.87 (61357.75 to 61572.14)	0.02 (0.01 to 0.02)
Spain	6374.19	66799.63 (66747.67 to 66851.63)	6870.34	66652.71 (66600.54 to 66704.92)	0.01 (-0.01 to 0.02)
Sri Lanka	2512.17	55615.71 (55546.09 to 55685.41)	3190.29	56264.05 (56202.18 to 56325.98)	0 (-0.02 to 0.01)
Sudan	2983.95	69959.66 (69877.26 to 70042.13)	7588.08	71148.76 (71097.02 to 71200.54)	0.06 (0.05 to 0.07)
Suriname	56.75	61196.89 (60681.77 to 61715.55)	89.26	61444.9 (61042.31 to 61849.52)	0.01 (0 to 0.01)
Sweden	1324.56	63370.86 (63262.05 to 63479.82)	1390.41	62117.56 (62013.39 to 62221.88)	-0.15 (-0.21 to -0.08)
Switzerland	1221.15	67881.62 (67760.4 to 68003.03)	1367.01	67405.53 (67290.29 to 67520.94)	-0.03 (-0.05 to -0.01)
Syrian Arab Republic	1700.63	69467.76 (69357.23 to 69578.45)	2607.46	70665.16 (70574.45 to 70755.99)	0.06 (0.05 to 0.06)
Taiwan (Province of China)	3184.28	58311.72 (58246.55 to 58376.96)	3674.14	62936.61 (62870.01 to 63003.28)	0.3 (0.23 to 0.37)
Tajikistan	663.67	59717.28 (59559.96 to 59874.99)	1499.45	60226.03 (60128.43 to 60323.76)	0.02 (0.01 to 0.03)
Thailand	8759.2	55873.47 (55835.8 to 55911.16)	9438.95	56743.84 (56706.83 to 56780.87)	0 (-0.02 to 0.03)

Timor-Leste	95.11	52819.58 (52473.98 to 53167.16)	180.9	54712.06 (54448.53 to 54976.65)	0.11 (0.09 to 0.14)
Togo	476.28	61020.28 (60838.94 to 61202.09)	1285.66	62074.1 (61965.54 to 62182.81)	0.03 (0.02 to 0.04)
Tokelau	0.19	55739.52 (47964.53 to 64523.44)	0.18	55852.65 (47927.64 to 64758.14)	0.01 (0 to 0.01)
Tonga	11.13	52832.18 (51818.02 to 53862.41)	13.47	54767.27 (53837.39 to 55709.78)	0.12 (0.11 to 0.13)
Trinidad and Tobago	183.3	61091.56 (60807.98 to 61376.26)	216.47	62046.01 (61782.08 to 62310.86)	0.01 (-0.01 to 0.02)
Tunisia	1337.06	70406.53 (70282.99 to 70530.25)	2253.54	70890.63 (70797.36 to 70983.99)	0.02 (0.02 to 0.03)
Turkey	9567.61	71930.55 (71883.87 to 71977.26)	15825.11	71747.88 (71712.42 to 71783.35)	-0.04 (-0.05 to -0.03)
Turkmenistan	495.58	59821.1 (59642.19 to 60000.49)	747.2	60125.03 (59988.51 to 60261.78)	0.01 (0 to 0.02)
Tuvalu	1.28	53235.15 (50349.32 to 56251.32)	1.56	55051.68 (52322.11 to 57892.95)	0.11 (0.1 to 0.12)
Uganda	2100.02	60958.26 (60869.6 to 61047.03)	5897.87	61771.58 (61718.91 to 61824.3)	0.05 (0.04 to 0.06)
Ukraine	8743.7	68184.33 (68139.02 to 68229.65)	7309.15	68745.7 (68693.68 to 68797.75)	0.03 (0.03 to 0.04)
United Arab Emirates	230.12	69213.17 (68906.9 to 69520.84)	1321.74	69483.63 (69349.14 to 69618.36)	0.02 (0 to 0.04)
United Kingdom	9960.49	69467.95 (69424.63 to 69511.28)	10314.65	66090.41 (66049.77 to 66131.06)	-0.15 (-0.17 to -0.12)
United Republic of Tanzania	3387.47	62165 (62094.71 to 62235.36)	8716.43	62614.06 (62571.19 to 62656.95)	0.01 (0 to 0.01)
United States of America	35800.78	52737.5 (52720.15 to 52754.86)	43572.09	56856.44 (56839.52 to 56873.36)	0.23 (0.15 to 0.31)
United States Virgin Islands	17.49	60548.27 (59650.15 to 61456.94)	10.78	61343.47 (60174.51 to 62530.89)	0.05 (0.05 to 0.06)
Uruguay	448.08	60178.86 (60002.65 to 60355.46)	503.32	59763.31 (59597.86 to 59929.13)	-0.01 (-0.02 to 0)
Uzbekistan	2695.03	59143.13 (59066.54 to 59219.81)	5373.06	59873.04 (59822.26 to 59923.85)	0.06 (0.04 to 0.08)
Vanuatu	17.7	53298.34 (52483.32 to 54124.53)	41.53	54608.6 (54077.52 to 55143.93)	0.09 (0.08 to 0.09)
Venezuela (Bolivarian Republic of)	2751.18	59232 (59159.71 to 59304.38)	4148.23	59590.91 (59533.17 to 59648.7)	0.01 (0 to 0.02)
Vietnam	9082.28	55506.31 (55467.99 to 55544.65)	14546.15	56046.82 (56017.8 to 56075.86)	0.04 (0.04 to 0.05)
Yemen	1759.7	70045.18 (69937.04 to 70153.48)	5612.81	71350.27 (71289.85 to 71410.74)	0.05 (0.04 to 0.06)
Zambia	1006.69	61396.98 (61268.29 to 61525.9)	2876.17	62786.31 (62710.39 to 62862.32)	0.04 (0.01 to 0.07)
Zimbabwe	1384.16	64044.1 (63930.5 to 64157.88)	2486.87	64882.48 (64800.02 to 64965.03)	0.01 (0 to 0.02)

S5 Table. The global prevalence of uterine fibroids among women aged 15-49 years in 1990 and 2021, along with the trends and changes observed between these years

Characteristics	1990		2021		1990−2021	
	Number of cases (Millions)	Age-standardized rate per 100,000 population (95% UI)	Number of cases (Millions)	Age-standardized rate per 100,000 population (95% UI)	Percentage change in absolute number (%)	Estimated annual percentage changes (95% CI)
**Socio-demographic index**						
High	11.35	4903.17 (4900.31 to 4906.03)	13.22	4855.42 (4852.78 to 4858.05)	16.48	-0.02 (-0.18 to 0.15)
High-middle	12.06	4788.54 (4785.81 to 4791.27)	15.64	4436.86 (4434.64 to 4439.08)	29.68	-0.35 (-0.41 to -0.28)
Middle	12.71	3451.67 (3449.74 to 3453.61)	26.3	4067.12 (4065.56 to 4068.67)	106.92	0.49 (0.46 to 0.53)
Low-middle	8.53	3818.79 (3816.19 to 3821.39)	20.87	4508.49 (4506.55 to 4510.43)	144.67	0.66 (0.6 to 0.72)
Low	3.39	3808.33 (3804.2 to 3812.47)	9.08	4100.43 (4097.72 to 4103.14)	167.85	0.3 (0.27 to 0.33)
**Age group (years)**						
15 to 19	0.82	319.5 (193.62 to 485.77)	0.96	317.01 (192.08 to 480.79)	17.07	-0.14 (-0.21 to -0.06)
20 to 24	1.78	730.85 (422.22 to 1096.7)	2.19	745.75 (430.61 to 1112.6)	23.03	-0.08 (-0.15 to 0)
25 to 29	4.1	1862.34 (1198.61 to 2812.98)	5.65	1940.07 (1257.45 to 2936.43)	37.8	0.1 (0.03 to 0.16)
30 to 34	8.1	4259.99 (2548.07 to 6765.28)	13.38	4474.59 (2672.15 to 7072.28)	65.19	0.22 (0.17 to 0.28)
35 to 39	11.83	6822.38 (4470.56 to 10202.37)	20.57	7405.32 (4879.63 to 11001.31)	73.88	0.29 (0.27 to 0.31)
40 to 44	12.17	8681.65 (5763.6 to 12062.53)	22.94	9248.46 (6261.38 to 12705.26)	88.5	0.17 (0.12 to 0.22)
45 to 49	9.28	8151.55 (5757.66 to 10768.99)	19.49	8270.78 (5911.63 to 10918.81)	110.02	0 (-0.06 to 0.07)
**GBD regions**						
High-income Asia Pacific	2.72	5881.5 (5874.49 to 5888.53)	2.56	6018.12 (6010.56 to 6025.7)	-5.88	0.08 (0 to 0.15)
Central Asia	0.89	6784.83 (6770.22 to 6799.48)	1.75	7167.08 (7156.44 to 7177.73)	96.63	0.2 (0.19 to 0.22)
East Asia	6.44	2252.8 (2251.03 to 2254.57)	9.28	2407.71 (2406.14 to 2409.27)	44.1	0.11 (-0.03 to 0.25)
South Asia	8.83	4149.72 (4146.96 to 4152.5)	23.32	5091.57 (5089.5 to 5093.65)	164.1	0.87 (0.76 to 0.98)
Southeast Asia	2.47	2426.7 (2423.6 to 2429.8)	4.78	2583.22 (2580.9 to 2585.54)	93.52	0.17 (0.14 to 0.21)
Australasia	0.08	1448.04 (1437.85 to 1458.29)	0.11	1442.06 (1433.67 to 1450.5)	37.5	-0.06 (-0.07 to -0.04)
Caribbean	0.5	6350.38 (6332.57 to 6368.24)	0.77	6460.19 (6445.75 to 6474.64)	54	0.02 (-0.01 to 0.05)
Central Europe	1.31	4193.83 (4186.64 to 4201.03)	1.24	4042.63 (4035.35 to 4049.91)	-5.34	-0.15 (-0.27 to -0.03)
Eastern Europe	5.68	10150.42 (10142.01 to 10158.82)	6.02	10268.73 (10260.39 to 10277.07)	5.99	0.07 (0.05 to 0.08)
Western Europe	5.99	6178.8 (6173.85 to 6183.76)	6.48	6059.42 (6054.71 to 6064.13)	8.18	-0.02 (-0.1 to 0.06)
Andean Latin America	0.69	9118.73 (9096.91 to 9140.58)	1.57	9309.69 (9295.12 to 9324.28)	127.54	0.04 (0.02 to 0.06)
Central Latin America	2.46	7651.98 (7642.24 to 7661.72)	5	7418.4 (7411.89 to 7424.91)	103.25	-0.13 (-0.16 to -0.11)
Southern Latin America	0.53	4626.13 (4613.72 to 4638.57)	0.89	4955.63 (4945.33 to 4965.94)	67.92	0.12 (0 to 0.24)
Tropical Latin America	1.12	3175.08 (3169.08 to 3181.08)	3.01	4676.94 (4671.65 to 4682.23)	168.75	1.21 (1.12 to 1.31)
North Africa and Middle East	1.29	2058.54 (2054.93 to 2062.16)	3.39	2142.02 (2139.74 to 2144.31)	162.79	0 (-0.06 to 0.05)
High-income North America	2.97	3883.11 (3878.68 to 3887.54)	4.16	4658.1 (4653.62 to 4662.59)	40.07	0.56 (0.24 to 0.88)
Oceania	0.03	2269.44 (2242.77 to 2296.36)	0.08	2437.2 (2419.95 to 2454.55)	166.67	0.18 (0.14 to 0.21)
Central Sub-Saharan Africa	0.42	4411.85 (4398.28 to 4425.46)	1.23	4656.2 (4647.83 to 4664.58)	192.86	0.17 (0.14 to 0.2)
Eastern Sub-Saharan Africa	1.14	3452.68 (3446.14 to 3459.23)	3.03	3554.43 (3550.33 to 3558.52)	165.79	0.13 (0.12 to 0.14)
Southern Sub-Saharan Africa	1	9539.11 (9520.05 to 9558.2)	2	9606.59 (9593.21 to 9619.98)	100	0.04 (0.01 to 0.07)
Western Sub-Saharan Africa	1.51	4535.45 (4528 to 4542.91)	4.51	4738.02 (4733.56 to 4742.48)	198.68	0.11 (0.07 to 0.15)

S6 Table. The global prevalence of polycystic ovarian syndrome among women aged 15-49 years in 1990 and 2021, along with the trends and changes observed between these years

Characteristics	1990		2021		1990−2021	
	Number of cases (Millions)	Age-standardized rate per 100,000 population (95% UI)	Number of cases (Millions)	Age-standardized rate per 100,000 population (95% UI)	Percentage change in absolute number (%)	Estimated annual percentage changes (95% CI)
**Socio-demographic index**						
High	13.17	5771.32 (5768.2 to 5774.45)	16.7	6831.77 (6828.46 to 6835.09)	26.8	0.1 (-0.07 to 0.26)
High-middle	6.69	2411.85 (2410 to 2413.69)	10.55	3464.51 (3462.38 to 3466.65)	57.7	1.2 (1.16 to 1.25)
Middle	10	2240.9 (2239.47 to 2242.33)	23.24	3757.42 (3755.89 to 3758.96)	132.4	1.73 (1.69 to 1.78)
Low-middle	3.86	1427.88 (1426.41 to 1429.34)	11.5	2275.82 (2274.49 to 2277.14)	197.93	1.63 (1.59 to 1.68)
Low	1.06	960.67 (958.77 to 962.57)	3.73	1371.29 (1369.85 to 1372.73)	251.89	1.23 (1.2 to 1.25)
**Age group (years)**						
15 to 19	4.92	1925.13 (1305.23 to 2746.17)	7.66	2523.76 (1707 to 3613.35)	55.69	0.83 (0.81 to 0.86)
20 to 24	6.35	2600.45 (1849.19 to 3620.8)	10.15	3453.97 (2489.13 to 4742.61)	59.84	0.84 (0.8 to 0.88)
25 to 29	6.03	2740.3 (1962.1 to 3836.74)	10.42	3581.86 (2571.9 to 4968.04)	72.8	0.81 (0.76 to 0.86)
30 to 34	5.37	2824.88 (2025.68 to 3934.06)	10.76	3600.5 (2579.79 to 5007.28)	100.37	0.73 (0.7 to 0.76)
35 to 39	4.93	2840.34 (2026.55 to 3941.19)	10.13	3644.97 (2588.05 to 5067.78)	105.48	0.73 (0.69 to 0.78)
40 to 44	4.26	3041.51 (2196.24 to 4233.57)	9.15	3687.03 (2637.99 to 5121.84)	114.79	0.64 (0.58 to 0.7)
45 to 49	2.95	2587.93 (1859.21 to 3573.47)	7.5	3182.5 (2297.85 to 4407.06)	154.24	0.53 (0.46 to 0.59)
**GBD regions**						
High-income Asia Pacific	4.2	9169.77 (9160.97 to 9178.57)	3.89	10140.46 (10130.07 to 10150.87)	-7.38	0.27 (0.22 to 0.31)
Central Asia	0.11	666.42 (662.37 to 670.5)	0.23	923.73 (919.91 to 927.56)	109.09	1.18 (1.11 to 1.25)
East Asia	5.39	1628.2 (1626.79 to 1629.61)	9.87	2970.62 (2968.72 to 2972.53)	83.12	2.03 (1.88 to 2.18)
South Asia	3.11	1230.87 (1229.47 to 1232.27)	10.75	2178 (2176.69 to 2179.31)	245.66	2.14 (2.02 to 2.26)
Southeast Asia	3.51	2931.94 (2928.78 to 2935.1)	10	5448.41 (5445.03 to 5451.79)	184.9	2.3 (2.19 to 2.4)
Australasia	0.43	7890.33 (7866.59 to 7914.13)	0.67	9162.54 (9140.36 to 9184.78)	55.81	0.28 (0.19 to 0.36)
Caribbean	0.21	2256.64 (2246.81 to 2266.52)	0.34	2823.31 (2813.82 to 2832.82)	61.9	0.76 (0.71 to 0.82)
Central Europe	0.11	353.67 (351.57 to 355.78)	0.11	436.36 (433.74 to 439)	0	0.64 (0.59 to 0.69)
Eastern Europe	0.22	396.25 (394.59 to 397.91)	0.25	509.25 (507.18 to 511.33)	13.64	0.98 (0.94 to 1.03)
Western Europe	6.46	6731.9 (6726.7 to 6737.11)	7.01	7501.21 (7495.57 to 7506.85)	8.51	0.21 (0.15 to 0.28)
Andean Latin America	0.43	4571.16 (4557.11 to 4585.26)	1.11	6302.4 (6290.64 to 6314.19)	158.14	1.08 (1.01 to 1.16)
Central Latin America	2.13	5064.02 (5056.96 to 5071.1)	3.81	5576.54 (5570.93 to 5582.14)	78.87	-0.09 (-0.25 to 0.07)
Southern Latin America	0.28	2284.05 (2275.6 to 2292.53)	0.64	3641.84 (3632.9 to 3650.8)	128.57	1.46 (1.25 to 1.68)
Tropical Latin America	0.42	1049.77 (1046.53 to 1053.02)	0.69	1140.95 (1138.26 to 1143.65)	64.29	-0.17 (-0.34 to 0)
North Africa and Middle East	2.31	2962.11 (2958.15 to 2966.08)	6.34	3968.4 (3965.31 to 3971.5)	174.46	1.09 (1.02 to 1.16)
High-income North America	4.29	5699.25 (5693.83 to 5704.68)	6.07	7207.04 (7201.3 to 7212.79)	41.49	-0.5 (-0.99 to -0.01)
Oceania	0.04	2446.54 (2421.15 to 2472.17)	0.12	3384.71 (3365.25 to 3404.26)	200	0.81 (0.65 to 0.98)
Central Sub-Saharan Africa	0.1	855.85 (850.43 to 861.3)	0.42	1292.54 (1288.5 to 1296.6)	320	1.27 (1.13 to 1.42)
Eastern Sub-Saharan Africa	0.42	991.96 (988.8 to 995.12)	1.36	1283.66 (1281.42 to 1285.9)	223.81	0.86 (0.83 to 0.89)
Southern Sub-Saharan Africa	0.22	1668.26 (1661.02 to 1675.53)	0.46	2096.15 (2090.06 to 2102.26)	109.09	0.78 (0.71 to 0.86)
Western Sub-Saharan Africa	0.41	964.11 (961.02 to 967.21)	1.64	1386.77 (1384.56 to 1388.97)	300	0.92 (0.75 to 1.1)

S7 Table. The global prevalence of female infertility among women aged 15-49 years in 1990 and 2021, along with the trends and changes observed between these years

Characteristics	1990		2021		1990−2021	
	Number of cases (Millions)	Age-standardized rate per 100,000 population (95% UI)	Number of cases (Millions)	Age-standardized rate per 100,000 population (95% UI)	Percentage change in absolute number (%)	Estimated annual percentage changes (95% CI)
**Socio-demographic index**						
High	5.09	2149.2 (2147.33 to 2151.07)	7.48	2901.9 (2899.81 to 2903.99)	46.95	1.43 (1.28 to 1.58)
High-middle	16.55	5932.64 (5929.78 to 5935.51)	21.2	6512.44 (6509.65 to 6515.24)	28.1	0.26 (0.24 to 0.29)
Middle	22.58	5453.65 (5451.37 to 5455.93)	39.04	6184.54 (6182.59 to 6186.48)	72.9	0.57 (0.46 to 0.69)
Low-middle	10.74	4162.82 (4160.29 to 4165.35)	30.05	5968.31 (5966.17 to 5970.45)	179.8	1.23 (0.68 to 1.79)
Low	4.69	4516.84 (4512.66 to 4521.03)	12.25	4745.44 (4742.74 to 4748.14)	161.19	0.1 (-0.29 to 0.49)
**Age group (years)**						
15 to 19	0.79	309.8 (38.35 to 915.12)	1.01	334.26 (22.73 to 1077.24)	27.85	-0.13 (-0.48 to 0.23)
20 to 24	7.96	3259.37 (1172.96 to 6555.44)	13.08	4453.62 (1571.49 to 8990.96)	64.32	1.07 (0.78 to 1.36)
25 to 29	10.62	4824.44 (1523.3 to 11815.87)	19.17	6588.05 (2106.54 to 15873.6)	80.51	1.2 (0.91 to 1.49)
30 to 34	13.28	6985.46 (1803.91 to 15953.4)	26.87	8987.52 (2319.04 to 21434.92)	102.33	0.84 (0.63 to 1.06)
35 to 39	17.09	9852.72 (2973.16 to 22109.47)	30.6	11014.8 (3056.8 to 24959.98)	79.05	0.43 (0.28 to 0.59)
40 to 44	9.83	7013.4 (1853.54 to 15899.33)	19.07	7687.07 (1920.07 to 17631.66)	94	0.28 (0.2 to 0.36)
45 to 49	0.12	103.77 (20.68 to 545.57)	0.28	120.84 (20.47 to 715.91)	133.33	-0.22 (-0.61 to 0.17)
**GBD regions**						
High-income Asia Pacific	0.66	1348.06 (1344.78 to 1351.34)	0.56	1249.13 (1245.81 to 1252.46)	-15.15	-0.39 (-0.65 to -0.13)
Central Asia	0.52	2956.2 (2947.96 to 2964.46)	0.8	3132.55 (3125.67 to 3139.45)	53.85	0.9 (0.64 to 1.16)
East Asia	25.46	8085.58 (8082.4 to 8088.75)	30.1	8420.38 (8417.34 to 8423.42)	18.22	0.01 (-0.04 to 0.05)
South Asia	11.11	4501.67 (4498.98 to 4504.35)	35.56	7134.49 (7132.13 to 7136.84)	220.07	1.95 (1.2 to 2.72)
Southeast Asia	5.04	4468.67 (4464.7 to 4472.64)	11	5976.74 (5973.21 to 5980.28)	118.25	1.68 (1.35 to 2.02)
Australasia	0.01	269.82 (265.51 to 274.18)	0.02	312.78 (308.82 to 316.78)	100	0.86 (0.71 to 1.01)
Caribbean	0.43	4863.96 (4849.22 to 4878.74)	0.59	4914.71 (4902.16 to 4927.27)	37.21	-0.11 (-0.28 to 0.06)
Central Europe	1.31	4179.04 (4171.87 to 4186.22)	1.33	5154.89 (5146.03 to 5163.78)	1.53	0.89 (0.75 to 1.03)
Eastern Europe	4.01	6745.87 (6739.24 to 6752.5)	3.64	7324.22 (7316.46 to 7331.99)	-9.23	0.61 (0.49 to 0.73)
Western Europe	1.73	1759.75 (1757.12 to 1762.38)	2.49	2514.3 (2511.15 to 2517.44)	43.93	1.48 (1.2 to 1.76)
Andean Latin America	0.01	141.54 (138.93 to 144.18)	0.18	1034.67 (1029.9 to 1039.45)	1700	8.21 (6.69 to 9.76)
Central Latin America	0.62	1708.35 (1704.03 to 1712.68)	2.27	3352.29 (3347.93 to 3356.66)	266.13	1.18 (0.79 to 1.57)
Southern Latin America	0.28	2355.09 (2346.4 to 2363.81)	0.39	2173.19 (2166.34 to 2180.05)	39.29	-0.24 (-0.33 to -0.16)
Tropical Latin America	1.04	2736.37 (2731.06 to 2741.68)	2.22	3475.04 (3470.46 to 3479.63)	113.46	1.71 (1.14 to 2.29)
North Africa and Middle East	1.93	2608.97 (2605.19 to 2612.76)	6.36	3920.11 (3917.06 to 3923.16)	229.53	1.19 (0.89 to 1.49)
High-income North America	0.84	1049.2 (1046.95 to 1051.44)	1.48	1733.01 (1730.22 to 1735.81)	76.19	3.01 (1.6 to 4.44)
Oceania	0.05	3394.59 (3363.84 to 3425.59)	0.08	2169.51 (2153.95 to 2185.17)	60	-1.6 (-1.88 to -1.31)
Central Sub-Saharan Africa	0.59	5467.94 (5453.66 to 5482.26)	1.71	5861.7 (5852.77 to 5870.64)	189.83	-0.17 (-0.78 to 0.44)
Eastern Sub-Saharan Africa	1.96	5071.96 (5064.62 to 5079.3)	3.96	4007.53 (4003.51 to 4011.56)	102.04	-1.26 (-1.53 to -0.99)
Southern Sub-Saharan Africa	0.69	5576.66 (5563.27 to 5590.08)	0.78	3511.71 (3503.92 to 3519.51)	13.04	-0.74 (-1.48 to 0)
Western Sub-Saharan Africa	1.41	3501.37 (3495.36 to 3507.4)	4.58	4192.43 (4188.51 to 4196.35)	224.82	-0.32 (-0.6 to -0.03)

S8 Table. The global prevalence of endometriosis among women aged 15-49 years in 1990 and 2021, along with the trends and changes observed between these years

Characteristics	1990		2021		1990−2021	
	Number of cases (Millions)	Age-standardized rate per 100,000 population (95% UI)	Number of cases (Millions)	Age-standardized rate per 100,000 population (95% UI)	Percentage change in absolute number (%)	Estimated annual percentage changes (95% CI)
**Socio-demographic index**						
High	2.64	1145.73 (1144.35 to 1147.11)	2.31	918.77 (917.58 to 919.97)	-12.5	-0.86 (-0.95 to -0.76)
High-middle	3.45	1253.16 (1251.82 to 1254.49)	3.15	992.47 (991.35 to 993.58)	-8.7	-0.73 (-0.82 to -0.63)
Middle	5.68	1333.48 (1332.35 to 1334.61)	6.12	976.79 (976.01 to 977.56)	7.75	-1.05 (-1.13 to -0.97)
Low-middle	5.05	1945.61 (1943.87 to 1947.35)	5.84	1169.2 (1168.24 to 1170.15)	15.64	-1.67 (-1.71 to -1.64)
Low	2.25	2135.81 (2132.93 to 2138.69)	3.6	1387.51 (1386.04 to 1388.98)	60	-1.4 (-1.47 to -1.32)
**Age group (years)**						
15 to 19	0.81	316.37 (194.57 to 508.31)	0.76	249.69 (155.46 to 400.02)	-6.17	-0.75 (-0.8 to -0.7)
20 to 24	3.73	1529.52 (866.52 to 2390.03)	3.48	1183.94 (677.2 to 1837.59)	-6.7	-0.9 (-0.97 to -0.83)
25 to 29	4.26	1935.49 (1202.44 to 2991.5)	4.23	1453.61 (916.52 to 2199.94)	-0.7	-0.96 (-1.01 to -0.91)
30 to 34	3.33	1749.18 (1048.36 to 2726.87)	3.74	1252.35 (768.87 to 1939.41)	12.31	-1.07 (-1.11 to -1.03)
35 to 39	2.89	1663.87 (1003.33 to 2528.38)	3.3	1186.9 (736.97 to 1778.08)	14.19	-1.12 (-1.18 to -1.05)
40 to 44	2.38	1698.79 (985.12 to 2578.23)	3.02	1217.01 (708.03 to 1821.81)	26.89	-1.13 (-1.2 to -1.06)
45 to 49	1.69	1482.77 (971.47 to 2141.71)	2.52	1069.35 (697.56 to 1536.77)	49.11	-1.08 (-1.14 to -1.01)
**GBD regions**						
High-income Asia Pacific	0.66	1438.92 (1435.44 to 1442.42)	0.49	1226.72 (1223.18 to 1230.26)	-25.76	-0.64 (-0.76 to -0.52)
Central Asia	0.23	1355.2 (1349.43 to 1360.99)	0.27	1070.62 (1066.54 to 1074.71)	17.39	-0.44 (-0.62 to -0.26)
East Asia	3.83	1214.05 (1212.81 to 1215.3)	2.78	784.43 (783.49 to 785.38)	-27.42	-1.55 (-1.73 to -1.37)
South Asia	4.77	1953.43 (1951.65 to 1955.22)	5.64	1148.31 (1147.36 to 1149.26)	18.24	-1.77 (-1.8 to -1.74)
Southeast Asia	1.86	1613.14 (1610.76 to 1615.52)	2.28	1236.76 (1235.15 to 1238.37)	22.58	-0.8 (-0.83 to -0.77)
Australasia	0.06	1201.83 (1192.59 to 1211.12)	0.08	1046.03 (1038.57 to 1053.53)	33.33	-0.28 (-0.36 to -0.2)
Caribbean	0.11	1161.65 (1154.52 to 1168.83)	0.11	870.89 (865.63 to 876.17)	0	-0.87 (-0.9 to -0.83)
Central Europe	0.31	1024.5 (1020.91 to 1028.11)	0.24	935.52 (931.71 to 939.34)	-22.58	-0.22 (-0.37 to -0.07)
Eastern Europe	0.97	1709.67 (1706.25 to 1713.1)	0.83	1674.73 (1670.99 to 1678.48)	-14.43	0.33 (0.15 to 0.5)
Western Europe	0.91	942.48 (940.55 to 944.42)	0.84	898.45 (896.51 to 900.4)	-7.69	-0.08 (-0.1 to -0.05)
Andean Latin America	0.11	1225.88 (1218.47 to 1233.33)	0.15	837.14 (832.86 to 841.44)	36.36	-1.15 (-1.22 to -1.07)
Central Latin America	0.48	1199.96 (1196.46 to 1203.47)	0.54	795.93 (793.81 to 798.05)	12.5	-1.27 (-1.36 to -1.18)
Southern Latin America	0.12	998.54 (992.9 to 1004.2)	0.15	836.27 (832.02 to 840.54)	25	-0.42 (-0.55 to -0.3)
Tropical Latin America	0.45	1169.82 (1166.35 to 1173.29)	0.59	951.74 (949.3 to 954.19)	31.11	-1.09 (-1.33 to -0.84)
North Africa and Middle East	1.46	1949.61 (1946.35 to 1952.88)	2.11	1325.7 (1323.91 to 1327.49)	44.52	-1.3 (-1.36 to -1.24)
High-income North America	0.76	985.79 (983.57 to 988.01)	0.55	641.79 (640.09 to 643.49)	-27.63	-1.93 (-2.09 to -1.76)
Oceania	0.03	2317.4 (2292.02 to 2343.03)	0.07	1921.6 (1906.74 to 1936.56)	133.33	-0.59 (-0.61 to -0.57)
Central Sub-Saharan Africa	0.24	2041.69 (2033.21 to 2050.2)	0.39	1272.66 (1268.58 to 1276.75)	62.5	-1.44 (-1.57 to -1.3)
Eastern Sub-Saharan Africa	0.72	1773.79 (1769.5 to 1778.09)	1.12	1109.07 (1106.94 to 1111.19)	55.56	-1.51 (-1.56 to -1.45)
Southern Sub-Saharan Africa	0.19	1471.03 (1464.15 to 1477.95)	0.25	1146.5 (1141.99 to 1151.03)	31.58	-0.75 (-0.79 to -0.72)
Western Sub-Saharan Africa	0.81	1957.77 (1953.3 to 1962.25)	1.58	1389.15 (1386.91 to 1391.39)	95.06	-1.05 (-1.11 to -0.99)

S9 Table. The global prevalence of genital prolapses among women aged 15-49 years in 1990 and 2021, along with the trends and changes observed between these years

Characteristics	1990		2021		1990−2021	
	Number of cases (Millions)	Age-standardized rate per 100,000 population (95% UI)	Number of cases (Millions)	Age-standardized rate per 100,000 population (95% UI)	Percentage change in absolute number (%)	Estimated annual percentage changes (95% CI)
**Socio-demographic index**						
High	2.53	1120.02 (1118.64 to 1121.4)	2.88	1009.91 (1008.74 to 1011.08)	13.83	-0.77 (-1.04 to -0.49)
High-middle	2.26	971.61 (970.33 to 972.89)	3.19	853.89 (852.95 to 854.84)	41.15	-0.26 (-0.44 to -0.07)
Middle	6.08	1771.59 (1770.16 to 1773.02)	9.08	1387.78 (1386.87 to 1388.68)	49.34	-1.01 (-1.1 to -0.93)
Low-middle	8.98	4214.15 (4211.37 to 4216.94)	11.49	2533.01 (2531.54 to 2534.48)	27.95	-1.88 (-1.99 to -1.77)
Low	3.12	3773.46 (3769.21 to 3777.71)	5.36	2582.52 (2580.31 to 2584.74)	71.79	-1.36 (-1.49 to -1.24)
**Age group (years)**						
15 to 19	0.05	18.96 (7.12 to 34.73)	0.06	20.05 (7.68 to 36.9)	20	0.07 (0.02 to 0.12)
20 to 24	0.51	207.28 (120.62 to 328.92)	0.59	200.38 (111.14 to 330.79)	15.69	-0.6 (-0.81 to -0.39)
25 to 29	1.53	696.87 (475.23 to 1014.99)	1.72	592.44 (396.63 to 868.12)	12.42	-1.15 (-1.38 to -0.92)
30 to 34	2.8	1472.79 (1005.63 to 2081.03)	3.37	1127.37 (763.04 to 1618.49)	20.36	-1.28 (-1.44 to -1.13)
35 to 39	4.47	2578.08 (1803.8 to 3548.3)	5.75	2069.69 (1430.5 to 2881.3)	28.64	-1 (-1.15 to -0.85)
40 to 44	5.97	4258.88 (2824.64 to 5960.75)	8.39	3382.6 (2244.67 to 4768.36)	40.54	-0.92 (-1.03 to -0.82)
45 to 49	7.66	6727.45 (4800.79 to 8974.39)	12.13	5149.6 (3631.7 to 6856.31)	58.36	-0.86 (-0.93 to -0.8)
**GBD regions**						
High-income Asia Pacific	0.14	294.2 (292.68 to 295.74)	0.12	239.36 (238 to 240.73)	-14.29	-0.69 (-0.76 to -0.62)
Central Asia	0.05	514.63 (510.23 to 519.06)	0.1	435.32 (432.64 to 438.01)	100	0 (-0.26 to 0.26)
East Asia	1.66	662.04 (661.01 to 663.06)	2.09	495.04 (494.36 to 495.72)	25.9	-0.85 (-1.03 to -0.66)
South Asia	10.44	5089.79 (5086.67 to 5092.91)	12.9	2852.97 (2851.41 to 2854.53)	23.56	-2.21 (-2.37 to -2.06)
Southeast Asia	0.65	734.6 (732.78 to 736.42)	1.11	592.21 (591.1 to 593.31)	70.77	-0.67 (-0.7 to -0.64)
Australasia	0.04	746.84 (739.34 to 754.41)	0.06	738.34 (732.45 to 744.27)	50	0.17 (0.04 to 0.31)
Caribbean	0.25	3260.55 (3247.61 to 3273.54)	0.3	2487.63 (2478.68 to 2496.6)	20	-0.8 (-0.83 to -0.76)
Central Europe	0.18	610.05 (607.22 to 612.88)	0.21	598.48 (595.93 to 601.05)	16.67	0.31 (0.01 to 0.61)
Eastern Europe	0.37	717.76 (715.42 to 720.1)	0.41	663.83 (661.8 to 665.88)	10.81	0.68 (0.28 to 1.09)
Western Europe	1.87	1923.59 (1920.83 to 1926.35)	2.17	1949.23 (1946.62 to 1951.85)	16.04	0.02 (-0.06 to 0.1)
Andean Latin America	0.27	3830.32 (3815.74 to 3844.94)	0.43	2612.4 (2604.61 to 2620.22)	59.26	-1.26 (-1.35 to -1.17)
Central Latin America	0.58	1946.17 (1941.08 to 1951.27)	1	1493.5 (1490.58 to 1496.43)	72.41	-1.54 (-1.81 to -1.27)
Southern Latin America	0.19	1686.07 (1678.47 to 1693.69)	0.26	1428.73 (1423.23 to 1434.24)	36.84	-0.56 (-0.62 to -0.49)
Tropical Latin America	2.05	5962.69 (5954.42 to 5970.97)	2.31	3614.03 (3609.37 to 3618.71)	12.68	-2.03 (-2.17 to -1.88)
North Africa and Middle East	1.65	3022.44 (3017.78 to 3027.1)	3.77	2462.95 (2460.46 to 2465.44)	128.48	-0.5 (-0.67 to -0.33)
High-income North America	0.68	951.92 (949.66 to 954.19)	0.6	651.9 (650.25 to 653.55)	-11.76	-2.65 (-3.52 to -1.77)
Oceania	0.01	827.51 (810.41 to 844.89)	0.02	754 (744.05 to 764.04)	100	-0.29 (-0.31 to -0.28)
Central Sub-Saharan Africa	0.18	2044.3 (2034.63 to 2054.02)	0.38	1597.29 (1592.18 to 1602.42)	111.11	-0.7 (-0.79 to -0.62)
Eastern Sub-Saharan Africa	0.66	2246.85 (2241.31 to 2252.4)	1.41	1820.41 (1817.35 to 1823.48)	113.64	-0.55 (-0.63 to -0.47)
Southern Sub-Saharan Africa	0.16	1708.84 (1700.37 to 1717.33)	0.27	1385.8 (1380.57 to 1391.05)	68.75	-0.61 (-0.63 to -0.59)
Western Sub-Saharan Africa	0.91	3033.83 (3027.46 to 3040.22)	2.08	2374.21 (2370.94 to 2377.49)	128.57	-0.72 (-0.8 to -0.64)

S10 Table. The global prevalence of premenstrual syndrome among women aged 15-49 years in 1990 and 2021, along with the trends and changes observed between these years

Characteristics	1990		2021		1990−2021	
	Number of cases (Millions)	Age-standardized rate per 100,000 population (95% UI)	Number of cases (Millions)	Age-standardized rate per 100,000 population (95% UI)	Percentage change in absolute number (%)	Estimated annual percentage changes (95% CI)
**Socio-demographic index**						
High	95.53	41884.86 (41876.44 to 41893.27)	106.34	43005.52 (42997.25 to 43013.8)	11.32	0.1 (0.06 to 0.14)
High-middle	126.2	45694.15 (45686.11 to 45702.2)	138.52	44717.78 (44710.17 to 44725.39)	9.76	-0.14 (-0.18 to -0.1)
Middle	202.13	46223.21 (46216.65 to 46229.78)	289.63	46622.64 (46617.26 to 46628.02)	43.29	-0.02 (-0.05 to 0)
Low-middle	123.82	46739.26 (46730.79 to 46747.74)	238.67	47575.08 (47569 to 47581.15)	92.76	0.05 (0.04 to 0.05)
Low	45.56	42436.09 (42423.29 to 42448.9)	116.11	43722.81 (43714.59 to 43731.03)	154.85	0.1 (0.09 to 0.12)
**Age group (years)**						
15 to 19	92	36003.9 (24869.82 to 51177.01)	108.27	35655.03 (24649.41 to 50937.59)	17.68	-0.05 (-0.08 to -0.02)
20 to 24	101.91	41741.65 (28135.8 to 55351.22)	130.07	44278.21 (29763.31 to 59071.39)	27.63	0.13 (0.09 to 0.16)
25 to 29	92.06	41824.96 (29333.08 to 55196.38)	126.23	43378.73 (30274.85 to 57592.2)	37.12	0.06 (0.04 to 0.08)
30 to 34	85.67	45061.11 (29322.83 to 61458.4)	135.04	45174.75 (29390.93 to 62147.29)	57.63	-0.01 (-0.03 to 0)
35 to 39	94.93	54728.44 (39709.16 to 70028.56)	152.95	55055.96 (39816.1 to 70559.99)	61.12	0.01 (-0.01 to 0.03)
40 to 44	80.11	57126.46 (41103.39 to 71654.34)	142.16	57301.62 (41205.34 to 71932.3)	77.46	-0.01 (-0.03 to 0.01)
45 to 49	47.14	41420.54 (28994.96 to 56093.06)	95.26	40426.27 (28444.83 to 55113.88)	102.08	-0.11 (-0.12 to -0.09)
**GBD regions**						
High-income Asia Pacific	19.82	43141.01 (43121.96 to 43160.08)	16.32	42311.81 (42290.58 to 42333.05)	-17.66	-0.13 (-0.23 to -0.03)
Central Asia	7.23	44397.04 (44363.29 to 44430.82)	10.93	44831.09 (44804.43 to 44857.76)	51.18	0 (-0.02 to 0.02)
East Asia	145.86	44377.74 (44370.35 to 44385.12)	145.63	43618.34 (43611.05 to 43625.64)	-0.16	-0.18 (-0.25 to -0.11)
South Asia	122.91	49341.32 (49332.39 to 49350.24)	247.93	50420.87 (50414.57 to 50427.18)	101.72	0.06 (0.06 to 0.07)
Southeast Asia	56.62	48169.86 (48156.92 to 48182.8)	87.59	47685.32 (47675.33 to 47695.31)	54.7	-0.07 (-0.08 to -0.06)
Australasia	2.36	43780.67 (43724.71 to 43836.68)	3.1	42511.02 (42463.33 to 42558.75)	31.36	-0.07 (-0.08 to -0.06)
Caribbean	4.14	45377.27 (45332.65 to 45421.93)	5.49	45682.51 (45644.28 to 45720.76)	32.61	0.02 (0.01 to 0.02)
Central Europe	14.68	47318.44 (47294.17 to 47342.73)	12.37	47096.37 (47069.31 to 47123.43)	-15.74	-0.03 (-0.04 to -0.02)
Eastern Europe	27.57	49201.76 (49183.28 to 49220.25)	24.1	48960.64 (48940.27 to 48981.01)	-12.59	-0.07 (-0.09 to -0.04)
Western Europe	42.89	44654.49 (44641.1 to 44667.88)	40.89	43286.97 (43273.5 to 43300.45)	-4.66	-0.08 (-0.09 to -0.07)
Andean Latin America	4.05	43775.84 (43731.74 to 43819.97)	7.9	45238.11 (45206.5 to 45269.73)	95.06	0.1 (0.09 to 0.11)
Central Latin America	18.72	45826.03 (45804.48 to 45847.6)	32.1	47058.28 (47041.99 to 47074.57)	71.47	0.09 (0.08 to 0.1)
Southern Latin America	5.3	43236.54 (43199.61 to 43273.49)	7.47	42581.93 (42551.35 to 42612.52)	40.94	-0.03 (-0.04 to -0.02)
Tropical Latin America	19.09	48421.92 (48399.76 to 48444.09)	29.58	48299.69 (48282.23 to 48317.17)	54.95	0 (-0.02 to 0.03)
North Africa and Middle East	30.17	40746.06 (40730.98 to 40761.15)	67.06	42020.28 (42010.21 to 42030.35)	122.27	0.05 (0.03 to 0.07)
High-income North America	28.11	37552.45 (37538.49 to 37566.41)	36.53	42965.45 (42951.48 to 42979.43)	29.95	0.53 (0.45 to 0.62)
Oceania	0.63	41622.32 (41515.59 to 41729.28)	1.44	42033.81 (41964.63 to 42103.08)	128.57	0.05 (0.04 to 0.06)
Central Sub-Saharan Africa	4.73	40030.94 (39993.18 to 40068.73)	13.14	41617.84 (41594.58 to 41641.12)	177.8	0.11 (0.07 to 0.15)
Eastern Sub-Saharan Africa	17.2	41698.16 (41677.29 to 41719.03)	45.83	44193.62 (44180.29 to 44206.96)	166.45	0.19 (0.18 to 0.2)
Southern Sub-Saharan Africa	5.75	44630.42 (44592.45 to 44668.41)	9.76	45007.55 (44979.2 to 45035.9)	69.74	0.01 (0 to 0.02)
Western Sub-Saharan Africa	15.98	38668.67 (38648.64 to 38688.71)	44.83	38988.1 (38976.23 to 38999.98)	180.54	0.03 (-0.02 to 0.08)

S11 Table. The global prevalence of other gynecological diseases among women aged 15-49 years in 1990 and 2021, along with the trends and changes observed between these years

Characteristics	1990		2021		1990−2021	
	Number of cases (Millions)	Age-standardized rate per 100,000 population (95% UI)	Number of cases (Millions)	Age-standardized rate per 100,000 population (95% UI)	Percentage change in absolute number (%)	Estimated annual percentage changes (95% CI)
**Socio-demographic index**						
High	48.33	20839.56 (20833.67 to 20845.44)	50.68	19692.56 (19687.1 to 19698.02)	4.86	-0.27 (-0.34 to -0.19)
High-middle	58.17	22069.3 (22063.57 to 22075.02)	64.49	19263.26 (19258.5 to 19268.02)	10.86	-0.66 (-0.77 to -0.55)
Middle	72.6	18582.8 (18578.43 to 18587.17)	112.93	17687.33 (17684.06 to 17690.6)	55.55	-0.31 (-0.41 to -0.22)
Low-middle	53.14	22416.31 (22410.18 to 22422.45)	106.18	22156.7 (22152.47 to 22160.94)	99.81	-0.08 (-0.1 to -0.06)
Low	26.27	27934.27 (27923.34 to 27945.2)	66.14	28333.19 (28326.23 to 28340.15)	151.77	0.04 (0.03 to 0.05)
**Age group (years)**						
15 to 19	3.35	1312.26 (807.2 to 2039.05)	4.76	1566.28 (1001.05 to 2326.7)	42.09	0.9 (0.69 to 1.12)
20 to 24	21.28	8716.76 (6019.13 to 12195.16)	27.96	9518.44 (6715.82 to 13180.49)	31.39	0.32 (0.28 to 0.36)
25 to 29	46.17	20978.8 (14763.68 to 28899.41)	61.71	21205.87 (15143.73 to 29139.17)	33.66	-0.03 (-0.06 to 0.01)
30 to 34	54.35	28589.38 (21668.53 to 37003.74)	82.74	27678.98 (21207.46 to 35402.2)	52.24	-0.16 (-0.19 to -0.13)
35 to 39	54.13	31207.69 (22707.97 to 40463.64)	84.16	30293.72 (22452.16 to 38693.12)	55.48	-0.22 (-0.31 to -0.13)
40 to 44	45.2	32232.04 (22800.92 to 43293.38)	76.03	30645.93 (22156.08 to 40449.47)	68.21	-0.39 (-0.51 to -0.26)
45 to 49	34.22	30070.23 (21850.88 to 38401.37)	63.33	26876.95 (20087.05 to 33991.41)	85.07	-0.56 (-0.66 to -0.46)
**GBD regions**						
High-income Asia Pacific	4.48	9843.7 (9834.55 to 9852.85)	3.19	8074.24 (8065.14 to 8083.35)	-28.79	-0.9 (-1.1 to -0.7)
Central Asia	3.18	21417.48 (21392.89 to 21442.1)	5.18	20795.97 (20778.03 to 20813.93)	62.89	-0.11 (-0.13 to -0.09)
East Asia	49.72	16622.99 (16618.28 to 16627.71)	43.94	12107.87 (12104.22 to 12111.53)	-11.63	-1.46 (-1.73 to -1.19)
South Asia	47.35	21008.77 (21002.69 to 21014.84)	94.76	19971.6 (19967.56 to 19975.63)	100.13	-0.21 (-0.25 to -0.18)
Southeast Asia	9.48	8865.75 (8859.97 to 8871.54)	14.71	7965.72 (7961.65 to 7969.79)	55.17	-0.47 (-0.55 to -0.39)
Australasia	1.23	22658.65 (22618.5 to 22698.85)	1.61	21184.75 (21151.93 to 21217.62)	30.89	-0.2 (-0.24 to -0.17)
Caribbean	1.42	17243.55 (17214.83 to 17272.31)	2.04	17030.64 (17007.27 to 17054.03)	43.66	-0.05 (-0.05 to -0.04)
Central Europe	3.72	11938.28 (11926.11 to 11950.46)	2.95	10329.22 (10317.15 to 10341.3)	-20.7	-0.46 (-0.66 to -0.27)
Eastern Europe	15.76	27615.14 (27601.42 to 27628.87)	15.03	26927.25 (26913.32 to 26941.19)	-4.63	-0.12 (-0.14 to -0.1)
Western Europe	33.41	34411.38 (34399.7 to 34423.06)	30.19	30797.22 (30786.1 to 30808.35)	-9.64	-0.41 (-0.44 to -0.37)
Andean Latin America	2.05	24652.2 (24617.68 to 24686.76)	4.16	24095.84 (24072.65 to 24119.06)	102.93	-0.08 (-0.09 to -0.06)
Central Latin America	6.23	17377.02 (17362.98 to 17391.07)	10.37	15305.9 (15296.58 to 15315.22)	66.45	-0.16 (-0.31 to 0)
Southern Latin America	2.73	23049.73 (23022.34 to 23077.15)	4	22463.64 (22441.63 to 22485.67)	46.52	-0.2 (-0.24 to -0.16)
Tropical Latin America	5.36	14562.14 (14549.6 to 14574.69)	8.06	12843.78 (12834.9 to 12852.67)	50.37	-0.82 (-1.02 to -0.62)
North Africa and Middle East	32.38	49101.4 (49084.08 to 49118.72)	76.17	48113.93 (48103.11 to 48124.76)	135.24	-0.08 (-0.09 to -0.07)
High-income North America	12.01	15528.67 (15519.86 to 15537.49)	10.93	12637.39 (12629.89 to 12644.9)	-8.99	-0.76 (-0.9 to -0.63)
Oceania	0.18	13480.59 (13417.16 to 13544.27)	0.44	13439.13 (13399.13 to 13479.22)	144.44	-0.01 (-0.01 to 0)
Central Sub-Saharan Africa	3.39	33061.43 (33025.26 to 33097.64)	9.15	33106.35 (33084.51 to 33128.2)	169.91	0 (0 to 0.01)
Eastern Sub-Saharan Africa	10.15	29185.56 (29167 to 29204.14)	25.96	29181.44 (29169.95 to 29192.94)	155.76	0.03 (0.01 to 0.04)
Southern Sub-Saharan Africa	3.38	30923.15 (30889.37 to 30956.96)	6.52	30913.96 (30890.11 to 30937.83)	92.9	-0.01 (-0.01 to 0)
Western Sub-Saharan Africa	11.11	31841 (31821.67 to 31860.34)	31.31	31716.58 (31705.24 to 31727.93)	181.82	-0.01 (-0.01 to -0.01)

S12 Table. The global prevalence of uterine fibroids among women aged 15-49 years in 1990 and 2021, along with the trends and changes observed between these years, by country and territories

**Countries**	Number of cases (Thousands) in 1990	Age-standardized rate per 100,000 population (95% UI) in 2021	Number of cases (Thousands) in 2021	Age-standardized rate per 100,000 population (95% UI) in 2021	Estimated annual percentage changes (95% CI) from 1990 to 2021
Afghanistan	30.33	1767.36 (1746.8 to 1788.14)	103.47	1901.32 (1889.42 to 1913.27)	0.34 (0.29 to 0.39)
Albania	28.56	4248.89 (4198.2 to 4300.08)	28.21	4446.4 (4394.46 to 4498.83)	0.14 (0.12 to 0.15)
Algeria	89.92	2054.22 (2040.39 to 2068.12)	254.19	2154.73 (2146.34 to 2163.15)	0.18 (0.17 to 0.19)
American Samoa	0.24	2437.25 (2129.6 to 2780.75)	0.3	2606.62 (2316.94 to 2924.12)	0.21 (0.18 to 0.24)
Andorra	0.86	5821.21 (5436.33 to 6227.76)	1.55	6026.73 (5719.77 to 6349.85)	0.08 (0.05 to 0.11)
Angola	80.27	4415.55 (4384.35 to 4446.94)	300.6	4838.44 (4820.89 to 4856.04)	0.31 (0.29 to 0.33)
Antigua and Barbuda	0.88	6248.99 (5837.31 to 6684.23)	1.67	6454.09 (6147.52 to 6773.17)	0.04 (0.02 to 0.06)
Argentina	355.42	4651.63 (4636.33 to 4666.96)	600.05	4936.87 (4924.37 to 4949.38)	0.19 (0.14 to 0.23)
Armenia	50.26	6896.74 (6834.34 to 6959.62)	60.23	7324.15 (7265.3 to 7383.42)	0.23 (0.21 to 0.24)
Australia	63.99	1425.48 (1414.42 to 1436.6)	96.55	1457.63 (1448.42 to 1466.88)	0.06 (0.05 to 0.07)
Austria	127.87	6418.01 (6382.79 to 6453.4)	135.14	5984.12 (5952.03 to 6016.36)	-0.45 (-0.54 to -0.36)
Azerbaijan	96.38	6824.93 (6779.63 to 6870.49)	208.34	7291.44 (7260.02 to 7322.98)	0.26 (0.24 to 0.29)
Bahamas	3.9	6413.11 (6209.85 to 6621.71)	7.19	6527.14 (6376.9 to 6680.1)	0.02 (-0.01 to 0.06)
Bahrain	2.05	2122.42 (2024.78 to 2224.28)	7.18	2155.08 (2105.45 to 2205.62)	0.02 (0.01 to 0.03)
Bangladesh	531.64	2956.46 (2948.27 to 2964.66)	1372.53	3218.96 (3213.56 to 3224.36)	0.33 (0.3 to 0.36)
Barbados	4.06	6432.49 (6233.73 to 6636.36)	5.11	6546.03 (6366.52 to 6729.7)	0.01 (-0.02 to 0.03)
Belarus	233.98	9453.72 (9415.25 to 9492.32)	251.42	9710.05 (9671.61 to 9748.64)	0.14 (0.12 to 0.16)
Belgium	147.24	5907.99 (5877.75 to 5938.35)	167.71	6049.17 (6020.1 to 6078.36)	-0.05 (-0.13 to 0.03)
Belize	1.91	6313.67 (6022.86 to 6616.18)	7.06	6567.31 (6414.46 to 6722.98)	0.06 (0 to 0.11)
Benin	37.67	4406.3 (4360.41 to 4452.59)	121.48	4767.59 (4740.26 to 4795.05)	0.25 (0.2 to 0.31)
Bermuda	1.2	6712.26 (6336.07 to 7107.54)	1.11	6843.45 (6439.85 to 7270.11)	0.01 (-0.02 to 0.04)
Bhutan	3.14	3046.14 (2937.24 to 3158.34)	6.52	3380.02 (3297.84 to 3463.84)	0.38 (0.36 to 0.4)
Bolivia (Plurinational State of)	113.54	9052.63 (8999.35 to 9106.17)	269.98	9304.84 (9269.66 to 9340.13)	0.06 (0.04 to 0.08)
Bosnia and Herzegovina	45.22	4135.78 (4097.56 to 4174.29)	38.3	4493.18 (4447.53 to 4539.25)	0.31 (0.28 to 0.34)
Botswana	20.27	8442.13 (8322.63 to 8563.05)	59.6	9027.95 (8955.06 to 9101.32)	0.45 (0.15 to 0.75)
Brazil	1086.99	3163.97 (3157.92 to 3170.03)	2941.91	4696.74 (4691.37 to 4702.12)	1.23 (1.14 to 1.33)
Brunei Darussalam	2.53	4158.19 (3990.55 to 4332.19)	5.73	4395.95 (4282.75 to 4511.61)	0.19 (0.16 to 0.22)
Bulgaria	100.97	4503.29 (4475.4 to 4531.33)	81.24	4620.24 (4587.52 to 4653.18)	0.13 (0.12 to 0.14)
Burkina Faso	72.02	4299.05 (4267.19 to 4331.11)	198.87	4614.12 (4593.32 to 4635.01)	0.21 (0.16 to 0.26)
Burundi	32.67	3288.95 (3252.13 to 3326.13)	80.26	3267.21 (3243.77 to 3290.79)	-0.03 (-0.04 to -0.01)
Cabo Verde	2.53	4427.48 (4248.32 to 4612.82)	6.7	4771.05 (4656.3 to 4888.09)	0.22 (0.18 to 0.27)
Cambodia	48.64	2331.76 (2310.56 to 2353.12)	109.11	2541.63 (2526.45 to 2556.88)	0.28 (0.27 to 0.29)
Cameroon	86.75	4725.55 (4693.37 to 4757.91)	305.63	4837.82 (4820.28 to 4855.42)	0.07 (0.04 to 0.09)
Canada	174.92	2278.7 (2268 to 2289.46)	210.98	2279.03 (2269.29 to 2288.82)	0.08 (-0.14 to 0.3)
Central African Republic	22.85	4529.44 (4469.67 to 4589.84)	51.69	4578.83 (4538.9 to 4619.05)	-0.02 (-0.04 to 0.01)
Chad	42.05	3984.56 (3945.77 to 4023.66)	124.72	4310.35 (4285.81 to 4335)	0.22 (0.18 to 0.27)
Chile	145.28	4557.78 (4534.19 to 4581.46)	245.34	4991.22 (4971.47 to 5011.04)	-0.04 (-0.3 to 0.23)
China	6245.51	2266.3 (2264.49 to 2268.11)	9006.44	2422.13 (2420.53 to 2423.73)	0.11 (-0.04 to 0.25)
Colombia	474.56	6967.02 (6946.84 to 6987.24)	899.55	6995.39 (6980.93 to 7009.87)	-0.05 (-0.07 to -0.02)
Comoros	2.86	3534.72 (3403.53 to 3669.95)	6.22	3540.26 (3452.45 to 3629.78)	-0.02 (-0.05 to 0.01)
Congo	19.57	4654.17 (4587.34 to 4721.8)	62.8	4841.55 (4803.56 to 4879.78)	0.1 (0.09 to 0.12)
Cook Islands	0.1	2465.37 (1991.36 to 3022.58)	0.11	2663.99 (2193.82 to 3209.16)	0.24 (0.21 to 0.26)

Costa Rica	43.73	7046.9 (6979.49 to 7114.85)	94.59	7165.5 (7119.83 to 7211.4)	0 (-0.03 to 0.02)
Côte d'Ivoire	91.46	4498.96 (4468.7 to 4529.38)	270.12	4819.97 (4801.39 to 4838.62)	0.22 (0.17 to 0.26)
Croatia	56.64	4544.95 (4507.53 to 4582.61)	49.6	4772.42 (4729.89 to 4815.3)	0.17 (0.11 to 0.24)
Cuba	178.63	6665.39 (6634.32 to 6696.58)	188.28	6874.32 (6842.82 to 6905.95)	0.06 (0.03 to 0.08)
Cyprus	10.97	5596.43 (5492.09 to 5702.29)	25.5	5926.86 (5853.67 to 6001.01)	-0.07 (-0.27 to 0.13)
Czechia	149.19	5283.41 (5256.39 to 5310.54)	158.35	5439.88 (5412.04 to 5467.86)	0.2 (0.12 to 0.28)
Democratic People's Republic of Korea	89.88	1788.19 (1776.44 to 1800)	128.46	1869.63 (1859.39 to 1879.92)	0.15 (0.14 to 0.17)
Democratic Republic of the Congo	291.05	4378.89 (4362.62 to 4395.21)	776.58	4564.52 (4554.19 to 4574.86)	0.13 (0.1 to 0.16)
Denmark	65.59	4703.11 (4666.97 to 4739.47)	63.86	4707.39 (4670.58 to 4744.44)	-0.01 (-0.14 to 0.13)
Djibouti	2.55	3413.74 (3278.84 to 3553.23)	11.57	3685.62 (3618.29 to 3753.96)	0.27 (0.24 to 0.31)
Dominica	0.84	6193.68 (5778.11 to 6632.12)	1.03	6452.21 (6064.1 to 6859)	0.06 (0.02 to 0.1)
Dominican Republic	88.98	6162.7 (6121.19 to 6204.45)	179.43	6571.26 (6540.83 to 6601.8)	0.18 (0.15 to 0.22)
Ecuador	188	9383.58 (9340.41 to 9426.91)	414.76	9275.44 (9247.19 to 9303.76)	-0.07 (-0.1 to -0.04)
Egypt	234.58	2102.39 (2093.8 to 2111)	526.31	2186.42 (2180.48 to 2192.37)	0.07 (0.06 to 0.09)
El Salvador	66.65	6772.85 (6720.86 to 6825.17)	117.12	7015.54 (6975.34 to 7055.92)	0.08 (0.06 to 0.09)
Equatorial Guinea	3.52	4424.53 (4277.37 to 4575.69)	15.12	5024.67 (4943.19 to 5107.26)	0.44 (0.37 to 0.52)
Eritrea	19.99	3194.62 (3149.63 to 3240.12)	47.33	3333.83 (3303.52 to 3364.37)	0.15 (0.11 to 0.18)
Estonia	38.25	9520.17 (9424.9 to 9616.21)	32.67	9893.46 (9785.38 to 10002.63)	0.17 (0.15 to 0.18)
Eswatini	12.57	8701.65 (8546.1 to 8859.53)	23.77	8828.13 (8713.73 to 8943.79)	0.03 (-0.01 to 0.08)
Ethiopia	299.63	3359.86 (3347.47 to 3372.3)	782.64	3548.95 (3540.87 to 3557.05)	0.22 (0.2 to 0.23)
Fiji	4.05	2386.56 (2312.73 to 2462.28)	5.74	2572.55 (2506.36 to 2640.1)	0.22 (0.2 to 0.25)
Finland	76.2	5368.69 (5330.32 to 5407.31)	67.88	5413.58 (5372.8 to 5454.62)	-0.34 (-0.54 to -0.15)
France	1047.61	7169.05 (7155.23 to 7182.89)	1143.43	7260.16 (7246.76 to 7273.58)	0.06 (-0.02 to 0.15)
Gabon	7.73	4722.01 (4613.25 to 4832.9)	20.85	5029.94 (4961.23 to 5099.41)	0.19 (0.16 to 0.22)
Gambia	7.59	4547.04 (4439.9 to 4656.43)	23.03	4793.76 (4730.27 to 4857.97)	0.13 (0.09 to 0.17)
Georgia	90.29	7012.87 (6966.95 to 7059.02)	73.65	8080.54 (8021.94 to 8139.53)	0.61 (0.53 to 0.68)
Germany	1469.63	7316.66 (7304.82 to 7328.52)	1449.04	7465.22 (7453.02 to 7477.44)	0.12 (0.02 to 0.22)
Ghana	121.53	4362.23 (4337.07 to 4387.5)	364.75	4580.09 (4565.05 to 4595.17)	0.1 (0.05 to 0.15)
Greece	153.1	5996.59 (5966.56 to 6026.73)	165.36	6158.67 (6128.22 to 6189.25)	0.07 (0.04 to 0.11)
Greenland	0.4	3056.68 (2757.64 to 3380.65)	0.4	3150.94 (2846.48 to 3480.34)	0.08 (0.06 to 0.09)
Grenada	0.93	6055.04 (5661.34 to 6470.87)	1.58	6434.66 (6120.06 to 6761.78)	0.11 (0.08 to 0.15)
Guam	0.77	2463.34 (2288.64 to 2649.45)	0.96	2659.12 (2492.54 to 2834.23)	0.26 (0.24 to 0.28)
Guatemala	91.86	6617.3 (6573.9 to 6660.93)	249.66	6711.93 (6685.45 to 6738.49)	0.02 (0 to 0.03)
Guinea	49.64	4276.42 (4238.29 to 4314.84)	119.52	4485.56 (4459.49 to 4511.75)	0.16 (0.14 to 0.18)
Guinea-Bissau	7.95	4343.65 (4246.33 to 4442.81)	19.26	4461.48 (4396.94 to 4526.82)	0.07 (0.03 to 0.11)
Guyana	9.75	6148.19 (6023.52 to 6275)	11.99	6455.86 (6340.31 to 6573.03)	0.13 (0.1 to 0.16)
Haiti	71.94	5737.96 (5695.24 to 5780.93)	191.8	5859.6 (5833.19 to 5886.11)	0.03 (0.01 to 0.06)
Honduras	52.02	6688.4 (6629.93 to 6747.28)	167.92	7008.46 (6974.73 to 7042.32)	0.12 (0.1 to 0.14)
Hungary	123.2	4460.89 (4435.88 to 4486.03)	120.78	4553.81 (4527.21 to 4580.56)	0.09 (0.07 to 0.11)
Iceland	3.65	5991.35 (5797.49 to 6190.31)	5.08	6004.86 (5840.47 to 6173.02)	-0.19 (-0.31 to -0.07)
India	7551.03	4387.9 (4384.74 to 4391.06)	19869.49	5604.8 (5602.33 to 5607.27)	1.03 (0.9 to 1.16)
Indonesia	870.83	2133.8 (2129.23 to 2138.37)	1581.6	2063.07 (2059.86 to 2066.29)	-0.25 (-0.35 to -0.14)
Iran (Islamic Republic of)	240.06	2429.94 (2419.91 to 2440)	642.61	2451.78 (2445.72 to 2457.86)	-0.67 (-0.97 to -0.36)
Iraq	65.81	2114.14 (2097.59 to 2130.81)	199.63	2117.41 (2108.09 to 2126.76)	0.01 (-0.02 to 0.05)
Ireland	49.45	5934.59 (5882.25 to 5987.3)	81.37	5970.95 (5929.34 to 6012.81)	-0.01 (-0.02 to 0.01)
Israel	65.89	5788.64 (5743.91 to 5833.66)	133.32	5884.86 (5853.26 to 5916.6)	0.03 (0.01 to 0.05)

Italy	742.98	5171.55 (5159.77 to 5183.35)	737.86	5253.44 (5241.07 to 5265.83)	0.04 (-0.04 to 0.12)
Jamaica	28.96	6329.3 (6254.69 to 6404.64)	48.97	6595.45 (6537.04 to 6654.27)	0.1 (0.08 to 0.12)
Japan	2327.24	6956.68 (6947.59 to 6965.79)	2057.01	7459.26 (7448.76 to 7469.77)	0.19 (0.11 to 0.27)
Jordan	12.49	2094.8 (2057.07 to 2133.1)	60.29	2166.53 (2149.21 to 2183.97)	0.12 (0.11 to 0.13)
Kazakhstan	250.7	7010.5 (6982.52 to 7038.56)	370.11	7371.78 (7348 to 7395.63)	0.18 (0.17 to 0.2)
Kenya	140.22	3761.05 (3740.54 to 3781.65)	414.27	3817.99 (3806.12 to 3829.88)	0.09 (0.07 to 0.12)
Kiribati	0.36	2310.45 (2073.14 to 2569.62)	0.76	2578.08 (2396.52 to 2770.55)	0.34 (0.29 to 0.38)
Kuwait	7.75	2168.87 (2118.16 to 2220.69)	39.11	2233.32 (2210.84 to 2256.05)	0.13 (0.12 to 0.14)
Kyrgyzstan	52.91	6610.82 (6551.75 to 6670.34)	111.04	6893.91 (6853.23 to 6934.77)	0.14 (0.13 to 0.15)
Lao People's Democratic Republic	18.88	2379.12 (2344.64 to 2414)	47.96	2629.4 (2605.72 to 2653.25)	0.35 (0.33 to 0.36)
Latvia	77.25	11330.64 (11250.73 to 11411.01)	55.24	11729.95 (11630.98 to 11829.72)	0.14 (0.05 to 0.23)
Lebanon	14.21	2139.58 (2104.26 to 2175.36)	35.53	2236.39 (2213.07 to 2259.92)	0.13 (0.12 to 0.14)
Lesotho	26.68	8290.26 (8190.39 to 8391.08)	37.42	8905.68 (8814.07 to 8998.06)	0.22 (0.2 to 0.25)
Liberia	19.09	4455.65 (4389.88 to 4522.26)	55.57	4683.24 (4644.01 to 4722.74)	0.2 (0.18 to 0.23)
Libya	13.75	2109.69 (2073.43 to 2146.47)	43.5	2095.63 (2075.94 to 2115.46)	0 (-0.01 to 0.01)
Lithuania	70.51	7633.08 (7576.79 to 7689.69)	51.97	7896.77 (7828.22 to 7965.87)	-0.04 (-0.1 to 0.02)
Luxembourg	7.61	7482.56 (7315.05 to 7653.44)	13.76	7633.04 (7505.34 to 7762.79)	-0.31 (-0.44 to -0.18)
Madagascar	69.8	3396.52 (3370.56 to 3422.65)	196.74	3366.08 (3350.98 to 3381.24)	0 (-0.02 to 0.01)
Malawi	60.59	3562.49 (3533.27 to 3591.91)	133.02	3488.28 (3468.98 to 3507.67)	-0.02 (-0.05 to 0)
Malaysia	102.25	2632.54 (2616.06 to 2649.1)	233.53	2870.31 (2858.63 to 2882.03)	0.26 (0.23 to 0.3)
Maldives	0.85	2412.21 (2245.74 to 2588.49)	3.42	2794.7 (2700.83 to 2891.29)	0.58 (0.54 to 0.63)
Mali	63.44	4066.14 (4033.98 to 4098.51)	183.17	4482.05 (4460.95 to 4503.24)	0.29 (0.25 to 0.33)
Malta	5.24	5048.6 (4911.91 to 5188.44)	5.81	5254.24 (5119.03 to 5392.86)	0 (-0.12 to 0.12)
Marshall Islands	0.17	2213.19 (1874.96 to 2604.89)	0.34	2444.8 (2191.45 to 2720.34)	0.28 (0.25 to 0.31)
Mauritania	16.84	4541.75 (4471.68 to 4612.73)	41.4	4796.74 (4749.94 to 4843.91)	0.14 (0.09 to 0.19)
Mauritius	7.08	2602.88 (2541.47 to 2665.55)	9.35	2816.86 (2759.67 to 2874.99)	0.24 (0.21 to 0.26)
Mexico	1380.85	8325.66 (8311.53 to 8339.81)	2740.52	7785.92 (7776.71 to 7795.15)	-0.24 (-0.27 to -0.2)
Micronesia (Federated States of)	0.42	2328.49 (2104.89 to 2572.03)	0.58	2512.81 (2312.17 to 2726.45)	0.2 (0.16 to 0.24)
Monaco	0.5	5993.12 (5470.99 to 6565.46)	0.52	6013.39 (5493.76 to 6575.52)	0.02 (-0.01 to 0.04)
Mongolia	24.42	6693.89 (6606.64 to 6782.07)	62.99	7126.65 (7071.01 to 7182.64)	0.21 (0.2 to 0.22)
Montenegro	6.61	4437.41 (4330.75 to 4546.13)	7.49	4630.38 (4525.15 to 4737.66)	0.21 (0.19 to 0.22)
Morocco	104.54	2031.51 (2018.98 to 2044.09)	201.17	2080.62 (2071.54 to 2089.74)	0.06 (0.05 to 0.07)
Mozambique	87	3347.5 (3324.97 to 3370.14)	204.88	3548.49 (3532.77 to 3564.26)	0.22 (0.2 to 0.23)
Myanmar	204.83	2381.96 (2371.38 to 2392.57)	384.26	2630.32 (2622 to 2638.66)	0.37 (0.35 to 0.4)
Namibia	21.22	8384.59 (8269.14 to 8501.36)	50.13	8612.56 (8536.65 to 8689.02)	0.1 (0.07 to 0.12)
Nauru	0.05	2379.91 (1755.05 to 3175.37)	0.07	2581.11 (1987.65 to 3306.82)	0.23 (0.22 to 0.25)
Nepal	81.82	2206.26 (2190.95 to 2221.66)	174.01	2179.95 (2169.65 to 2190.29)	-0.17 (-0.25 to -0.1)
Netherlands	237.28	5884.97 (5861.24 to 5908.79)	235.89	5937.66 (5913.56 to 5961.83)	0.01 (0 to 0.02)
New Zealand	13.74	1561.48 (1535.41 to 1587.9)	17.07	1359.08 (1338.73 to 1379.68)	-0.65 (-0.71 to -0.58)
Nicaragua	42.83	6718.91 (6653.72 to 6784.63)	117.52	7017.07 (6976.87 to 7057.45)	0.1 (0.08 to 0.13)
Niger	54.47	4049.82 (4015.04 to 4084.85)	163.35	4289.05 (4267.7 to 4310.5)	0.21 (0.18 to 0.24)
Nigeria	710.81	4750.79 (4739.37 to 4762.24)	2191.78	4873.89 (4867.32 to 4880.47)	0.03 (-0.01 to 0.07)
Niue	0.01	2424.62 (1214.65 to 4345.61)	0.01	2641.2 (1275.81 to 4898.8)	0.26 (0.23 to 0.29)
North Macedonia	21.15	4274.31 (4216.8 to 4332.43)	27.22	4505.24 (4451.25 to 4559.83)	0.19 (0.18 to 0.2)
Northern Mariana Islands	0.29	2436.85 (2152.28 to 2754.45)	0.31	2572.75 (2283.68 to 2890.66)	0.14 (0.12 to 0.17)
Norway	47.16	4367.93 (4328.5 to 4407.63)	57.82	4387.2 (4351.35 to 4423.29)	-0.6 (-1.02 to -0.18)

Oman	5.6	2003.3 (1949.61 to 2058.23)	22.72	2141.34 (2113.03 to 2169.98)	0.24 (0.24 to 0.25)
Pakistan	662.43	3500.18 (3491.67 to 3508.7)	1900.74	3584.26 (3579.12 to 3589.41)	0.12 (0.09 to 0.15)
Palau	0.09	2440.41 (1955.43 to 3018.12)	0.11	2645.55 (2160.11 to 3220.62)	0.22 (0.2 to 0.25)
Palestine	6.55	2015.81 (1965.85 to 2066.78)	22.5	2056.05 (2028.89 to 2083.51)	0.06 (0.05 to 0.08)
Panama	32.61	6655.93 (6583.06 to 6729.46)	72.64	7022.46 (6971.46 to 7073.75)	0.11 (0.1 to 0.13)
Papua New Guinea	17.33	2217.91 (2184.31 to 2251.93)	57.25	2407.72 (2387.89 to 2427.68)	0.2 (0.17 to 0.24)
Paraguay	28.46	3674.52 (3631.21 to 3718.24)	68.69	3891.85 (3862.64 to 3921.24)	0.22 (0.21 to 0.24)
Peru	391.08	9016.17 (8987.47 to 9044.94)	886.2	9326.86 (9307.43 to 9346.32)	0.09 (0.07 to 0.11)
Philippines	435.34	3386.08 (3375.81 to 3396.38)	1031.43	3828.14 (3820.72 to 3835.57)	0.44 (0.39 to 0.48)
Poland	352.96	3649.53 (3637.31 to 3661.77)	302.59	2946.02 (2935.2 to 2956.87)	-1.04 (-1.38 to -0.7)
Portugal	114.43	4580.55 (4554.03 to 4607.2)	118.73	4162.46 (4138.07 to 4186.98)	-0.53 (-0.66 to -0.4)
Puerto Rico	61	6584.16 (6531.95 to 6636.68)	55.32	6790.7 (6733.78 to 6848.01)	0.06 (0.03 to 0.09)
Qatar	1.62	2119.77 (2011.59 to 2233.37)	13.87	2177.13 (2139.73 to 2215.17)	0.08 (0.07 to 0.09)
Republic of Korea	357.17	3059.95 (3049.77 to 3070.15)	421.17	3239.77 (3229.78 to 3249.79)	0.2 (0.02 to 0.39)
Republic of Moldova	98.58	8995.26 (8938.7 to 9052.1)	100.92	9475.25 (9416.19 to 9534.69)	0.23 (0.19 to 0.27)
Romania	219.19	3946.47 (3929.92 to 3963.08)	204.68	4164.75 (4146.21 to 4183.37)	0.25 (0.18 to 0.31)
Russian Federation	3838.71	10289.2 (10278.78 to 10299.63)	4269.22	10383.65 (10373.64 to 10393.68)	0.06 (0.04 to 0.07)
Rwanda	43.53	3500.48 (3466.33 to 3534.92)	107.01	3576.55 (3554.83 to 3598.39)	0.14 (0.12 to 0.16)
Saint Kitts and Nevis	0.49	6385.06 (5800.91 to 7019.79)	1.11	6668.11 (6280.18 to 7075.28)	0.08 (0.05 to 0.11)
Saint Lucia	1.62	6212.57 (5906.2 to 6531.79)	3.22	6527.16 (6302.62 to 6758.3)	0.05 (0.01 to 0.1)
Saint Vincent and the Grenadines	1.18	6082.75 (5729.19 to 6454.18)	1.86	6501.16 (6208.03 to 6805.09)	0.15 (0.11 to 0.18)
Samoa	0.69	2464.84 (2280.53 to 2660.89)	1.12	2656.46 (2502.06 to 2818.16)	0.21 (0.18 to 0.25)
San Marino	0.36	5990.65 (5385.62 to 6645.96)	0.52	6000.03 (5474.3 to 6568.41)	0 (-0.01 to 0.02)
Sao Tome and Principe	0.83	4371.3 (4070.02 to 4690.28)	2.2	4585.55 (4394.6 to 4783.05)	0.15 (0.11 to 0.19)
Saudi Arabia	50.8	2003.52 (1985.59 to 2021.59)	232.54	2119.16 (2110.49 to 2127.86)	0.2 (0.19 to 0.2)
Senegal	59.98	4580.86 (4543.29 to 4618.69)	147.78	4621.35 (4597.44 to 4645.36)	-0.01 (-0.04 to 0.02)
Serbia	97.58	4123.64 (4097.8 to 4149.61)	100.21	4352.83 (4325.64 to 4380.16)	0.28 (0.23 to 0.33)
Seychelles	0.38	2608.04 (2342.66 to 2897.07)	0.72	2792.71 (2591.03 to 3006.59)	0.2 (0.17 to 0.22)
Sierra Leone	35.4	4464.72 (4416.44 to 4513.44)	85.37	4793.13 (4760.1 to 4826.36)	0.22 (0.19 to 0.24)
Singapore	36.77	3990.3 (3949.32 to 4031.62)	75.37	4242.14 (4211 to 4273.55)	0.2 (0.17 to 0.22)
Slovakia	70.23	5265.29 (5226.3 to 5304.51)	82.49	5392.83 (5355.2 to 5430.71)	-0.14 (-0.33 to 0.05)
Slovenia	22.32	4441.02 (4382.88 to 4499.77)	23.68	4603.96 (4543.88 to 4664.78)	0.17 (0.05 to 0.3)
Solomon Islands	1.24	2235.85 (2109.8 to 2367.94)	3.76	2460.58 (2382.24 to 2540.94)	0.24 (0.19 to 0.28)
Somalia	48.27	3347.88 (3317.58 to 3378.41)	129.07	3369.47 (3350.76 to 3388.26)	0.06 (0.04 to 0.08)
South Africa	772.89	9881.01 (9858.55 to 9903.51)	1536.24	9930.5 (9914.74 to 9946.28)	0.03 (0.01 to 0.05)
South Sudan	32.23	3364.38 (3326.35 to 3402.77)	65.75	3387.73 (3361.66 to 3413.96)	0.02 (0 to 0.04)
Spain	678.72	7366.29 (7348.76 to 7383.86)	931.88	7423.64 (7408.03 to 7439.28)	0.09 (-0.01 to 0.18)
Sri Lanka	102.72	2475.76 (2460.49 to 2491.11)	153.86	2652.18 (2638.91 to 2665.51)	0.21 (0.19 to 0.24)
Sudan	64.98	1747.88 (1734.18 to 1761.68)	184.8	1908.49 (1899.66 to 1917.36)	0.31 (0.27 to 0.34)
Suriname	5.07	6351.09 (6175.82 to 6530.17)	9.76	6674.87 (6543.06 to 6808.7)	0.11 (0.09 to 0.13)
Sweden	99.33	4445.26 (4417.41 to 4473.24)	98.62	4085.98 (4060.42 to 4111.69)	0.33 (0.04 to 0.62)
Switzerland	107.96	5739.67 (5705.4 to 5774.11)	131.17	5749.26 (5717.99 to 5780.69)	0 (-0.01 to 0.02)
Syrian Arab Republic	41.91	2056.29 (2036.04 to 2076.7)	76.94	2101.34 (2085.93 to 2116.86)	0.06 (0.05 to 0.07)
Taiwan (Province of China)	101.55	1999.96 (1987.42 to 2012.57)	145.38	2167.2 (2155.85 to 2178.6)	0.31 (0.29 to 0.33)
Tajikistan	54.81	6638.19 (6578.84 to 6697.99)	151.42	6742.4 (6708.09 to 6776.86)	0.04 (0.03 to 0.06)
Thailand	356.86	2526.26 (2517.86 to 2534.68)	509.4	2758.53 (2750.81 to 2766.28)	0.28 (0.25 to 0.31)

Timor-Leste	3.71	2331.29 (2255.06 to 2409.64)	6.98	2548.31 (2487.56 to 2610.23)	0.31 (0.29 to 0.33)
Togo	28.52	4320.31 (4268.91 to 4372.22)	86.92	4536.01 (4505.64 to 4566.55)	0.14 (0.11 to 0.17)
Tokelau	0.01	2362.45 (961.29 to 4953.94)	0.01	2599.3 (1129.61 to 5158.87)	0.3 (0.27 to 0.33)
Tonga	0.43	2458.47 (2230.14 to 2704.37)	0.59	2620.49 (2413.46 to 2840.85)	0.14 (0.1 to 0.18)
Trinidad and Tobago	17.19	6348.47 (6252.78 to 6445.35)	25.12	6655.3 (6572.83 to 6738.63)	0.11 (0.09 to 0.14)
Tunisia	34.23	2065.5 (2043.21 to 2088)	72.82	2166.38 (2150.61 to 2182.25)	0.15 (0.14 to 0.16)
Turkey	224.72	1883.38 (1875.49 to 1891.3)	453.77	1998.24 (1992.42 to 2004.08)	0.2 (0.17 to 0.24)
Turkmenistan	43.61	6814.89 (6747.27 to 6883.07)	85.28	7143.28 (7095.36 to 7191.44)	0.19 (0.18 to 0.2)
Tuvalu	0.05	2334.57 (1752.23 to 3056.23)	0.07	2555.66 (1974.76 to 3257.87)	0.26 (0.23 to 0.29)
Uganda	92.22	3361.06 (3338.51 to 3383.73)	270.71	3459.93 (3446.5 to 3473.4)	0.1 (0.09 to 0.11)
Ukraine	1323.52	10133.55 (10116.27 to 10150.86)	1260.29	10157.97 (10139.92 to 10176.05)	0.05 (0.03 to 0.07)
United Arab Emirates	6.09	2032.31 (1976.91 to 2089.21)	48.81	2148.54 (2127.32 to 2169.99)	0.15 (0.13 to 0.17)
United Kingdom	727.26	5006.83 (4995.29 to 5018.4)	704.69	4163.29 (4153.55 to 4173.05)	-0.19 (-0.39 to 0.02)
United Republic of Tanzania	157.07	3547.1 (3528.98 to 3565.3)	435.64	3624.49 (3613.56 to 3635.45)	0.1 (0.09 to 0.12)
United States of America	2796.67	4061.24 (4056.46 to 4066.02)	3944.2	4933.22 (4928.35 to 4938.1)	0.59 (0.26 to 0.92)
United States Virgin Islands	1.92	6420.65 (6135.68 to 6716.02)	1.28	6616.83 (6255.07 to 6996.17)	0.06 (0.02 to 0.09)
Uruguay	33.9	4651.53 (4602.14 to 4701.33)	43.8	5016.88 (4969.87 to 5064.24)	0.24 (0.19 to 0.29)
Uzbekistan	226.95	6481.62 (6453.13 to 6510.22)	626.04	7056.18 (7038.67 to 7073.72)	0.28 (0.26 to 0.29)
Vanuatu	0.64	2275.17 (2098.13 to 2464.47)	1.7	2464.9 (2348.36 to 2585.95)	0.25 (0.23 to 0.26)
Venezuela (Bolivarian Republic of)	275.85	7175.57 (7148.2 to 7203.03)	536.98	7171.03 (7151.81 to 7190.29)	-0.05 (-0.07 to -0.03)
Vietnam	309.92	2262.37 (2254 to 2270.77)	706.44	2578.46 (2572.43 to 2584.49)	0.5 (0.47 to 0.53)
Yemen	41.65	1895.02 (1876.25 to 1913.95)	140.54	1952.47 (1942.08 to 1962.9)	0.14 (0.11 to 0.16)
Zambia	47.04	3626.58 (3592.77 to 3660.65)	141.23	3674.16 (3654.46 to 3693.95)	0.04 (0.03 to 0.05)
Zimbabwe	150.5	8636.34 (8591.26 to 8681.61)	293.56	8592.89 (8561.4 to 8624.46)	-0.04 (-0.07 to -0.01)

S13 Table. The global prevalence of polycystic ovarian syndrome among women aged 15-49 years in 1990 and 2021, along with the trends and changes observed between these years, by country and territories

Countries	Number of cases (Thousands) in 1990	Age-standardized rate per 100,000 population (95% UI) in 2021	Number of cases (Thousands) in 2021	Age-standardized rate per 100,000 population (95% UI) in 2021	Estimated annual percentage changes (95% CI) from 1990 to 2021
Afghanistan	37.14	1726.17 (1707.05 to 1745.5)	177.43	2478.45 (2466.2 to 2490.76)	1.68 (1.41 to 1.95)
Albania	2.31	277.04 (265.47 to 289.06)	2.24	364.64 (349.65 to 380.14)	0.86 (0.76 to 0.95)
Algeria	167.73	2877.85 (2863.24 to 2892.53)	473.94	4210.8 (4198.77 to 4222.87)	1.4 (1.36 to 1.44)
American Samoa	0.46	3844.68 (3489.66 to 4231.88)	0.61	5315.09 (4899 to 5758.7)	0.9 (0.72 to 1.08)
Andorra	0.83	5517.26 (5145.39 to 5910.63)	1.36	6826.34 (6450.89 to 7220.44)	0.64 (0.54 to 0.74)
Angola	17.77	784.05 (772.09 to 796.17)	107.22	1408.14 (1399.45 to 1416.88)	1.8 (1.65 to 1.94)
Antigua and Barbuda	0.37	2249.5 (2021.85 to 2499.18)	0.72	2995.22 (2779.74 to 3223.86)	0.81 (0.76 to 0.86)
Argentina	179	2241.9 (2231.5 to 2252.33)	411.9	3453.91 (3443.36 to 3464.48)	1.35 (1.18 to 1.52)
Armenia	5.38	614.42 (597.62 to 631.64)	6.79	912.9 (890.77 to 935.51)	1.43 (1.38 to 1.48)
Australia	328.29	7325.25 (7300.17 to 7350.39)	534.96	8825.38 (8801.52 to 8849.29)	0.38 (0.26 to 0.49)
Austria	141.88	7119.85 (7082.64 to 7157.22)	149.42	7522.91 (7484.18 to 7561.82)	-0.05 (-0.13 to 0.02)
Azerbaijan	11.83	626.1 (614.21 to 638.2)	26.69	965.29 (953.59 to 977.11)	1.73 (1.61 to 1.84)
Bahamas	2.09	2855.31 (2731.25 to 2984.23)	3.66	3406.3 (3296.7 to 3518.66)	0.52 (0.45 to 0.59)
Bahrain	4.7	4014.39 (3893.05 to 4139.48)	14.96	4576.31 (4503.13 to 4650.42)	0.4 (0.36 to 0.45)
Bangladesh	173.22	714.99 (711.42 to 718.58)	543.25	1178.95 (1175.8 to 1182.1)	1.87 (1.74 to 2)
Barbados	1.95	2816.4 (2691.5 to 2946.14)	2.35	3315.12 (3181.27 to 3453.4)	0.47 (0.44 to 0.5)
Belarus	9.78	382.6 (375.01 to 390.3)	10.95	509.77 (499.88 to 519.82)	1.12 (1.04 to 1.2)
Belgium	140.15	5712.68 (5682.69 to 5742.79)	168.22	6767.98 (6735.36 to 6800.74)	0.43 (0.24 to 0.61)
Belize	0.94	2231.42 (2081.34 to 2391.73)	3.9	3222.8 (3121.44 to 3326.82)	0.97 (0.71 to 1.23)
Benin	9.6	879.7 (861.33 to 898.43)	52.04	1609.02 (1594.64 to 1623.5)	1.85 (1.58 to 2.11)
Bermuda	0.59	3411.46 (3138.02 to 3704.68)	0.51	3796.81 (3463.54 to 4156.25)	0.24 (0.18 to 0.3)
Bhutan	1.45	1027.26 (971.66 to 1085.75)	3.8	1821.31 (1763.46 to 1880.72)	2.11 (1.99 to 2.23)
Bolivia (Plurinational State of)	64.63	4240.99 (4207.47 to 4274.74)	178.51	5700.83 (5674.3 to 5727.47)	1 (0.92 to 1.07)
Bosnia and Herzegovina	2.74	234.82 (226.08 to 243.83)	2.67	364.96 (351 to 379.39)	1.58 (1.37 to 1.8)
Botswana	4.19	1317.72 (1275.84 to 1360.85)	14.15	2065.86 (2031.81 to 2100.38)	1.67 (1.46 to 1.87)
Brazil	411.78	1057.39 (1054.1 to 1060.69)	672.52	1140.87 (1138.13 to 1143.61)	-0.21 (-0.38 to -0.04)
Brunei Darussalam	3.19	4712.57 (4543.65 to 4887.48)	9.32	7372.66 (7223.21 to 7524.6)	1.51 (1.36 to 1.66)
Bulgaria	6.74	325.52 (317.75 to 333.44)	5.93	417.34 (406.34 to 428.58)	0.82 (0.78 to 0.85)
Burkina Faso	17.18	829.43 (816.64 to 842.39)	72.54	1324.72 (1314.67 to 1334.84)	1.29 (1.09 to 1.49)
Burundi	10.45	837 (820.27 to 854.04)	27.32	885.53 (874.54 to 896.65)	0.09 (0.02 to 0.17)
Cabo Verde	0.75	957.88 (885.36 to 1035.83)	2.42	1602.38 (1538.69 to 1668.26)	1.45 (1.24 to 1.65)
Cambodia	55.63	2236.21 (2217.03 to 2255.54)	175.04	3856.08 (3837.92 to 3874.31)	1.88 (1.85 to 1.92)
Cameroon	31.22	1316.44 (1301.17 to 1331.87)	138.28	1765.07 (1755.44 to 1774.75)	0.74 (0.64 to 0.83)
Canada	206.44	2768.59 (2756.57 to 2780.65)	295.93	3548.83 (3535.93 to 3561.78)	0.72 (0.62 to 0.82)
Central African Republic	6.48	1020.13 (994.41 to 1046.42)	15.13	1105.79 (1087.69 to 1124.15)	0.05 (-0.07 to 0.17)
Chad	8.54	653.68 (639.35 to 668.29)	37.89	993.42 (982.85 to 1004.08)	0.89 (0.66 to 1.13)
Chile	85.93	2375.5 (2359.46 to 2391.63)	194.12	4089.67 (4071.43 to 4107.99)	1.71 (1.41 to 2.01)
China	5127.46	1604.41 (1602.99 to 1605.84)	9481.52	2962.19 (2960.24 to 2964.13)	2.07 (1.91 to 2.23)
Colombia	275.13	3154.13 (3141.99 to 3166.32)	544.01	4130.18 (4119.19 to 4141.19)	0.79 (0.73 to 0.85)
Comoros	1.3	1244.65 (1174.54 to 1318.39)	2.88	1478.13 (1424.17 to 1533.72)	0.31 (0.15 to 0.47)
Congo	6.07	1092.52 (1063.61 to 1122.14)	21.73	1521.54 (1501.19 to 1542.12)	0.9 (0.77 to 1.03)
Cook Islands	0.18	3965.54 (3397.26 to 4609.95)	0.25	5891.73 (5184.36 to 6672.77)	1.12 (0.99 to 1.26)

Costa Rica	30.74	3908.57 (3863.71 to 3953.9)	67.3	5168.31 (5129.23 to 5207.63)	0.8 (0.74 to 0.86)
Côte d'Ivoire	24.34	895.86 (883.91 to 907.96)	98.28	1474.51 (1465.06 to 1484.03)	1.41 (1.14 to 1.69)
Croatia	3.6	298.69 (288.99 to 308.64)	3.63	404.04 (390.75 to 417.7)	1.07 (0.98 to 1.17)
Cuba	75.59	2458.19 (2440.41 to 2476.07)	80.62	3265.07 (3242.26 to 3288.01)	0.94 (0.9 to 0.99)
Cyprus	8.69	4382.04 (4290.29 to 4475.3)	22.9	6328.57 (6243.46 to 6414.76)	1.31 (1.14 to 1.47)
Czechia	7.93	308.56 (301.73 to 315.5)	9.04	396.15 (387.64 to 404.82)	0.78 (0.73 to 0.84)
Democratic People's Republic of Korea	94.97	1679.74 (1668.93 to 1690.61)	124.32	1883.72 (1873.23 to 1894.26)	0.32 (0.24 to 0.41)
Democratic Republic of the Congo	70.43	837.29 (830.84 to 843.79)	257.73	1219.82 (1214.94 to 1224.72)	1.18 (1.03 to 1.34)
Denmark	63.41	4843.71 (4805.88 to 4881.78)	78.69	6232.53 (6188.72 to 6276.59)	0.79 (0.66 to 0.91)
Djibouti	0.96	986.8 (921.81 to 1055.94)	5.41	1675.58 (1631.03 to 1721.12)	1.81 (1.67 to 1.95)
Dominica	0.38	2234.97 (2008.93 to 2481.77)	0.5	3065.18 (2803.06 to 3345.75)	0.86 (0.74 to 0.97)
Dominican Republic	36.97	1954.38 (1933.55 to 1975.42)	89.88	3090.45 (3070.21 to 3110.8)	1.55 (1.46 to 1.64)
Ecuador	135.16	5346.2 (5316.73 to 5375.82)	333.59	7048.67 (7024.7 to 7072.71)	0.88 (0.65 to 1.1)
Egypt	456.39	3478.82 (3468.51 to 3489.16)	1146.7	4403.71 (4395.6 to 4411.84)	0.62 (0.56 to 0.69)
El Salvador	41.82	3193.15 (3161.35 to 3225.24)	80.76	4527.85 (4496.51 to 4559.37)	1.06 (0.95 to 1.16)
Equatorial Guinea	0.87	895.47 (835.04 to 959.58)	7.62	2090.19 (2042.07 to 2139.32)	2.97 (2.54 to 3.4)
Eritrea	5.74	739.81 (720.06 to 760.03)	18.27	1108.39 (1092.06 to 1124.92)	1.26 (1.07 to 1.46)
Estonia	1.57	408.09 (388.06 to 428.93)	1.62	578.11 (549.55 to 607.9)	1.42 (1.33 to 1.5)
Eswatini	3.35	1729.38 (1667.95 to 1792.87)	6.8	2145.1 (2093.22 to 2198.13)	0.42 (0.18 to 0.67)
Ethiopia	82.94	752.81 (747.45 to 758.2)	312.48	1144.76 (1140.57 to 1148.97)	1.56 (1.49 to 1.62)
Fiji	6.01	3068.05 (2989.57 to 3148.28)	10.71	4687.9 (4599.43 to 4777.69)	1.24 (1.1 to 1.37)
Finland	63.93	5010.52 (4971.22 to 5050.07)	72.41	6347.86 (6301.35 to 6394.65)	0.7 (0.65 to 0.74)
France	700.3	4821.71 (4810.39 to 4833.06)	858.88	6063.34 (6050.41 to 6076.28)	0.7 (0.66 to 0.74)
Gabon	2.71	1235.19 (1185.99 to 1286.28)	9.09	1862.2 (1823.33 to 1901.77)	1.18 (1.04 to 1.32)
Gambia	2.03	901.93 (860.22 to 945.62)	8.25	1345.03 (1314.81 to 1375.89)	1.02 (0.87 to 1.18)
Georgia	10.82	783.07 (768.3 to 798.07)	10.83	1378.31 (1352 to 1405.06)	2.31 (2.07 to 2.54)
Germany	922.89	4701.63 (4691.93 to 4711.34)	1011.06	5889.06 (5877.41 to 5900.73)	0.65 (0.6 to 0.7)
Ghana	32.7	932.83 (922.32 to 943.45)	127.75	1395.75 (1387.98 to 1403.56)	0.93 (0.69 to 1.17)
Greece	138.88	5508.5 (5479.55 to 5537.57)	149.09	6892.07 (6855.89 to 6928.4)	0.52 (0.35 to 0.69)
Greenland	0.36	2366.43 (2120.02 to 2636.06)	0.4	3141.79 (2841.64 to 3466.47)	0.93 (0.85 to 1.02)
Grenada	0.38	1926.27 (1728.94 to 2143.56)	0.71	2762.43 (2561.51 to 2975.64)	1 (0.87 to 1.13)
Guam	1.5	4247.68 (4030.88 to 4475.11)	2.14	5967.09 (5716.4 to 6226.26)	1.11 (1.02 to 1.2)
Guatemala	49.94	2723.92 (2699.12 to 2748.93)	172.33	3901.16 (3882.45 to 3919.95)	0.96 (0.84 to 1.07)
Guinea	11.07	815.98 (800.52 to 831.7)	40.96	1235.68 (1223.28 to 1248.18)	1.11 (0.97 to 1.24)
Guinea-Bissau	1.88	816.43 (778.38 to 856.13)	6.48	1234.07 (1203.19 to 1265.69)	1.05 (0.79 to 1.31)
Guyana	4.09	1991.94 (1928.65 to 2057.13)	5.88	2883.49 (2809.55 to 2958.98)	1.17 (1.08 to 1.26)
Haiti	23.19	1503.81 (1483.92 to 1523.93)	66.18	1861.43 (1847.17 to 1875.77)	0.83 (0.78 to 0.87)
Honduras	27.67	2619.97 (2587.75 to 2652.56)	112.49	3938.85 (3915.49 to 3962.32)	1.27 (1.16 to 1.38)
Hungary	8.41	332.26 (325.13 to 339.51)	8.74	410.43 (401.55 to 419.47)	0.67 (0.61 to 0.74)
Iceland	3.7	5686.36 (5503.89 to 5873.74)	5.69	7117.2 (6932.74 to 7305.57)	0.74 (0.69 to 0.78)
India	2610.62	1299.09 (1297.49 to 1300.7)	9198.65	2433.4 (2431.82 to 2434.98)	2.35 (2.21 to 2.49)
Indonesia	1377.48	2888.09 (2883.13 to 2893.05)	4160.51	5515.46 (5510.15 to 5520.76)	2.37 (2.28 to 2.46)
Iran (Islamic Republic of)	403.58	3197.01 (3186.62 to 3207.43)	990.12	4238.43 (4229.89 to 4246.98)	1.37 (1.02 to 1.71)
Iraq	137.08	3373.69 (3354.86 to 3392.61)	409.47	3878.8 (3866.8 to 3890.83)	0.61 (0.49 to 0.73)
Ireland	46.61	5309.76 (5261.46 to 5358.42)	76.76	6593.6 (6546.1 to 6641.39)	0.66 (0.57 to 0.75)
Israel	56.54	4647.28 (4608.7 to 4686.13)	133.42	6018.74 (5986.44 to 6051.18)	0.73 (0.65 to 0.81)

Italy	2300.24	16099.08 (16078.24 to 16119.94)	1838.18	15308.01 (15285.15 to 15330.9)	-0.42 (-0.54 to -0.3)
Jamaica	13	2170.74 (2131.84 to 2210.28)	22.66	2920.03 (2882.01 to 2958.45)	0.97 (0.89 to 1.04)
Japan	3663.28	11435.87 (11424 to 11447.75)	3065.76	12262.94 (12248.76 to 12277.14)	0.21 (0.17 to 0.26)
Jordan	26.16	3132.67 (3091.76 to 3174.07)	122.99	3969.62 (3947.22 to 3992.12)	0.87 (0.81 to 0.93)
Kazakhstan	29.14	703.82 (695.62 to 712.09)	47.13	992.02 (983.01 to 1001.09)	1.21 (1.18 to 1.24)
Kenya	58.31	1153.28 (1143.18 to 1163.46)	188.16	1432.55 (1425.86 to 1439.27)	0.53 (0.42 to 0.63)
Kiribati	0.49	2616.83 (2383.66 to 2869.93)	1.24	3855.51 (3641.87 to 4079.51)	1.12 (0.91 to 1.33)
Kuwait	18.02	4286.25 (4220.98 to 4352.53)	75.83	5165.2 (5126.27 to 5204.41)	0.8 (0.75 to 0.85)
Kyrgyzstan	6.8	647.8 (631.72 to 664.28)	13.47	778.16 (765.01 to 791.49)	0.45 (0.37 to 0.52)
Lao People's Democratic Republic	24.11	2518.97 (2486.18 to 2552.13)	92.99	4670.74 (4640.49 to 4701.14)	2.22 (2.16 to 2.28)
Latvia	2.64	404.91 (389.54 to 420.74)	2.09	531.71 (508.39 to 555.93)	1.07 (1 to 1.15)
Lebanon	25.43	3387.33 (3345.2 to 3429.89)	66.3	4453.95 (4419.59 to 4488.53)	0.89 (0.87 to 0.91)
Lesotho	4.5	1197.93 (1162.53 to 1234.22)	9.09	1788.66 (1751.07 to 1826.97)	1.25 (1.12 to 1.37)
Liberia	5.15	933.15 (906.4 to 960.65)	18.94	1364.34 (1344.57 to 1384.37)	1.39 (1.28 to 1.51)
Libya	34.84	3823.45 (3780.2 to 3867.17)	84.1	4230.86 (4202.26 to 4259.62)	0.47 (0.42 to 0.51)
Lithuania	3.41	368.71 (356.41 to 381.32)	2.96	512.44 (493.79 to 531.68)	1.29 (1.22 to 1.37)
Luxembourg	5.3	5386.54 (5241 to 5535.54)	10.82	6894.47 (6762.43 to 7028.75)	0.8 (0.74 to 0.85)
Madagascar	24.57	922.13 (910 to 934.4)	76.59	1065.16 (1057.37 to 1073)	0.53 (0.48 to 0.58)
Malawi	27.86	1239.01 (1223.63 to 1254.57)	68.53	1377.59 (1366.73 to 1388.54)	0.44 (0.32 to 0.56)
Malaysia	206.11	4601.4 (4581.07 to 4621.82)	668.84	7881.36 (7862.41 to 7900.35)	1.88 (1.73 to 2.03)
Maldives	1.28	2726.08 (2567.53 to 2893.27)	7.37	6310.12 (6164.76 to 6458.29)	3.39 (3.11 to 3.66)
Mali	13.24	697.38 (685.17 to 709.78)	60.11	1110.7 (1101.32 to 1120.14)	1.25 (1.05 to 1.46)
Malta	4.82	5067.83 (4924.44 to 5214.56)	6.53	6865.89 (6696.31 to 7039.22)	0.91 (0.77 to 1.04)
Marshall Islands	0.22	2235.09 (1930 to 2588.42)	0.56	3772.02 (3464.05 to 4101.38)	1.47 (1.3 to 1.64)
Mauritania	5.35	1142.32 (1110.61 to 1174.84)	17.22	1609.27 (1584.5 to 1634.38)	0.76 (0.58 to 0.95)
Mauritius	13.55	4496.22 (4419.55 to 4574.06)	22.84	7208.3 (7114.62 to 7302.94)	1.66 (1.55 to 1.78)
Mexico	1474	6740.45 (6729.1 to 6751.81)	2379.83	6793.31 (6784.68 to 6801.95)	-0.53 (-0.76 to -0.3)
Micronesia (Federated States of)	0.66	2867.32 (2642.14 to 3110.39)	1.08	4148.72 (3901.95 to 4407.68)	0.97 (0.77 to 1.16)
Monaco	0.41	5894.02 (5321.95 to 6521.42)	0.49	6942.09 (6328.82 to 7602.48)	0.48 (0.42 to 0.54)
Mongolia	3.14	617.75 (594.89 to 641.4)	7.46	879.25 (859.3 to 899.58)	1.25 (1.2 to 1.29)
Montenegro	0.5	321.3 (293.77 to 350.85)	0.59	412.28 (379.44 to 447.35)	1.03 (0.95 to 1.11)
Morocco	183.65	2913.81 (2900.05 to 2927.63)	375.83	3877.66 (3865.27 to 3890.08)	1.01 (0.97 to 1.05)
Mozambique	28.14	902.65 (891.82 to 913.58)	102.86	1364.6 (1355.82 to 1373.43)	1.33 (1.25 to 1.41)
Myanmar	241.14	2323.85 (2314.24 to 2333.49)	719.36	4768.21 (4757.18 to 4779.26)	2.76 (2.62 to 2.89)
Namibia	4.24	1264.27 (1224.39 to 1305.33)	11.22	1694.01 (1662.41 to 1726.12)	0.91 (0.79 to 1.04)
Nauru	0.08	3331.89 (2632.21 to 4188.63)	0.14	4827.05 (4043.97 to 5732.66)	1.06 (0.99 to 1.12)
Nepal	33.47	740.15 (732.01 to 748.38)	114.52	1254.74 (1247.4 to 1262.12)	1.8 (1.75 to 1.85)
Netherlands	189.83	4758.66 (4737.21 to 4780.19)	220.66	6007.75 (5982.56 to 6033.02)	0.7 (0.65 to 0.74)
New Zealand	96.74	10679.99 (10612.67 to 10747.66)	130.18	10852.23 (10793.12 to 10911.61)	-0.1 (-0.21 to 0.01)
Nicaragua	26.9	2974.67 (2937.11 to 3012.68)	77.23	4237.12 (4207.16 to 4267.24)	1.05 (0.96 to 1.13)
Niger	12.55	734.38 (720.98 to 748.01)	51.99	992.96 (983.81 to 1002.19)	0.9 (0.76 to 1.04)
Nigeria	205.8	1040.15 (1035.36 to 1044.96)	800.54	1420.48 (1417.24 to 1423.73)	0.74 (0.57 to 0.9)
Niue	0.02	3632.59 (2140.84 to 5801.78)	0.02	5514.01 (3407.36 to 8490.12)	1.26 (1.11 to 1.42)
North Macedonia	1.37	268.85 (254.78 to 283.52)	2	373.84 (357.31 to 391.02)	1.16 (1.09 to 1.23)
Northern Mariana Islands	0.57	4056.31 (3716.35 to 4426.35)	0.59	5244.89 (4819.89 to 5699.19)	0.66 (0.51 to 0.81)
Norway	56.73	5356.59 (5312.53 to 5400.94)	74.51	6141.69 (6097.36 to 6186.28)	0.32 (0.22 to 0.42)

Oman	9.45	2780.63 (2722.73 to 2839.64)	47.06	4597.28 (4555.1 to 4639.78)	1.74 (1.65 to 1.82)
Pakistan	293.23	1261.31 (1256.59 to 1266.04)	889.15	1470.5 (1467.4 to 1473.61)	0.54 (0.4 to 0.68)
Palau	0.16	3809.99 (3229.08 to 4477.33)	0.2	5501 (4747.06 to 6349.06)	1.04 (0.88 to 1.19)
Palestine	12.94	2926.59 (2872.88 to 2981.18)	48.48	3711.15 (3677.33 to 3745.23)	0.75 (0.7 to 0.8)
Panama	16.94	2751.51 (2709.14 to 2794.46)	47.08	4398.81 (4359.09 to 4438.81)	1.36 (1.27 to 1.45)
Papua New Guinea	20.46	2122.96 (2092.91 to 2153.39)	82.23	3128.25 (3106.71 to 3149.93)	1 (0.84 to 1.16)
Paraguay	6.82	731.43 (713.66 to 749.58)	21.77	1146.77 (1131.5 to 1162.21)	1.67 (1.61 to 1.73)
Peru	233.54	4304.26 (4286.22 to 4322.37)	593.08	6131.67 (6116.05 to 6147.32)	1.24 (1.2 to 1.27)
Philippines	486.35	3163.57 (3154.38 to 3172.79)	1573.84	5369.83 (5361.38 to 5378.28)	2.16 (2.03 to 2.3)
Poland	46.2	487.59 (483.1 to 492.11)	45.3	512.87 (507.98 to 517.81)	-0.01 (-0.08 to 0.05)
Portugal	115.8	4590.06 (4563.63 to 4616.6)	144.97	6237.22 (6204.07 to 6270.51)	0.74 (0.56 to 0.91)
Puerto Rico	32.29	3368.06 (3331.36 to 3405.07)	32.07	4299.24 (4251.99 to 4346.91)	0.82 (0.74 to 0.9)
Qatar	3.43	4287.23 (4139.69 to 4439.93)	28.43	5071.84 (5009.03 to 5135.35)	0.54 (0.5 to 0.58)
Republic of Korea	492.74	3906.62 (3895.55 to 3917.72)	712	6052.15 (6037.65 to 6066.67)	1.11 (0.78 to 1.43)
Republic of Moldova	3.65	322.36 (311.91 to 333.08)	4.25	473.74 (459.01 to 488.88)	1.52 (1.37 to 1.66)
Romania	15.63	278.33 (273.96 to 282.76)	16.02	396.49 (390.19 to 402.87)	1.24 (1.19 to 1.29)
Russian Federation	152.31	405.09 (403.04 to 407.16)	181.82	525.11 (522.6 to 527.62)	1 (0.96 to 1.04)
Rwanda	17.52	1089.27 (1072.29 to 1106.5)	48.43	1377.66 (1365.14 to 1390.28)	0.81 (0.76 to 0.87)
Saint Kitts and Nevis	0.26	2556.97 (2236.37 to 2922.94)	0.54	3472.73 (3185.46 to 3780.11)	0.9 (0.81 to 0.98)
Saint Lucia	0.73	2123.47 (1964.71 to 2293.66)	1.3	2852.44 (2698.49 to 3013.35)	0.68 (0.54 to 0.81)
Saint Vincent and the Grenadines	0.51	1939.64 (1765.45 to 2129.62)	0.81	2923.6 (2725.42 to 3132.81)	1.37 (1.27 to 1.47)
Samoa	1.26	3434.66 (3237.29 to 3642.46)	2.25	4635.8 (4443 to 4835.35)	0.86 (0.73 to 0.99)
San Marino	0.35	5637.17 (5061.67 to 6261.79)	0.48	6711.16 (6103.52 to 7365.93)	0.52 (0.44 to 0.59)
Sao Tome and Principe	0.25	974.97 (849.87 to 1116.04)	0.81	1447.34 (1347.51 to 1553.31)	1.05 (0.85 to 1.24)
Saudi Arabia	115.26	3522.84 (3501.45 to 3544.37)	507.69	4971.5 (4957.62 to 4985.41)	1.22 (1.2 to 1.24)
Senegal	17.91	1050.98 (1034.83 to 1067.35)	51.6	1325.93 (1314.18 to 1337.77)	0.51 (0.39 to 0.64)
Serbia	6.51	278.62 (271.89 to 285.48)	7.69	378.04 (369.53 to 386.7)	1.11 (1.05 to 1.17)
Seychelles	0.87	4796.91 (4467.51 to 5147.36)	1.63	6741.66 (6416.78 to 7079.12)	1.08 (1.02 to 1.13)
Sierra Leone	8.06	805.93 (787.55 to 824.72)	31.03	1367.43 (1351.55 to 1383.49)	1.53 (1.39 to 1.66)
Singapore	42.25	4458.04 (4415.27 to 4501.16)	107.71	7173.62 (7127.55 to 7219.99)	1.54 (1.4 to 1.68)
Slovakia	3.82	287.45 (278.38 to 296.74)	5.07	400.12 (388.78 to 411.74)	1.07 (1.03 to 1.11)
Slovenia	1.52	304.14 (289.03 to 319.86)	1.8	426.58 (406.37 to 447.63)	1.16 (1.05 to 1.26)
Solomon Islands	1.64	2220.48 (2107.57 to 2338.74)	5.97	3492.35 (3403.36 to 3583.23)	1.23 (0.99 to 1.46)
Somalia	15.36	915.64 (900.87 to 930.62)	51.13	1068.75 (1059.1 to 1078.48)	0.59 (0.55 to 0.64)
South Africa	169.24	1761.02 (1752.35 to 1769.73)	351.65	2255.65 (2248.18 to 2263.14)	0.92 (0.85 to 0.99)
South Sudan	12.99	1011.95 (993.45 to 1030.77)	25.76	1125.02 (1110.88 to 1139.31)	0.18 (0.08 to 0.28)
Spain	476.73	4963.32 (4949.2 to 4977.46)	622.27	6300.98 (6284.64 to 6317.35)	0.66 (0.54 to 0.79)
Sri Lanka	167.58	3644 (3626.36 to 3661.71)	341.2	6058.43 (6038.08 to 6078.84)	1.86 (1.65 to 2.07)
Sudan	91.33	1958.74 (1945.5 to 1972.05)	393.15	3476.85 (3465.74 to 3488)	1.95 (1.8 to 2.1)
Suriname	2.16	2220.67 (2125.65 to 2319.16)	4.39	3029.59 (2940.62 to 3120.6)	0.99 (0.96 to 1.03)
Sweden	84.77	4121.28 (4093.36 to 4149.35)	109.85	4986.01 (4956.27 to 5015.89)	0.49 (0.24 to 0.73)
Switzerland	94	5293.81 (5259.73 to 5328.07)	117.52	5964.56 (5929.76 to 5999.53)	0.41 (0.4 to 0.43)
Syrian Arab Republic	80.31	2915.11 (2893.62 to 2936.75)	148.81	3870.82 (3849.86 to 3891.88)	0.99 (0.91 to 1.07)
Taiwan (Province of China)	163.58	2945.61 (2931.13 to 2960.16)	268.09	4754.69 (4736.04 to 4773.41)	1.78 (1.7 to 1.86)
Tajikistan	6.2	517.26 (503.31 to 531.58)	17.04	665.62 (655.55 to 675.82)	0.89 (0.83 to 0.95)
Thailand	551.22	3437.19 (3427.97 to 3446.44)	1082.5	6661.72 (6648.9 to 6674.55)	2.24 (2.09 to 2.39)

Timor-Leste	4.46	2385.79 (2314.17 to 2459.38)	13.5	3941.3 (3872.02 to 4011.63)	1.96 (1.86 to 2.07)
Togo	7.1	839.15 (818.8 to 859.96)	27.66	1283.78 (1268.51 to 1299.2)	1.09 (0.92 to 1.26)
Tokelau	0.01	3044.19 (1479.16 to 5726.59)	0.02	4987.96 (2843.78 to 8177.16)	1.49 (1.35 to 1.63)
Tonga	0.86	3893.41 (3627.64 to 4175.01)	1.34	5317.64 (5033.54 to 5614.3)	0.69 (0.49 to 0.9)
Trinidad and Tobago	6.92	2237.39 (2184.23 to 2291.66)	11.01	3242.65 (3181.7 to 3304.54)	1.32 (1.18 to 1.46)
Tunisia	57.94	2823.05 (2799.25 to 2847.03)	121.99	3975.86 (3953.37 to 3998.45)	1.21 (1.17 to 1.25)
Turkey	380.7	2663.61 (2654.93 to 2672.32)	807.61	3731.94 (3723.78 to 3740.12)	1.18 (1.13 to 1.24)
Turkmenistan	5.8	654.89 (636.99 to 673.27)	11.99	953.36 (936.34 to 970.62)	1.26 (1.23 to 1.28)
Tuvalu	0.07	2841.94 (2210.98 to 3607.46)	0.13	4501.79 (3755.24 to 5361.45)	1.26 (1.09 to 1.43)
Uganda	39.43	1036.24 (1025.22 to 1047.38)	133.36	1288.04 (1280.73 to 1295.39)	0.72 (0.69 to 0.76)
Ukraine	48.64	380.75 (377.36 to 384.16)	47.34	455.85 (451.57 to 460.17)	0.79 (0.72 to 0.86)
United Arab Emirates	12.25	3525.04 (3458.69 to 3592.74)	79.71	4640.74 (4604.48 to 4677.26)	0.85 (0.73 to 0.96)
United Kingdom	835.3	5848.84 (5836.25 to 5861.45)	1125.56	7309.99 (7296.38 to 7323.62)	0.48 (0.35 to 0.61)
United Republic of Tanzania	71.6	1206.14 (1196.73 to 1215.61)	217.23	1467.47 (1461.09 to 1473.86)	0.63 (0.54 to 0.71)
United States of America	4080.73	6021.6 (6015.73 to 6027.48)	5774.95	7608.41 (7602.19 to 7614.63)	-0.58 (-1.1 to -0.06)
United States Virgin Islands	0.93	3265.08 (3058.04 to 3482.85)	0.7	4124.35 (3821.15 to 4446.57)	0.71 (0.62 to 0.81)
Uruguay	17.18	2294.36 (2260.15 to 2328.95)	31.56	3785.91 (3744.14 to 3828.03)	1.61 (1.42 to 1.8)
Uzbekistan	32.88	671.34 (663.53 to 679.23)	84.08	938.62 (932.26 to 945.01)	1.15 (1.03 to 1.27)
Vanuatu	0.9	2566.23 (2394.65 to 2748.86)	3.01	3834.49 (3696.78 to 3976.41)	1.21 (1.16 to 1.27)
Venezuela (Bolivarian Republic of)	185.09	3792.89 (3775.11 to 3810.75)	325.89	4772.72 (4756.23 to 4789.26)	0.67 (0.61 to 0.74)
Vietnam	371.45	2183.94 (2176.51 to 2191.39)	1124.76	4374.41 (4366.26 to 4382.56)	2.79 (2.62 to 2.97)
Yemen	55.29	2021.2 (2003.6 to 2038.94)	208.74	2500.22 (2489.24 to 2511.25)	0.99 (0.87 to 1.11)
Zambia	25.29	1388.55 (1370.09 to 1407.23)	83.19	1691.06 (1679.04 to 1703.16)	0.51 (0.46 to 0.56)
Zimbabwe	34.72	1458.6 (1442.24 to 1475.13)	63.88	1575.4 (1562.91 to 1587.97)	-0.12 (-0.3 to 0.06)

S14 Table. The global prevalence of female infertility among women aged 15-49 years in 1990 and 2021, along with the trends and changes observed between these years, by country and territories

Countries	Number of cases (Thousands) in 1990	Age-standardized rate per 100,000 population (95% UI) in 2021	Number of cases (Thousands) in 2021	Age-standardized rate per 100,000 population (95% UI) in 2021	Estimated annual percentage changes (95% CI) from 1990 to 2021
Afghanistan	52.22	2808.85 (2783.48 to 2834.43)	103.77	1599.12 (1588.87 to 1609.41)	-2.52 (-2.95 to -2.09)
Albania	26.29	3121.53 (3083.12 to 3160.37)	12.16	1986.31 (1951.12 to 2022)	-1.48 (-2.57 to -0.38)
Algeria	97.71	1758.57 (1747 to 1770.22)	411.46	3483.32 (3472.65 to 3494.02)	2.9 (2.55 to 3.24)
American Samoa	0.48	4324.82 (3935.53 to 4747.36)	0.45	4121.4 (3747.6 to 4523.56)	-0.11 (-0.14 to -0.09)
Andorra	0.37	2327.92 (2095 to 2581.85)	0.5	2180.98 (1988.04 to 2391.2)	-0.22 (-0.29 to -0.16)
Angola	150.74	7354.02 (7316.02 to 7392.17)	533.94	7809.53 (7788.3 to 7830.81)	0.25 (0.22 to 0.28)
Antigua and Barbuda	0.95	5866.94 (5496.97 to 6258.58)	1.45	5890.75 (5590.61 to 6203.8)	0.05 (0.03 to 0.07)
Argentina	185.37	2378.91 (2368.09 to 2389.77)	261.64	2158.31 (2150.04 to 2166.6)	-0.29 (-0.37 to -0.2)
Armenia	32.53	3374.11 (3337.08 to 3411.55)	7.69	1000.27 (977.59 to 1023.42)	-2.71 (-3.31 to -2.11)
Australia	12.71	274.23 (269.48 to 279.05)	16.21	252.41 (248.53 to 256.34)	-0.19 (-0.36 to -0.02)
Austria	110.56	5665.51 (5632.07 to 5699.11)	126.85	5982.28 (5949.29 to 6015.43)	1.05 (0.74 to 1.36)
Azerbaijan	88.76	4368.39 (4338.75 to 4398.24)	135.5	4536.64 (4512.34 to 4561.06)	-0.39 (-1.65 to 0.88)
Bahamas	3.98	5633.06 (5456.54 to 5814.48)	6.01	5665.83 (5523.38 to 5811.06)	0.06 (0.05 to 0.08)
Bahrain	4.26	3759.26 (3641.68 to 3880.61)	12.86	3818.21 (3752.42 to 3884.9)	0.06 (0.03 to 0.09)
Bangladesh	435.32	1560.58 (1555.78 to 1565.39)	1506.09	3080.22 (3075.29 to 3085.16)	2.95 (2.56 to 3.34)
Barbados	4.13	5873.28 (5694.58 to 6056.74)	4.15	5818.22 (5641.88 to 5998.93)	0.02 (0 to 0.04)
Belarus	134.61	4984.61 (4957.94 to 5011.4)	115.51	5215.22 (5184.37 to 5246.23)	0.26 (0.22 to 0.3)
Belgium	67.21	2565.24 (2545.87 to 2584.74)	135.22	5133.69 (5106.25 to 5161.24)	2.42 (1.9 to 2.96)
Belize	0.75	1863.13 (1727.45 to 2008.98)	3.32	2860.93 (2763.94 to 2960.61)	1.67 (1.22 to 2.11)
Benin	31.77	3154.32 (3118.45 to 3190.55)	43.77	1400.88 (1387.45 to 1414.44)	-1.91 (-2.18 to -1.63)
Bermuda	1.08	5792.99 (5451.43 to 6153.21)	0.82	5846.59 (5446.27 to 6271.88)	0.07 (0.05 to 0.08)
Bhutan	3.99	2781.36 (2692.46 to 2873.04)	6.05	2786.34 (2716.42 to 2857.79)	-0.02 (-0.04 to 0)
Bolivia (Plurinational State of)	4.04	316.06 (306.33 to 326.04)	24.34	793.25 (783.29 to 803.31)	6.68 (3.88 to 9.56)
Bosnia and Herzegovina	45.69	3804.88 (3770.03 to 3840)	28.24	3900.07 (3854.33 to 3946.26)	0.17 (0.13 to 0.2)
Botswana	6.6	2246.23 (2190.75 to 2302.97)	32.63	4601.27 (4551.43 to 4651.58)	0.78 (0.26 to 1.3)
Brazil	1019.8	2736.1 (2730.74 to 2741.47)	2125.45	3435.04 (3430.42 to 3439.68)	1.71 (1.1 to 2.33)
Brunei Darussalam	0.46	820.38 (745.37 to 902.01)	0.95	718.16 (673.26 to 765.51)	-0.45 (-0.72 to -0.18)
Bulgaria	80.03	3908.5 (3881.33 to 3935.81)	56.19	3944 (3910.75 to 3977.49)	0.03 (0.01 to 0.06)
Burkina Faso	24.83	1219.82 (1204.22 to 1235.6)	151.36	3013.48 (2997.94 to 3029.08)	2.16 (1.29 to 3.03)
Burundi	26.43	2437.35 (2407.23 to 2467.8)	20.73	699.16 (689.5 to 708.95)	-4.49 (-5.64 to -3.33)
Cabo Verde	3.54	5137.71 (4959.51 to 5321.52)	8.53	5647.64 (5527.87 to 5769.58)	0.46 (0.41 to 0.51)
Cambodia	110.43	4827.45 (4798.6 to 4856.46)	220.33	4826.84 (4806.64 to 4847.11)	-2.48 (-3.43 to -1.53)
Cameroon	103.17	5076.95 (5045.08 to 5108.98)	549.48	7462.65 (7442.55 to 7482.8)	0.3 (-0.89 to 1.51)
Canada	82.46	1035.85 (1028.77 to 1042.96)	81.97	961.17 (954.58 to 967.8)	-0.19 (-0.24 to -0.14)
Central African Republic	54.15	9609.22 (9526.16 to 9692.88)	148.17	11739.3 (11678.7 to 11800.17)	0.7 (0.47 to 0.93)
Chad	42.01	3469.48 (3435.58 to 3503.67)	143.99	4311.76 (4288.84 to 4334.78)	-4.27 (-5.56 to -2.97)
Chile	79.75	2312.52 (2296.38 to 2328.76)	106.5	2199.58 (2186.38 to 2212.84)	-0.16 (-0.28 to -0.04)
China	24796.28	8155.29 (8152.04 to 8158.53)	29317	8505.94 (8502.83 to 8509.06)	0.01 (-0.03 to 0.05)
Colombia	27.19	344.35 (340.21 to 348.53)	58.27	430.51 (427.02 to 434.03)	1.18 (0.56 to 1.8)
Comoros	8.91	9254 (9057.12 to 9454.56)	17.95	9483.17 (9344.07 to 9623.91)	1 (0.45 to 1.55)
Congo	28.22	5961.38 (5889.88 to 6033.62)	87.06	6362.51 (6320.23 to 6405.02)	-0.52 (-1.27 to 0.25)
Cook Islands	0.19	4512.37 (3876.9 to 5227.88)	0.18	4305.83 (3694.3 to 4992.89)	-0.14 (-0.16 to -0.12)

Costa Rica	28.17	3729.38 (3685.29 to 3773.95)	49.84	3664.43 (3632.32 to 3696.77)	-0.06 (-0.08 to -0.04)
Côte d'Ivoire	62.1	2562.91 (2541.78 to 2584.2)	405.92	6387.73 (6367.85 to 6407.67)	2.6 (1.87 to 3.34)
Croatia	50.25	4039.1 (4003.81 to 4074.63)	35.64	3957.41 (3916.11 to 3999.08)	-0.15 (-0.24 to -0.06)
Cuba	179.44	6193.45 (6164.42 to 6222.59)	144.32	5908.44 (5877.96 to 5939.06)	-0.06 (-0.14 to 0.01)
Cyprus	4.92	2458.73 (2390.44 to 2528.5)	9.76	2270.18 (2224.87 to 2316.44)	-0.48 (-0.66 to -0.31)
Czechia	141.91	5657.6 (5627.9 to 5687.42)	128.04	5746.08 (5713.96 to 5778.36)	1.45 (0.91 to 1.99)
Democratic People's Republic of Korea	313.73	6157.2 (6135.44 to 6179.02)	410.66	6228.52 (6209.47 to 6247.61)	-0.05 (-0.08 to -0.02)
Democratic Republic of the Congo	333.01	4468.76 (4453.2 to 4484.36)	862.74	4581.47 (4571.65 to 4591.32)	-0.81 (-2.36 to 0.77)
Denmark	3.21	232.58 (224.56 to 240.83)	8.21	651.33 (637.26 to 665.65)	3.64 (2.51 to 4.78)
Djibouti	9.44	10671.54 (10450.92 to 10896.34)	36.06	10812.69 (10701.3 to 10925.04)	0.39 (-0.18 to 0.95)
Dominica	0.88	5732.41 (5353.44 to 6132.81)	0.93	5721.8 (5359.51 to 6102.58)	0.02 (0.01 to 0.03)
Dominican Republic	33.42	1876.16 (1855.56 to 1896.97)	139.68	4872.3 (4846.72 to 4897.99)	1.99 (0.75 to 3.24)
Ecuador	3.74	159.58 (154.4 to 164.91)	34.63	748.39 (740.51 to 756.33)	9.35 (7.3 to 11.44)
Egypt	258.43	2065.57 (2057.5 to 2073.66)	989.41	3825.1 (3817.55 to 3832.67)	1.35 (0.72 to 1.99)
El Salvador	11.02	956.34 (938.21 to 974.77)	26.37	1536.14 (1517.56 to 1554.91)	5.15 (2.93 to 7.41)
Equatorial Guinea	6.56	7531.93 (7346.89 to 7720.78)	26.89	8003.94 (7907.25 to 8101.67)	0.28 (0.22 to 0.33)
Eritrea	39.35	5177.22 (5124.79 to 5230.12)	142.8	9003.58 (8956.45 to 9050.91)	2.31 (2.05 to 2.56)
Estonia	18.69	4777.84 (4709.42 to 4847.06)	15.14	5313.82 (5228.12 to 5400.71)	0.34 (0.29 to 0.39)
Eswatini	4.4	2520.1 (2444.01 to 2598.29)	8	2546.38 (2490.35 to 2603.56)	-0.65 (-2.4 to 1.14)
Ethiopia	625.16	5893.51 (5878.48 to 5908.57)	992.15	3876.7 (3868.91 to 3884.49)	-0.57 (-0.88 to -0.25)
Fiji	12.64	6800.7 (6681.43 to 6921.76)	15.29	6682.32 (6576.78 to 6789.19)	-0.06 (-0.08 to -0.04)
Finland	7.56	504.2 (492.79 to 515.86)	21.05	1709.11 (1686.03 to 1732.46)	1.3 (0.52 to 2.09)
France	361.69	2374.48 (2366.73 to 2382.24)	625.85	4295.8 (4285.1 to 4306.52)	2.06 (1.47 to 2.65)
Gabon	19.31	10227.74 (10076.37 to 10381.11)	46.82	10372.65 (10277.84 to 10468.16)	0.26 (-0.21 to 0.74)
Gambia	9.72	4748.09 (4649.71 to 4848.46)	28.49	5070.88 (5010.73 to 5131.68)	-0.56 (-1 to -0.13)
Georgia	51.07	3552.35 (3521.49 to 3583.43)	26.63	3280.29 (3240.61 to 3320.39)	-0.34 (-0.44 to -0.25)
Germany	60.65	305.86 (303.43 to 308.31)	220.85	1159.97 (1155.12 to 1164.84)	4.7 (3.32 to 6.09)
Ghana	54.58	1656.33 (1642.05 to 1670.72)	377.92	4291.49 (4277.69 to 4305.32)	0.38 (-0.51 to 1.27)
Greece	59.09	2337.08 (2318.26 to 2356.02)	51.81	2106.54 (2087.98 to 2125.25)	-0.39 (-0.51 to -0.26)
Greenland	0.16	982.67 (833.22 to 1154.55)	0.13	968.19 (809.55 to 1150.96)	-0.14 (-0.19 to -0.09)
Grenada	1.06	5800.65 (5449.08 to 6172.19)	1.44	5856.18 (5555.65 to 6169.26)	0.03 (0.01 to 0.05)
Guam	1.52	4322.65 (4106.04 to 4549.77)	1.4	4040.03 (3830.84 to 4257.91)	-0.28 (-0.31 to -0.25)
Guatemala	31.47	2096.28 (2072.94 to 2119.85)	133.61	3255.56 (3237.98 to 3273.21)	-1.3 (-2.48 to -0.11)
Guinea	71.49	5483.93 (5443.31 to 5524.81)	136.37	4195.3 (4172.64 to 4218.09)	-0.22 (-0.53 to 0.09)
Guinea-Bissau	7.72	3577.14 (3495.65 to 3660.31)	21.95	4413.64 (4354.48 to 4473.54)	1.02 (0.91 to 1.13)
Guyana	15.38	8046.58 (7916.92 to 8178.15)	15.89	8103.23 (7976.38 to 8231.66)	-0.43 (-1.48 to 0.63)
Haiti	55.67	3891.76 (3858.92 to 3924.85)	113.71	3183.79 (3165.29 to 3202.39)	-0.17 (-0.39 to 0.04)
Honduras	32.56	3482.99 (3444.54 to 3521.8)	95.11	3538.22 (3515.58 to 3560.98)	0.53 (-1.58 to 2.68)
Hungary	96.76	3790.17 (3766.09 to 3814.38)	80.01	3915.7 (3888.21 to 3943.36)	0.01 (-0.02 to 0.05)
Iceland	1.52	2362.88 (2245.55 to 2485.15)	1.96	2361.61 (2257.94 to 2469.1)	-0.12 (-0.24 to 0.01)
India	8702.43	4443.82 (4440.83 to 4446.81)	29075.29	7636.42 (7633.63 to 7639.2)	2.53 (1.85 to 3.21)
Indonesia	2407.86	5450.99 (5443.99 to 5458)	6251.54	8241.48 (8235.02 to 8247.95)	1.67 (1.1 to 2.25)
Iran (Islamic Republic of)	419.26	3406.81 (3396.16 to 3417.48)	815.74	3319.58 (3312.21 to 3326.96)	-0.78 (-1.65 to 0.11)
Iraq	137.11	3728.15 (3707.79 to 3748.62)	385.45	3837.7 (3825.5 to 3849.94)	0.12 (0.1 to 0.14)
Ireland	20.51	2375.31 (2342.9 to 2408.08)	30.33	2327.82 (2301.19 to 2354.71)	-0.05 (-0.14 to 0.04)
Israel	19.07	1542.72 (1520.89 to 1564.83)	39.82	1791.2 (1773.63 to 1808.92)	0.29 (0.07 to 0.5)

Italy	163.8	1158.79 (1153.17 to 1164.43)	281.02	2235.98 (2227.59 to 2244.39)	2.77 (2.32 to 3.21)
Jamaica	38.28	6927.07 (6855.84 to 6998.96)	54.43	6953.11 (6894.66 to 7011.95)	0.03 (0.01 to 0.06)
Japan	558.71	1523.24 (1519.18 to 1527.3)	424.42	1486.87 (1482.34 to 1491.41)	-0.36 (-0.65 to -0.06)
Jordan	10.48	1309.57 (1282.81 to 1336.82)	99.62	3376.58 (3355.54 to 3397.73)	2.37 (1.97 to 2.76)
Kazakhstan	90.99	2058.82 (2045.37 to 2072.34)	175.04	3535.07 (3518.42 to 3551.78)	3.29 (2.57 to 4.02)
Kenya	139.19	3183.83 (3166.39 to 3201.35)	617.52	5017.71 (5005 to 5030.44)	-3.89 (-5.6 to -2.15)
Kiribati	0.8	4579.27 (4261.26 to 4917.61)	1.39	4430.49 (4199.81 to 4671.59)	-0.05 (-0.08 to -0.02)
Kuwait	15.77	3778.26 (3718.01 to 3839.5)	62.23	3684.82 (3654.79 to 3715.1)	-0.04 (-0.08 to 0)
Kyrgyzstan	49.17	4645.91 (4603.94 to 4688.26)	85.51	4838.45 (4805.94 to 4871.14)	0.63 (0.01 to 1.26)
Lao People's Democratic Republic	37.4	4359.48 (4314.69 to 4404.66)	78.11	4030.18 (4001.82 to 4058.7)	-0.19 (-0.21 to -0.17)
Latvia	19.5	2972.17 (2930.55 to 3014.26)	21.56	5446.72 (5372.89 to 5521.44)	2.71 (2.23 to 3.2)
Lebanon	38.27	5338.63 (5284.64 to 5393.05)	86.05	5482.26 (5445.29 to 5519.44)	1.95 (1.33 to 2.58)
Lesotho	21.19	6041.13 (5959.44 to 6123.73)	28.6	5928.36 (5858.98 to 5998.47)	-3.88 (-5.56 to -2.17)
Liberia	23.75	4861.57 (4797.35 to 4926.57)	87.23	6692.68 (6647.95 to 6737.67)	0.08 (-0.51 to 0.66)
Libya	15.92	1907.66 (1876.38 to 1939.41)	69.08	3376.2 (3351.01 to 3401.55)	2.43 (1.97 to 2.89)
Lithuania	30	3239.65 (3203.05 to 3276.58)	32.23	5593.85 (5532.72 to 5655.56)	2.37 (1.93 to 2.82)
Luxembourg	2.24	2140.79 (2052.96 to 2231.94)	3.59	2053.28 (1986.36 to 2122.29)	-0.03 (-0.22 to 0.16)
Madagascar	86.37	3527.1 (3502.9 to 3551.44)	355.63	5263.06 (5245.43 to 5280.73)	1.26 (0.39 to 2.13)
Malawi	128.75	6440.56 (6404.22 to 6477.08)	41.57	777.46 (769.88 to 785.11)	-6.3 (-6.74 to -5.86)
Malaysia	220.38	5182.24 (5160.44 to 5204.12)	407.01	4778.75 (4764.06 to 4793.49)	-0.27 (-0.3 to -0.23)
Maldives	3.99	9965.64 (9643 to 10297.38)	9.19	7081.7 (6936.79 to 7229.23)	-1.7 (-2.26 to -1.14)
Mali	38.35	2063.18 (2042.02 to 2084.53)	109.18	1865.68 (1854.28 to 1877.16)	0.67 (-0.37 to 1.72)
Malta	2.58	2453.16 (2358.62 to 2550.84)	2.52	2358.03 (2266.22 to 2453.32)	-0.23 (-0.45 to -0.01)
Marshall Islands	0.4	4565.71 (4118.78 to 5060.48)	0.63	4397.97 (4061.91 to 4755.61)	-0.12 (-0.14 to -0.1)
Mauritania	7.68	1711.53 (1672.26 to 1751.63)	58.06	5883.49 (5834.83 to 5932.49)	4.38 (3.88 to 4.89)
Mauritius	12.66	4229.54 (4155.59 to 4304.66)	12.59	3922.62 (3853.96 to 3992.23)	-0.24 (-0.26 to -0.22)
Mexico	312.14	1740.69 (1734.5 to 1746.9)	1614.55	4648.86 (4641.69 to 4656.04)	1.48 (0.83 to 2.14)
Micronesia (Federated States of)	0.9	4414.55 (4125.43 to 4721.99)	1.04	4358.67 (4096.23 to 4633.86)	-0.01 (-0.03 to 0.01)
Monaco	0.18	2283.16 (1955.47 to 2663.91)	0.17	2202.63 (1879.98 to 2570.54)	-0.11 (-0.17 to -0.06)
Mongolia	15.84	3199.67 (3147.8 to 3252.31)	31.16	3522.23 (3483.1 to 3561.72)	0.34 (0.29 to 0.4)
Montenegro	6.1	3847.9 (3751.88 to 3945.88)	5.38	3775.75 (3674.96 to 3878.74)	-0.01 (-0.02 to 0.01)
Morocco	123.56	2206.96 (2194.45 to 2219.54)	430.24	4406.7 (4393.54 to 4419.89)	3.83 (2.64 to 5.02)
Mozambique	233.41	7936.91 (7904.34 to 7969.6)	574.16	8479.16 (8456.55 to 8501.82)	-1.15 (-1.94 to -0.36)
Myanmar	435.81	4531.95 (4518.25 to 4545.69)	296.75	2050.49 (2043.11 to 2057.89)	-3.5 (-4.1 to -2.9)
Namibia	8.02	2927.41 (2862.21 to 2993.86)	19.81	3066.49 (3023.65 to 3109.84)	-1.97 (-4.37 to 0.5)
Nauru	0.1	4571.34 (3729.57 to 5571.48)	0.12	4435.51 (3670.78 to 5325.89)	-0.12 (-0.14 to -0.09)
Nepal	159.02	3395.62 (3378.66 to 3412.66)	150.43	1513.89 (1506.21 to 1521.61)	-0.52 (-1.87 to 0.85)
Netherlands	99.45	2386.5 (2371.66 to 2401.41)	88.18	2368.81 (2353.18 to 2384.53)	0.03 (-0.02 to 0.08)
New Zealand	2.26	246.42 (236.35 to 256.83)	7.73	632.02 (618 to 646.3)	4.33 (3.51 to 5.17)
Nicaragua	14.62	1839.07 (1808.63 to 1869.97)	32.87	1821.69 (1802.02 to 1841.55)	3.95 (1.99 to 5.94)
Niger	80.5	5587.22 (5547.75 to 5626.92)	231.24	5129.85 (5108.09 to 5151.69)	-0.44 (-1.15 to 0.28)
Nigeria	744.78	3866.07 (3856.83 to 3875.33)	1892.83	3697.61 (3692.22 to 3703)	-1.12 (-1.54 to -0.71)
Niue	0.02	4566.38 (2822.3 to 7004.19)	0.02	4375.25 (2513.43 to 7123.18)	-0.15 (-0.16 to -0.13)
North Macedonia	19.85	3892.05 (3838.07 to 3946.61)	21.44	3886.08 (3833.88 to 3938.9)	0.07 (0.05 to 0.09)
Northern Mariana Islands	0.61	4394.55 (4043.31 to 4775.57)	0.43	4294.64 (3897.82 to 4722.06)	-0.05 (-0.08 to -0.02)
Norway	39.29	3619.91 (3584.12 to 3655.97)	46.12	3699.21 (3665.48 to 3733.19)	-0.13 (-0.27 to 0.01)

Oman	13.06	4119.59 (4047.49 to 4192.83)	43.44	3941.33 (3904.01 to 3978.95)	-0.19 (-0.21 to -0.17)
Pakistan	1813.53	8209.41 (8197.23 to 8221.61)	4817.4	7858.79 (7851.72 to 7865.86)	-5.63 (-10.1 to -0.93)
Palau	0.18	4555.76 (3910.32 to 5288.47)	0.16	4352.44 (3689.6 to 5109.03)	-0.09 (-0.12 to -0.07)
Palestine	15.17	3897.16 (3832.13 to 3963.13)	48.57	3981.8 (3945.8 to 4018.07)	0.07 (0.05 to 0.09)
Panama	25.4	4488.63 (4432.86 to 4544.99)	45.75	4383 (4342.87 to 4423.42)	-0.06 (-0.09 to -0.04)
Papua New Guinea	20.95	2356.03 (2323.57 to 2388.88)	37.49	1381.07 (1367.06 to 1395.21)	-2.01 (-2.56 to -1.46)
Paraguay	22.65	2805.55 (2768.53 to 2842.97)	90.28	4860.5 (4828.77 to 4892.41)	1.95 (1.43 to 2.46)
Peru	3.86	81.68 (79.08 to 84.36)	121.87	1245.75 (1238.76 to 1252.77)	7.08 (5.47 to 8.71)
Philippines	1129.72	7803.74 (7789.15 to 7818.36)	2499.07	8735.29 (8724.41 to 8746.17)	6.56 (3.8 to 9.4)
Poland	463.16	4711.48 (4697.79 to 4725.21)	657.23	7116.36 (7098.79 to 7133.96)	1.41 (0.99 to 1.83)
Portugal	59.05	2358.64 (2339.64 to 2377.76)	55.23	2149.31 (2130.98 to 2167.78)	-0.24 (-0.31 to -0.18)
Puerto Rico	54.88	5822.15 (5773.5 to 5871.12)	42.73	5722.98 (5668.62 to 5777.76)	-0.05 (-0.06 to -0.03)
Qatar	3.18	3804.62 (3671.5 to 3942.7)	24.66	3849.66 (3799.48 to 3900.48)	0.1 (0.07 to 0.12)
Republic of Korea	91.67	843.05 (837.56 to 848.57)	112.15	809.3 (804.52 to 814.12)	-0.14 (-0.32 to 0.05)
Republic of Moldova	63.16	5260.11 (5218.93 to 5301.55)	52.44	5586.91 (5537.99 to 5636.23)	1.44 (0.36 to 2.53)
Romania	205.73	3671.56 (3655.62 to 3687.57)	150.45	3855.73 (3835.96 to 3875.58)	0.25 (0.15 to 0.35)
Russian Federation	2933.15	7252.23 (7243.89 to 7260.58)	2715.41	7763.54 (7754 to 7773.1)	0.26 (0.25 to 0.28)
Rwanda	7.75	480.91 (469.94 to 492.11)	46.5	1360.13 (1347.69 to 1372.67)	-0.58 (-1.88 to 0.75)
Saint Kitts and Nevis	0.57	5818.29 (5333.57 to 6347.08)	0.95	5931.38 (5558.88 to 6323.58)	0.04 (0.01 to 0.06)
Saint Lucia	1.78	5689.43 (5420.92 to 5969.57)	2.73	5893.66 (5673.91 to 6120.25)	0.11 (0.09 to 0.13)
Saint Vincent and the Grenadines	1.39	5813.81 (5502 to 6141.55)	1.61	5841.86 (5559.15 to 6135.61)	-0.03 (-0.05 to -0.01)
Samoa	1.4	4507.46 (4268.01 to 4757.97)	1.94	4403 (4207.95 to 4605.13)	-0.07 (-0.08 to -0.05)
San Marino	0.14	2306.04 (1939.89 to 2722.52)	0.17	2241.78 (1907.33 to 2622.36)	-0.11 (-0.17 to -0.05)
Sao Tome and Principe	0.46	2064.28 (1873.42 to 2271.52)	1.16	2194.71 (2068.97 to 2326.69)	-0.05 (-1.27 to 1.17)
Saudi Arabia	117.02	3927.63 (3904.47 to 3950.93)	432.94	3896.57 (3884.89 to 3908.28)	-0.01 (-0.04 to 0.01)
Senegal	39.82	2656.08 (2629.22 to 2683.19)	117.79	3244.73 (3225.9 to 3263.65)	1.69 (0.9 to 2.49)
Serbia	94.6	4006.47 (3980.97 to 4032.09)	78.59	3875.87 (3848.64 to 3903.25)	-0.1 (-0.16 to -0.04)
Seychelles	0.68	4259.63 (3934.78 to 4606.69)	0.94	3848.21 (3605.53 to 4103.39)	-0.26 (-0.28 to -0.23)
Sierra Leone	54.99	5862.17 (5811.72 to 5913.03)	137.49	6654.11 (6618.11 to 6690.3)	-0.61 (-1.23 to 0.02)
Singapore	7.22	797.09 (778.76 to 815.77)	18.3	883.43 (870.45 to 896.66)	0.08 (-0.2 to 0.36)
Slovakia	55.1	4025.03 (3991.42 to 4058.87)	50.68	3902.27 (3867.8 to 3937.02)	-0.24 (-0.33 to -0.15)
Slovenia	4.79	932.21 (905.97 to 959.06)	11.5	2707.23 (2657.12 to 2758.15)	4.84 (3.91 to 5.77)
Solomon Islands	2.69	4297.12 (4131.09 to 4468.79)	6.87	4266.43 (4165.68 to 4369.12)	0.01 (-0.03 to 0.05)
Somalia	75.36	4661.3 (4627.84 to 4694.97)	214.54	4892.27 (4871.18 to 4913.43)	0.16 (0.12 to 0.19)
South Africa	603.96	6572.31 (6555.46 to 6589.2)	564.55	3447.94 (3438.94 to 3456.96)	-0.7 (-1.58 to 0.2)
South Sudan	51.48	4378.54 (4339.36 to 4418.04)	100.3	4760.29 (4730.52 to 4790.21)	0.28 (0.25 to 0.31)
Spain	38.79	423.35 (419.14 to 427.59)	136.11	1179.27 (1172.82 to 1185.74)	4.9 (3.87 to 5.93)
Sri Lanka	76.4	1673.98 (1662.07 to 1685.97)	183.59	3225.73 (3210.94 to 3240.57)	0.47 (-0.06 to 1)
Sudan	207.09	5003.7 (4981.58 to 5025.9)	757.02	7030.45 (7014.44 to 7046.5)	1.75 (1.23 to 2.27)
Suriname	5.25	5863.33 (5702.83 to 6027.44)	8.4	5834.54 (5710.41 to 5960.7)	-0.03 (-0.04 to -0.01)
Sweden	91.07	4315.97 (4287.78 to 4344.32)	100.56	4375.09 (4348.01 to 4402.31)	-0.02 (-0.06 to 0.01)
Switzerland	39.79	2137.7 (2116.71 to 2158.88)	49.3	2246.9 (2226.99 to 2266.98)	0.19 (0.05 to 0.33)
Syrian Arab Republic	50.36	2098.64 (2079.54 to 2117.89)	131.62	3545.82 (3525.74 to 3566)	2.55 (1.98 to 3.12)
Taiwan (Province of China)	345.23	6109.74 (6089.2 to 6130.34)	369.47	5986.81 (5967.19 to 6006.49)	-0.06 (-0.11 to -0.02)
Tajikistan	31.06	2608.51 (2578.15 to 2639.25)	33.51	1194.26 (1181.48 to 1207.16)	-2.84 (-3.31 to -2.36)
Thailand	278.9	1726.57 (1720.11 to 1733.06)	571.87	3444.26 (3435.23 to 3453.31)	0.65 (0.14 to 1.17)

Timor-Leste	5.69	3248.3 (3162.87 to 3335.73)	8.94	3082.54 (3017.4 to 3148.8)	-0.42 (-1.01 to 0.16)
Togo	8.22	958.14 (936.76 to 979.97)	80.31	3846.79 (3820.08 to 3873.65)	2.67 (1.74 to 3.6)
Tokelau	0.01	4493.29 (2465.07 to 7647.26)	0.01	4306.9 (2311.4 to 7370.87)	-0.11 (-0.13 to -0.1)
Tonga	0.85	4500.34 (4197.51 to 4820.1)	1.01	4318.68 (4055.5 to 4594.91)	-0.1 (-0.11 to -0.08)
Trinidad and Tobago	13.5	4439.25 (4364.06 to 4515.53)	26.03	7179.3 (7091.45 to 7268.04)	0.6 (0.27 to 0.92)
Tunisia	39.28	1981.82 (1961.65 to 2002.18)	119.05	3659.91 (3638.96 to 3680.95)	2.31 (2.03 to 2.59)
Turkey	183.54	1230.94 (1225.2 to 1236.69)	811.79	3667.26 (3659.26 to 3675.27)	0.7 (-0.36 to 1.78)
Turkmenistan	31.42	3507.62 (3467.43 to 3548.26)	46.46	3707.43 (3673.74 to 3741.36)	0.28 (0.24 to 0.33)
Tuvalu	0.11	4545.38 (3734.76 to 5489.49)	0.12	4403.5 (3643.46 to 5280.8)	-0.08 (-0.1 to -0.07)
Uganda	132.89	4046.35 (4023.54 to 4069.28)	108	1016.96 (1010.78 to 1023.18)	-4.97 (-5.33 to -4.6)
Ukraine	809.76	6188.51 (6175.01 to 6202.04)	688.46	6695.67 (6679.37 to 6712)	1.91 (1.29 to 2.53)
United Arab Emirates	14.25	4085.86 (4016.77 to 4156.26)	87.7	3966.83 (3936.47 to 3997.41)	-0.08 (-0.1 to -0.07)
United Kingdom	471.76	3243.77 (3234.48 to 3253.08)	455.08	2812.45 (2804.27 to 2820.65)	-0.08 (-0.27 to 0.1)
United Republic of Tanzania	315.71	6390.03 (6366.95 to 6413.18)	501.89	3668.19 (3657.87 to 3678.53)	-2.19 (-2.34 to -2.03)
United States of America	756.31	1050.87 (1048.5 to 1053.24)	1396.8	1820.47 (1817.45 to 1823.49)	3.3 (1.73 to 4.9)
United States Virgin Islands	1.53	5400.51 (5131.73 to 5680.07)	0.95	5535.71 (5186.36 to 5903.86)	0.14 (0.12 to 0.16)
Uruguay	16.91	2297.4 (2262.89 to 2332.31)	18.95	2237.2 (2205.38 to 2269.37)	-0.14 (-0.24 to -0.05)
Uzbekistan	125.08	2563.12 (2548.2 to 2578.13)	256.63	2748.25 (2737.61 to 2758.93)	2.09 (1.38 to 2.79)
Vanuatu	1.44	4536.68 (4300.28 to 4784.45)	3.16	4313.59 (4163.45 to 4468.07)	-0.15 (-0.17 to -0.13)
Venezuela (Bolivarian Republic of)	137.42	2975.81 (2959.91 to 2991.79)	213.84	3022.32 (3009.44 to 3035.24)	0.14 (0.08 to 0.19)
Vietnam	309.76	1993.92 (1986.63 to 2001.24)	449.46	1623.87 (1619.12 to 1628.64)	2.08 (1.17 to 3)
Yemen	110.26	4195.49 (4169.98 to 4221.13)	428.5	5302.88 (5286.87 to 5318.93)	0.4 (-0.38 to 1.18)
Zambia	74.55	4853.46 (4817.07 to 4890.08)	187.2	4144.37 (4125.17 to 4163.65)	-3.3 (-4.59 to -1.99)
Zimbabwe	47.6	2343.45 (2321.85 to 2365.23)	128.92	3362.07 (3343.59 to 3380.63)	-1.55 (-2.54 to -0.55)

S15 Table. The global prevalence of endometriosis among women aged 15-49 years in 1990 and 2021, along with the trends and changes observed between these years, by country and territories

Countries	Number of cases (Thousands) in 1990	Age-standardized rate per 100,000 population (95% UI) in 2021	Number of cases (Thousands) in 2021	Age-standardized rate per 100,000 population (95% UI) in 2021	Estimated annual percentage changes (95% CI) from 1990 to 2021
Afghanistan	49.4	2563.07 (2538.99 to 2587.35)	134.07	2007.35 (1996.06 to 2018.71)	-0.79 (-1 to -0.59)
Albania	10.84	1294.38 (1269.3 to 1319.89)	6.28	1001.86 (977.16 to 1027.05)	-0.86 (-1 to -0.71)
Algeria	100.35	1803.81 (1792.05 to 1815.63)	150.4	1313.64 (1306.98 to 1320.32)	-0.79 (-0.92 to -0.66)
American Samoa	0.26	2294.65 (2015.26 to 2607.42)	0.18	1583.94 (1358.15 to 1838.03)	-1.2 (-1.23 to -1.18)
Andorra	0.12	814.13 (676.42 to 973.7)	0.15	768.13 (646.45 to 908.96)	-0.21 (-0.24 to -0.18)
Angola	48.1	2200.14 (2179.84 to 2220.61)	101.31	1398.37 (1389.56 to 1407.23)	-1.38 (-1.48 to -1.28)
Antigua and Barbuda	0.15	947.7 (800.89 to 1117.13)	0.18	736.96 (633.32 to 853.79)	-0.9 (-0.94 to -0.86)
Argentina	68.86	876.79 (870.24 to 883.37)	85.23	708.55 (703.79 to 713.32)	-0.56 (-0.62 to -0.49)
Armenia	10.1	1126.41 (1103.82 to 1149.42)	6.42	844.97 (824.01 to 866.39)	-0.72 (-0.89 to -0.55)
Australia	49.97	1111.99 (1102.25 to 1121.8)	60.72	994.49 (986.53 to 1002.49)	-0.24 (-0.3 to -0.17)
Austria	22.47	1116.43 (1101.8 to 1131.21)	24.24	1212.76 (1197.35 to 1228.35)	0.38 (0.22 to 0.54)
Azerbaijan	24.3	1258.69 (1241.92 to 1275.67)	28.24	989.95 (978.33 to 1001.69)	-0.43 (-0.57 to -0.28)
Bahamas	0.65	908.7 (838.58 to 983.81)	0.77	711.13 (661.56 to 763.48)	-0.89 (-0.95 to -0.84)
Bahrain	1.77	1483.41 (1410.28 to 1560.34)	3.54	1067 (1032.07 to 1102.85)	-1.24 (-1.33 to -1.14)
Bangladesh	426.74	1860.99 (1855.11 to 1866.89)	481.24	1051.07 (1048.09 to 1054.06)	-1.71 (-1.83 to -1.59)
Barbados	0.6	878.88 (809.41 to 953.28)	0.52	724.95 (663.74 to 790.58)	-0.58 (-0.65 to -0.51)
Belarus	42.5	1636.26 (1620.66 to 1651.98)	34.88	1597.73 (1580.43 to 1615.2)	0.28 (0.11 to 0.46)
Belgium	29.04	1172.78 (1159.29 to 1186.4)	27.37	1092.03 (1079.02 to 1105.17)	0.06 (-0.3 to 0.42)
Belize	0.53	1359.74 (1239.72 to 1490.38)	0.98	830.73 (779.13 to 885.04)	-1.45 (-1.53 to -1.36)
Benin	20.8	1956.36 (1928.6 to 1984.47)	42.35	1374.13 (1360.61 to 1387.77)	-0.88 (-0.98 to -0.77)
Bermuda	0.15	864.68 (732.46 to 1016.7)	0.1	743.14 (602.4 to 910.33)	-0.47 (-0.54 to -0.4)
Bhutan	2.42	1835.31 (1758.85 to 1914.74)	2.07	991.32 (948.74 to 1035.47)	-2.04 (-2.11 to -1.97)
Bolivia (Plurinational State of)	23.28	1598.17 (1577.22 to 1619.35)	31.21	1003.45 (992.29 to 1014.71)	-1.35 (-1.53 to -1.16)
Bosnia and Herzegovina	11.69	991.8 (973.83 to 1010.03)	6.73	910.71 (888.75 to 933.13)	-0.19 (-0.28 to -0.1)
Botswana	4.23	1393.35 (1349.62 to 1438.36)	7.09	1032.92 (1008.9 to 1057.42)	-0.91 (-0.94 to -0.89)
Brazil	440.44	1160.44 (1156.94 to 1163.94)	570.12	954.92 (952.43 to 957.4)	-1.07 (-1.32 to -0.82)
Brunei Darussalam	1.21	1832.08 (1725.33 to 1945)	1.58	1237.19 (1176.81 to 1300.08)	-1.29 (-1.4 to -1.18)
Bulgaria	21.36	1039.07 (1025.1 to 1053.18)	13.96	977.92 (961.15 to 994.93)	0.07 (-0.03 to 0.16)
Burkina Faso	42.22	2129.31 (2108.47 to 2150.33)	75.87	1457.92 (1447.18 to 1468.73)	-1.29 (-1.32 to -1.26)
Burundi	22.44	1851.11 (1825.9 to 1876.63)	40.18	1369.8 (1355.86 to 1383.87)	-0.91 (-0.95 to -0.86)
Cabo Verde	0.97	1278.71 (1193.79 to 1369.09)	1.26	828.72 (783.13 to 876.5)	-1.5 (-1.55 to -1.45)
Cambodia	55.56	2333.96 (2313.96 to 2354.1)	57.67	1279.2 (1268.7 to 1289.77)	-1.87 (-2.05 to -1.69)
Cameroon	39.19	1725.05 (1707.29 to 1742.97)	83.03	1104.73 (1097 to 1112.52)	-1.24 (-1.4 to -1.07)
Canada	63.37	830.35 (823.86 to 836.88)	60.72	703.04 (697.42 to 708.7)	-1.46 (-2.02 to -0.89)
Central African Republic	10.58	1710.92 (1677.26 to 1745.16)	15.98	1218.65 (1199.37 to 1238.19)	-1.06 (-1.15 to -0.98)
Chad	28.43	2271.74 (2244.57 to 2299.19)	74.57	2106.18 (2090.42 to 2122.05)	-0.19 (-0.23 to -0.15)
Chile	44.55	1274.14 (1262.19 to 1286.18)	55.74	1147.13 (1137.61 to 1156.72)	-0.13 (-0.51 to 0.25)
China	3583.59	1176.65 (1175.4 to 1177.9)	2583.36	755.68 (754.74 to 756.63)	-1.58 (-1.76 to -1.4)
Colombia	89.51	1048.39 (1041.31 to 1055.52)	96.95	728.57 (723.98 to 733.18)	-1.17 (-1.19 to -1.15)
Comoros	1.64	1665.38 (1582.58 to 1751.89)	1.65	864.17 (822.67 to 907.31)	-2.07 (-2.1 to -2.03)
Congo	8.23	1555.55 (1520.51 to 1591.3)	14.63	1052.23 (1035.14 to 1069.55)	-0.98 (-1.08 to -0.87)
Cook Islands	0.08	1823.38 (1437.28 to 2288.86)	0.06	1402.73 (1068.34 to 1813.1)	-0.82 (-0.85 to -0.79)

Costa Rica	7.99	1035.82 (1012.48 to 1059.65)	9.29	703.95 (689.68 to 718.45)	-1.18 (-1.29 to -1.07)
Côte d'Ivoire	49.86	1899.55 (1881.9 to 1917.36)	81.6	1260.04 (1251.19 to 1268.94)	-1.19 (-1.24 to -1.15)
Croatia	11.44	939.62 (922.45 to 957.04)	7.26	804.53 (785.86 to 823.57)	-0.56 (-0.76 to -0.37)
Cuba	29.4	966.38 (955.18 to 977.69)	21.22	831.48 (820.19 to 842.91)	-0.28 (-0.35 to -0.2)
Cyprus	2.01	1010.14 (966.43 to 1055.35)	2.98	808.24 (778.45 to 839.1)	-0.66 (-0.76 to -0.56)
Czechia	27.64	1099.01 (1085.96 to 1112.19)	23.69	1032.15 (1018.48 to 1045.98)	-0.02 (-0.24 to 0.21)
Democratic People's Republic of Korea	128.44	2358.31 (2345.26 to 2371.4)	87.19	1286.75 (1278.2 to 1295.35)	-1.88 (-1.99 to -1.78)
Democratic Republic of the Congo	167.03	2065.43 (2055.15 to 2075.74)	253.44	1258.73 (1253.69 to 1263.79)	-1.51 (-1.67 to -1.34)
Denmark	9.51	724.71 (710.15 to 739.51)	8.71	684.8 (670.39 to 699.46)	-0.25 (-0.37 to -0.13)
Djibouti	1.72	1877.51 (1785.9 to 1973.31)	3.29	1018.89 (984.23 to 1054.54)	-1.97 (-2.07 to -1.87)
Dominica	0.19	1181.64 (1015.92 to 1368.79)	0.13	767.59 (638.9 to 915.15)	-1.42 (-1.51 to -1.32)
Dominican Republic	23.05	1277.04 (1259.84 to 1294.46)	27.03	931.87 (920.75 to 943.09)	-1.04 (-1.1 to -0.98)
Ecuador	29.49	1219.44 (1205.1 to 1233.92)	39.45	834.49 (826.25 to 842.8)	-1.19 (-1.26 to -1.12)
Egypt	232.87	1832.87 (1825.3 to 1840.47)	365.06	1424.82 (1420.17 to 1429.48)	-0.67 (-0.73 to -0.6)
El Salvador	13.56	1083.25 (1064.43 to 1102.37)	12.69	705.62 (693.33 to 718.09)	-1.42 (-1.6 to -1.25)
Equatorial Guinea	2.06	2206.87 (2110.04 to 2307.42)	3.7	1048 (1013.59 to 1083.45)	-2.59 (-2.67 to -2.52)
Eritrea	12.22	1639.04 (1609.18 to 1669.38)	15.27	951.35 (936.07 to 966.84)	-1.77 (-1.85 to -1.68)
Estonia	6.54	1689.14 (1648.32 to 1730.76)	4.33	1522.54 (1476.49 to 1569.81)	0.11 (-0.04 to 0.26)
Eswatini	2.88	1572.53 (1512.87 to 1634.28)	3.15	1008.05 (972.3 to 1044.97)	-1.4 (-1.43 to -1.38)
Ethiopia	194.07	1812.62 (1804.23 to 1821.05)	273.59	1045.92 (1041.86 to 1050)	-1.8 (-1.86 to -1.73)
Fiji	3.21	1702.66 (1643.26 to 1763.85)	3.37	1488.87 (1438.94 to 1540.13)	-0.34 (-0.4 to -0.28)
Finland	14.39	1119.01 (1100.59 to 1137.69)	11.21	975.98 (957.87 to 994.37)	-0.95 (-1.21 to -0.68)
France	128.02	877.78 (872.96 to 882.62)	119.64	845.13 (840.31 to 849.97)	-0.05 (-0.12 to 0.03)
Gabon	3.27	1554.27 (1498.31 to 1612.15)	4.38	921.95 (894.36 to 950.25)	-1.62 (-1.69 to -1.56)
Gambia	4.72	2165.3 (2099.58 to 2233.01)	6.83	1163.56 (1135.02 to 1192.75)	-2.08 (-2.17 to -2)
Georgia	16.39	1165.2 (1147.31 to 1183.31)	8.54	1069.34 (1046.44 to 1092.66)	0.04 (-0.1 to 0.17)
Germany	211.61	1055.88 (1051.35 to 1060.43)	178.39	1028.25 (1023.42 to 1033.09)	0.05 (-0.2 to 0.3)
Ghana	51.96	1523.93 (1510.34 to 1537.62)	80.9	895 (888.74 to 901.29)	-1.67 (-1.7 to -1.63)
Greece	21.39	847.45 (836.12 to 858.9)	18.06	831.93 (819.43 to 844.59)	0.02 (-0.02 to 0.07)
Greenland	0.14	935.43 (782.64 to 1111.9)	0.1	796.03 (649.04 to 968)	-0.58 (-0.64 to -0.52)
Grenada	0.2	1069.64 (920.87 to 1239.07)	0.21	792.42 (687.14 to 910.08)	-0.89 (-0.98 to -0.79)
Guam	0.61	1766.95 (1626.31 to 1918.46)	0.58	1607.57 (1478.83 to 1744.86)	-0.33 (-0.46 to -0.2)
Guatemala	29.04	1683.42 (1663.46 to 1703.6)	36.07	832.95 (824.24 to 841.75)	-2.31 (-2.51 to -2.11)
Guinea	27.33	2055.96 (2031.17 to 2081)	43.35	1363.23 (1350 to 1376.58)	-1.32 (-1.35 to -1.29)
Guinea-Bissau	3.71	1677.1 (1621.65 to 1734.23)	5.34	1047.94 (1019.16 to 1077.46)	-1.52 (-1.55 to -1.48)
Guyana	2.22	1133.12 (1084.5 to 1183.68)	1.72	841.43 (801.71 to 882.71)	-0.86 (-0.91 to -0.82)
Haiti	24.56	1667.83 (1646.43 to 1689.47)	32.57	931.65 (921.48 to 941.91)	-1.89 (-1.93 to -1.84)
Honduras	15.44	1555.05 (1529.63 to 1580.84)	22.69	808.16 (797.5 to 818.93)	-2.16 (-2.34 to -1.99)
Hungary	26.06	1042.21 (1029.46 to 1055.08)	19.97	932.9 (919.59 to 946.38)	-0.19 (-0.29 to -0.09)
Iceland	0.27	417.31 (368.63 to 471.03)	0.44	546.26 (496.21 to 600.25)	1.19 (0.92 to 1.46)
India	3585.6	1841.13 (1839.19 to 1843.07)	4117.14	1094.01 (1092.95 to 1095.07)	-1.76 (-1.8 to -1.72)
Indonesia	811.9	1770.61 (1766.66 to 1774.57)	966.98	1271.81 (1269.27 to 1274.34)	-1.01 (-1.07 to -0.95)
Iran (Islamic Republic of)	183.65	1524.57 (1517.3 to 1531.88)	268.33	1117.59 (1113.29 to 1121.92)	-0.91 (-1.21 to -0.61)
Iraq	100.62	2639.65 (2622.64 to 2656.76)	144.43	1401.02 (1393.73 to 1408.33)	-2.31 (-2.4 to -2.22)
Ireland	8.22	944.74 (924.35 to 965.48)	9.79	842.95 (826 to 860.18)	-0.27 (-0.34 to -0.21)
Israel	13.24	1096.82 (1078.06 to 1115.85)	25.2	1140.92 (1126.85 to 1155.12)	-0.02 (-0.09 to 0.05)

Italy	131.73	916.85 (911.89 to 921.82)	102.59	859.35 (853.94 to 864.79)	-0.13 (-0.2 to -0.05)
Jamaica	6.12	1066.19 (1038.43 to 1094.61)	6.11	778.62 (759.14 to 798.49)	-0.99 (-1.03 to -0.95)
Japan	468.57	1455.49 (1451.26 to 1459.72)	328.75	1253.84 (1249.41 to 1258.29)	-0.68 (-0.83 to -0.52)
Jordan	17.7	2285.27 (2249.51 to 2321.53)	47.14	1570.28 (1556.02 to 1584.64)	-1.28 (-1.38 to -1.18)
Kazakhstan	49.83	1194.29 (1183.64 to 1205.03)	54.14	1123.31 (1113.8 to 1132.89)	0.26 (0.06 to 0.46)
Kenya	81.99	1723.55 (1710.98 to 1736.22)	117.74	931.5 (926.03 to 936.99)	-1.93 (-1.96 to -1.91)
Kiribati	0.41	2271.67 (2050.08 to 2513.7)	0.57	1821.02 (1673.07 to 1979.58)	-0.63 (-0.66 to -0.6)
Kuwait	5.73	1340.03 (1303.77 to 1377.29)	16.87	1090.38 (1073.31 to 1107.71)	-1.05 (-1.21 to -0.88)
Kyrgyzstan	14.53	1393.13 (1369.42 to 1417.23)	20.65	1178.41 (1162.31 to 1194.7)	-0.09 (-0.32 to 0.15)
Lao People's Democratic Republic	20.23	2231.31 (2199.79 to 2263.2)	26.05	1329.81 (1313.55 to 1346.24)	-1.83 (-1.94 to -1.71)
Latvia	9.94	1507.01 (1477.46 to 1537.04)	5.77	1455.18 (1416.76 to 1494.5)	0.04 (-0.25 to 0.34)
Lebanon	14.27	1929.56 (1897.57 to 1961.99)	22.12	1437.81 (1418.68 to 1457.16)	-0.92 (-0.97 to -0.88)
Lesotho	5.82	1611.95 (1570.26 to 1654.55)	5.43	1099.93 (1070.13 to 1130.46)	-1.26 (-1.35 to -1.18)
Liberia	10.12	1889.49 (1850.86 to 1928.86)	13.23	989.99 (972.92 to 1007.31)	-2.09 (-2.17 to -2.02)
Libya	18.9	2261.29 (2227.18 to 2295.87)	22.07	1102.63 (1088.1 to 1117.31)	-2.07 (-2.34 to -1.79)
Lithuania	17.59	1880.2 (1852.48 to 1908.24)	10.16	1717.12 (1683.47 to 1751.35)	-0.11 (-0.37 to 0.14)
Luxembourg	0.97	966.63 (906.21 to 1030.52)	1.41	882.79 (836.45 to 931.37)	-0.87 (-1.08 to -0.66)
Madagascar	42.04	1657.89 (1641.34 to 1674.58)	67.56	983.99 (976.37 to 991.65)	-1.71 (-1.76 to -1.65)
Malawi	37.26	1739.43 (1720.88 to 1758.17)	43.45	936.15 (926.98 to 945.4)	-2.21 (-2.34 to -2.07)
Malaysia	66.58	1530.31 (1518.41 to 1542.28)	104.34	1229.07 (1221.59 to 1236.59)	-0.86 (-0.99 to -0.73)
Maldives	1.11	2546.04 (2388.48 to 2712.51)	1.52	1272.78 (1208.94 to 1339.4)	-2.03 (-2.33 to -1.74)
Mali	39.66	2154.32 (2132.58 to 2176.26)	86.3	1705.61 (1693.72 to 1717.58)	-0.76 (-0.79 to -0.73)
Malta	0.72	756.68 (701.93 to 814.8)	0.63	651.47 (600.44 to 706.28)	-0.32 (-0.42 to -0.22)
Marshall Islands	0.19	2149.49 (1839.04 to 2509.92)	0.22	1498.79 (1304.6 to 1714.94)	-0.95 (-1.05 to -0.85)
Mauritania	8.27	1844.84 (1803.86 to 1886.64)	11.38	1117.48 (1096.49 to 1138.81)	-1.54 (-1.62 to -1.47)
Mauritius	3.55	1184.73 (1145.33 to 1225.32)	3.29	1021.67 (986.88 to 1057.41)	-0.51 (-0.56 to -0.46)
Mexico	256.21	1238.13 (1233.16 to 1243.11)	291.62	830.9 (827.89 to 833.92)	-1.23 (-1.34 to -1.12)
Micronesia (Federated States of)	0.55	2621.27 (2398.17 to 2862.97)	0.38	1522.65 (1371.9 to 1685.98)	-1.63 (-1.75 to -1.5)
Monaco	0.06	872.14 (663.61 to 1138.64)	0.06	816.44 (615.95 to 1065.89)	-0.21 (-0.23 to -0.19)
Mongolia	7.86	1572.83 (1536.06 to 1610.39)	10.44	1210.97 (1187.77 to 1234.54)	-0.22 (-0.58 to 0.14)
Montenegro	1.68	1073.5 (1022.68 to 1126.31)	1.43	989.43 (938.36 to 1042.71)	-0.21 (-0.25 to -0.18)
Morocco	102.5	1674.66 (1664.12 to 1685.27)	110.45	1139.02 (1132.31 to 1145.76)	-1.32 (-1.38 to -1.27)
Mozambique	48.08	1616.48 (1601.75 to 1631.32)	81.38	1149.6 (1141.37 to 1157.88)	-0.96 (-1.06 to -0.87)
Myanmar	177.44	1781.1 (1772.53 to 1789.7)	178.95	1198.64 (1193.08 to 1204.21)	-1.21 (-1.26 to -1.15)
Namibia	5.05	1584.12 (1538.68 to 1630.74)	7.5	1149.71 (1123.5 to 1176.41)	-0.95 (-1.01 to -0.88)
Nauru	0.07	2901.61 (2236.12 to 3728.81)	0.05	1830.74 (1352.8 to 2437.49)	-1.52 (-1.57 to -1.47)
Nepal	100.2	2304.69 (2290.07 to 2319.38)	93.83	1043.11 (1036.37 to 1049.89)	-2.65 (-2.77 to -2.53)
Netherlands	36.68	912.83 (903.49 to 922.25)	30.75	833.49 (824.16 to 842.92)	-0.21 (-0.26 to -0.16)
New Zealand	14.97	1650.94 (1624.55 to 1677.67)	15.63	1297.44 (1277.11 to 1318.03)	-0.44 (-0.68 to -0.19)
Nicaragua	11.27	1322.66 (1297.01 to 1348.76)	13.77	758.63 (745.95 to 771.49)	-1.56 (-1.77 to -1.35)
Niger	36.91	2258.64 (2234.76 to 2282.76)	119.71	2520.75 (2505.72 to 2535.86)	0.4 (0.34 to 0.45)
Nigeria	377.7	2003.52 (1996.78 to 2010.29)	755.58	1408.94 (1405.65 to 1412.22)	-1.09 (-1.17 to -1.01)
Niue	0.01	1935.34 (892.12 to 3685.53)	0.01	1424.85 (487.49 to 3294.99)	-0.96 (-1.01 to -0.91)
North Macedonia	5.68	1114.76 (1085.93 to 1144.19)	5.12	941.01 (915.09 to 967.57)	-0.5 (-0.6 to -0.4)
Northern Mariana Islands	0.22	1618.74 (1403.49 to 1865.18)	0.15	1343.14 (1132.58 to 1583.51)	-0.5 (-0.54 to -0.46)
Norway	8.02	752.57 (736.17 to 769.26)	8.82	716.83 (701.86 to 732.05)	-0.1 (-0.18 to -0.02)

Oman	7.98	2436.63 (2381.65 to 2492.73)	12.59	1200.16 (1178.93 to 1221.72)	-2.18 (-2.42 to -1.95)
Pakistan	653.09	2983.22 (2975.81 to 2990.64)	943.7	1586.25 (1583.01 to 1589.49)	-2.09 (-2.19 to -1.99)
Palau	0.06	1600.05 (1226.53 to 2063.66)	0.05	1310.09 (959.09 to 1757.87)	-0.6 (-0.63 to -0.57)
Palestine	11.79	2858.87 (2804.54 to 2914.11)	18.93	1495.17 (1473.44 to 1517.17)	-2.44 (-2.61 to -2.26)
Panama	5.65	944 (918.91 to 969.68)	8.13	759.03 (742.59 to 775.76)	-0.62 (-0.68 to -0.55)
Papua New Guinea	21.97	2427.7 (2394.62 to 2461.18)	50.71	1987.09 (1969.66 to 2004.65)	-0.66 (-0.69 to -0.63)
Paraguay	14.16	1563.4 (1537.02 to 1590.16)	16.14	855.54 (842.3 to 868.94)	-1.85 (-1.92 to -1.79)
Peru	58.56	1123.19 (1113.82 to 1132.62)	76.76	785.77 (780.21 to 791.36)	-1.08 (-1.13 to -1.02)
Philippines	248.63	1700.51 (1693.63 to 1707.41)	412.6	1438.36 (1433.94 to 1442.78)	-0.54 (-0.58 to -0.49)
Poland	94.52	997.79 (991.36 to 1004.25)	82.29	905.99 (899.62 to 912.39)	-0.33 (-0.52 to -0.14)
Portugal	16.13	641.41 (631.54 to 651.4)	13.33	574.68 (564.65 to 584.85)	-0.33 (-0.36 to -0.29)
Puerto Rico	9.13	962.34 (942.67 to 982.32)	5.28	695.02 (676.27 to 714.18)	-1.07 (-1.13 to -1.01)
Qatar	1.63	2020.18 (1919.28 to 2126.27)	7.54	1275.12 (1244.87 to 1306.04)	-1.58 (-1.62 to -1.55)
Republic of Korea	175.21	1412.69 (1405.97 to 1419.44)	145.54	1187.25 (1180.99 to 1193.55)	-0.55 (-0.61 to -0.48)
Republic of Moldova	20.43	1783.18 (1758.63 to 1808.01)	13.25	1447.47 (1422.15 to 1473.2)	-0.38 (-0.53 to -0.22)
Romania	54.42	984.59 (976.27 to 992.96)	38.87	967.24 (957.37 to 977.19)	0.11 (0 to 0.21)
Russian Federation	649.79	1703.36 (1699.18 to 1707.54)	593.27	1710.83 (1706.31 to 1715.37)	0.45 (0.27 to 0.63)
Rwanda	26.4	1723.06 (1701.31 to 1745.07)	32.62	963.34 (952.72 to 974.07)	-2.09 (-2.17 to -2)
Saint Kitts and Nevis	0.09	954.67 (758.42 to 1199.04)	0.12	725.16 (598.47 to 872.16)	-0.85 (-0.87 to -0.82)
Saint Lucia	0.36	1111.6 (995.17 to 1239.84)	0.34	732.15 (656.2 to 815.04)	-1.31 (-1.39 to -1.23)
Saint Vincent and the Grenadines	0.26	1050.52 (920.38 to 1197.04)	0.22	796.67 (695.16 to 909.33)	-0.85 (-0.9 to -0.8)
Samoa	0.55	1646.68 (1506.34 to 1797.92)	0.7	1520.21 (1408.2 to 1639.21)	-0.04 (-0.13 to 0.04)
San Marino	0.05	846.74 (633.88 to 1110.15)	0.06	797.46 (597.66 to 1046.23)	-0.19 (-0.22 to -0.17)
Sao Tome and Principe	0.36	1523.72 (1363.52 to 1700.05)	0.47	879.28 (800.78 to 964.06)	-1.85 (-1.9 to -1.79)
Saudi Arabia	64.25	2062.33 (2045.68 to 2079.1)	112.37	1061.99 (1055.73 to 1068.28)	-2.3 (-2.43 to -2.16)
Senegal	31.55	1951.76 (1929.35 to 1974.4)	43.95	1171.3 (1160.1 to 1182.59)	-1.42 (-1.52 to -1.33)
Serbia	23.14	988.35 (975.65 to 1001.17)	17.64	863.8 (850.96 to 876.79)	-0.55 (-0.77 to -0.33)
Seychelles	0.23	1312.68 (1140.52 to 1506.7)	0.3	1217.92 (1083.11 to 1365.36)	-0.07 (-0.16 to 0.03)
Sierra Leone	17.8	1802.52 (1774.81 to 1830.63)	26	1189.37 (1174.36 to 1204.56)	-1.38 (-1.51 to -1.26)
Singapore	13.02	1373.11 (1349.42 to 1397.14)	16.89	1081.45 (1064.14 to 1099.04)	-0.76 (-0.84 to -0.68)
Slovakia	13.98	1056.05 (1038.57 to 1073.75)	11.79	919.17 (902.18 to 936.43)	-0.24 (-0.4 to -0.09)
Slovenia	6.12	1209.76 (1179.62 to 1240.51)	5.05	1188.35 (1154.78 to 1222.76)	0.18 (0.07 to 0.29)
Solomon Islands	2.25	3408.48 (3262.46 to 3560.08)	3.4	2081.09 (2011.12 to 2153)	-1.63 (-1.64 to -1.61)
Somalia	31.89	1995.53 (1973.32 to 2017.96)	72.68	1632.57 (1620.34 to 1644.89)	-0.58 (-0.64 to -0.53)
South Africa	129.29	1386.57 (1378.79 to 1394.38)	174.72	1110.61 (1105.39 to 1115.84)	-0.73 (-0.74 to -0.72)
South Sudan	19.13	1570.43 (1546.96 to 1594.24)	29.2	1377.87 (1361.81 to 1394.09)	-0.36 (-0.41 to -0.31)
Spain	66.13	688.53 (683.28 to 693.81)	65.47	655.31 (650.08 to 660.58)	-0.11 (-0.15 to -0.07)
Sri Lanka	55.19	1224.43 (1214.11 to 1234.82)	59.8	1059.7 (1051.2 to 1068.25)	-0.36 (-0.39 to -0.32)
Sudan	131.9	2970.59 (2953.99 to 2987.26)	175.53	1609.23 (1601.55 to 1616.93)	-2.26 (-2.44 to -2.08)
Suriname	1.09	1149.11 (1080.14 to 1221.62)	1.33	918.74 (870.09 to 969.43)	-0.71 (-0.76 to -0.65)
Sweden	16.74	799.35 (787.18 to 811.67)	20.93	938.36 (925.58 to 951.29)	0.59 (0.41 to 0.77)
Switzerland	19.81	1101.03 (1085.65 to 1116.59)	20.59	1029.97 (1015.68 to 1044.42)	-0.09 (-0.21 to 0.02)
Syrian Arab Republic	63.3	2459.96 (2439.78 to 2480.29)	45.46	1277.42 (1264.96 to 1289.98)	-2.13 (-2.22 to -2.05)
Taiwan (Province of China)	113.38	2098.5 (2086.05 to 2111.01)	113.26	1795.25 (1784.54 to 1806.02)	-0.47 (-0.76 to -0.19)
Tajikistan	21.82	1855.7 (1829.05 to 1882.73)	29.47	1144.37 (1131.18 to 1157.69)	-1.3 (-1.46 to -1.14)
Thailand	185.91	1182.44 (1176.97 to 1187.93)	170.91	1014.61 (1009.71 to 1019.53)	-0.43 (-0.47 to -0.39)

Timor-Leste	4.87	2679.08 (2601.97 to 2758.17)	5.51	1724.06 (1677.11 to 1772.09)	-1.63 (-1.76 to -1.49)
Togo	14.97	1841.23 (1810.61 to 1872.31)	23.36	1109.09 (1094.77 to 1123.56)	-1.46 (-1.56 to -1.37)
Tokelau	0.01	3067.25 (1464.61 to 5808.91)	0.01	2022.61 (768.54 to 4378.85)	-1.29 (-1.34 to -1.25)
Tonga	0.42	2057.2 (1859.37 to 2271.56)	0.42	1733.56 (1569.95 to 1910.14)	-0.46 (-0.49 to -0.43)
Trinidad and Tobago	3.11	1018.77 (982.71 to 1055.95)	2.72	791.26 (761.51 to 821.94)	-0.67 (-0.76 to -0.58)
Tunisia	32.11	1606.06 (1587.94 to 1624.36)	37.51	1198.86 (1186.67 to 1211.16)	-0.9 (-0.98 to -0.82)
Turkey	213.83	1545.07 (1538.38 to 1551.78)	261.1	1190.49 (1185.91 to 1195.08)	-1.02 (-1.1 to -0.95)
Turkmenistan	14.4	1643.16 (1614.63 to 1672.17)	14.88	1181.92 (1162.97 to 1201.12)	-0.77 (-0.98 to -0.55)
Tuvalu	0.05	2001.66 (1476.03 to 2663.98)	0.04	1491.44 (1070 to 2031.34)	-0.91 (-0.94 to -0.88)
Uganda	68.84	1925.13 (1909.78 to 1940.6)	113.22	1166.88 (1159.76 to 1174.04)	-1.75 (-1.87 to -1.64)
Ukraine	223.21	1734.38 (1727.18 to 1741.61)	167.19	1601.02 (1593.05 to 1609.03)	0.04 (-0.12 to 0.19)
United Arab Emirates	5.89	1660.07 (1614.8 to 1706.7)	17.86	1020.41 (1003.48 to 1037.59)	-1.51 (-1.66 to -1.35)
United Kingdom	154.35	1067.17 (1061.84 to 1072.53)	146.93	958.29 (953.36 to 963.24)	-0.28 (-0.32 to -0.23)
United Republic of Tanzania	100.82	1801.32 (1789.6 to 1813.11)	177.75	1264.06 (1258.02 to 1270.11)	-0.99 (-1.07 to -0.92)
United States of America	697.65	1003.2 (1000.84 to 1005.56)	490.79	635.07 (633.29 to 636.85)	-1.99 (-2.21 to -1.77)
United States Virgin Islands	0.3	1046.02 (929.79 to 1173.15)	0.14	800.31 (670.97 to 948.92)	-0.87 (-0.92 to -0.83)
Uruguay	7.46	1005.21 (982.51 to 1028.3)	7.42	876.01 (856.14 to 896.24)	-0.47 (-0.51 to -0.42)
Uzbekistan	69.78	1441.98 (1430.49 to 1453.56)	92.79	1017.51 (1010.96 to 1024.1)	-0.89 (-1.09 to -0.69)
Vanuatu	0.8	2442.43 (2270.02 to 2626.26)	1.22	1616.09 (1525.46 to 1711.03)	-1.35 (-1.39 to -1.31)
Venezuela (Bolivarian Republic of)	53.37	1130.02 (1120.15 to 1139.97)	52.81	776.39 (769.73 to 783.1)	-1.02 (-1.13 to -0.91)
Vietnam	228.39	1390.28 (1384.25 to 1396.34)	291.77	1108.65 (1104.61 to 1112.71)	-0.56 (-0.62 to -0.5)
Yemen	97.55	3764.83 (3740.31 to 3789.5)	135.32	1696.71 (1687.51 to 1705.96)	-2.86 (-3.05 to -2.66)
Zambia	29.14	1735.62 (1714.45 to 1757.02)	48.2	1033.92 (1024.33 to 1043.6)	-1.65 (-1.75 to -1.54)
Zimbabwe	39.97	1794.02 (1775.49 to 1812.72)	51.78	1326.43 (1314.81 to 1338.15)	-0.67 (-0.8 to -0.53)

S16 Table. The global prevalence of genital prolapses among women aged 15-49 years in 1990 and 2021, along with the trends and changes observed between these years, by country and territories

Countries	Number of cases (Thousands) in 1990	Age-standardized rate per 100,000 population (95% UI) in 2021	Number of cases (Thousands) in 2021	Age-standardized rate per 100,000 population (95% UI) in 2021	Estimated annual percentage changes (95% CI) from 1990 to 2021
Afghanistan	61.87	3395.36 (3368.41 to 3422.52)	169.89	3338.07 (3322.04 to 3354.17)	0.04 (-0.07 to 0.16)
Albania	4.08	773.64 (749.99 to 797.86)	3.93	568.27 (550.61 to 586.39)	-0.97 (-1.1 to -0.83)
Algeria	108.39	2971.52 (2953.66 to 2989.46)	294.11	2552.18 (2542.94 to 2561.45)	-0.28 (-0.34 to -0.22)
American Samoa	0.07	856.59 (666.15 to 1086.7)	0.08	672.86 (535.15 to 837.84)	-0.79 (-0.82 to -0.77)
Andorra	0.25	1751.28 (1539.81 to 1985.12)	0.47	1704.95 (1550.12 to 1875.68)	-0.03 (-0.09 to 0.02)
Angola	33.55	2058.32 (2036 to 2080.84)	94.62	1665.51 (1654.78 to 1676.28)	-0.62 (-0.67 to -0.56)
Antigua and Barbuda	0.43	3164.43 (2866.78 to 3486.45)	0.57	2147.04 (1973.51 to 2333.05)	-1.34 (-1.39 to -1.29)
Argentina	130.15	1730.77 (1721.38 to 1740.21)	179.52	1467.97 (1461.19 to 1474.78)	-0.42 (-0.49 to -0.36)
Armenia	2.56	456.56 (438.8 to 474.87)	2.81	357.12 (344 to 370.69)	-0.24 (-0.58 to 0.1)
Australia	29.87	698.49 (690.56 to 706.5)	47.47	692.88 (686.65 to 699.15)	0.18 (0.07 to 0.29)
Austria	45.64	2257.46 (2236.74 to 2278.33)	32.12	1366.57 (1351.58 to 1381.72)	-2.16 (-2.78 to -1.54)
Azerbaijan	4.97	466.95 (453.85 to 480.34)	9.72	358.83 (351.72 to 366.05)	-0.34 (-0.58 to -0.09)
Bahamas	1.77	3039.37 (2897.1 to 3187.14)	2.37	2123.95 (2039.19 to 2211.42)	-1.26 (-1.33 to -1.19)
Bahrain	1.82	2544.08 (2423.7 to 2669.25)	6.23	1878.23 (1831.76 to 1925.62)	-1.04 (-1.09 to -0.99)
Bangladesh	1012.51	5949.87 (5937.98 to 5961.79)	1455.32	3438.52 (3432.93 to 3444.13)	-1.67 (-1.69 to -1.65)
Barbados	1.68	2773.63 (2641.02 to 2911.49)	1.67	2070.1 (1971.23 to 2173.09)	-0.89 (-0.98 to -0.8)
Belarus	16.48	719.21 (708.23 to 730.33)	18.91	692.86 (682.99 to 702.88)	0.78 (0.39 to 1.16)
Belgium	45.68	1857.96 (1840.89 to 1875.15)	55.03	1923.42 (1907.32 to 1939.65)	-0.28 (-0.72 to 0.16)
Belize	1.07	3775.11 (3544.08 to 4018.3)	2.54	2406.51 (2313.47 to 2502.42)	-1.41 (-1.44 to -1.38)
Benin	23.08	3009.66 (2969.88 to 3049.86)	58.35	2490.68 (2470.15 to 2511.35)	-0.49 (-0.55 to -0.43)
Bermuda	0.48	2723.24 (2483.57 to 2982.37)	0.36	2119.53 (1901.77 to 2360.33)	-0.83 (-0.94 to -0.73)
Bhutan	5.93	6098.49 (5940.36 to 6260.02)	6.95	3673.64 (3587.03 to 3761.92)	-1.65 (-1.69 to -1.6)
Bolivia (Plurinational State of)	46	3909.8 (3873.75 to 3946.12)	84.81	3017.69 (2997.35 to 3038.13)	-0.77 (-0.86 to -0.69)
Bosnia and Herzegovina	5.61	571.55 (556.66 to 586.74)	4.49	467.42 (453.82 to 481.41)	-0.51 (-0.68 to -0.33)
Botswana	3.68	1738.5 (1681.45 to 1797.07)	8.41	1364.56 (1335.28 to 1394.34)	-0.71 (-0.74 to -0.68)
Brazil	2000.63	5954.63 (5946.27 to 5963)	2230.33	3590.19 (3585.48 to 3594.92)	-2.06 (-2.21 to -1.91)
Brunei Darussalam	0.18	403.5 (345 to 469.63)	0.39	305.47 (275.76 to 337.73)	-0.92 (-1.05 to -0.79)
Bulgaria	13.52	590.6 (580.67 to 600.67)	11.45	545.09 (535.11 to 555.27)	0.21 (0.02 to 0.41)
Burkina Faso	47.8	3075 (3047.15 to 3103.05)	104.44	2687.79 (2671.12 to 2704.55)	-0.42 (-0.43 to -0.4)
Burundi	19.56	2222.75 (2190.8 to 2255.08)	42.43	1969.71 (1950.32 to 1989.24)	-0.32 (-0.35 to -0.29)
Cabo Verde	1.37	2704.8 (2558.23 to 2857.93)	2.5	1898.84 (1824.28 to 1975.78)	-1.11 (-1.22 to -1.01)
Cambodia	15.46	876.41 (862.4 to 890.6)	24.9	639.86 (631.88 to 647.93)	-1.01 (-1.08 to -0.95)
Cameroon	48.52	2893.08 (2866.91 to 2919.45)	126.23	2196.34 (2183.96 to 2208.77)	-0.74 (-0.86 to -0.62)
Canada	47.69	675.33 (669.25 to 681.44)	61.66	635.04 (630.03 to 640.08)	-0.17 (-0.23 to -0.11)
Central African Republic	8.96	1951.12 (1910.41 to 1992.5)	16.7	1610.43 (1585.84 to 1635.32)	-0.54 (-0.59 to -0.49)
Chad	30.36	3149.17 (3113.26 to 3185.41)	72.56	2772.98 (2752.39 to 2793.69)	-0.34 (-0.39 to -0.3)
Chile	47.5	1594.94 (1580.55 to 1609.44)	67.06	1337.84 (1327.73 to 1348.02)	-0.87 (-1.06 to -0.68)
China	1594.6	659.18 (658.14 to 660.22)	2022.07	495.32 (494.63 to 496.01)	-0.83 (-1.02 to -0.64)
Colombia	123.77	1951.21 (1940.14 to 1962.33)	177.34	1391.12 (1384.65 to 1397.62)	-1.09 (-1.1 to -1.07)
Comoros	1.63	2194.91 (2088.08 to 2305.97)	2.52	1483.32 (1425.83 to 1542.56)	-1.27 (-1.3 to -1.24)
Congo	7.17	1915.81 (1870.88 to 1961.58)	17.93	1459.7 (1438.29 to 1481.35)	-0.68 (-0.77 to -0.6)
Cook Islands	0.03	778.02 (517.52 to 1126.67)	0.03	640 (427.94 to 927.36)	-0.63 (-0.64 to -0.62)

Costa Rica	11.32	1984.2 (1946.89 to 2022.08)	16.26	1264.48 (1245.08 to 1284.13)	-1.44 (-1.53 to -1.34)
Côte d'Ivoire	53.63	2961.64 (2935.84 to 2987.63)	118.81	2340.44 (2326.83 to 2354.12)	-0.7 (-0.74 to -0.66)
Croatia	6.04	492.63 (480.29 to 505.23)	4.82	414.29 (402.65 to 426.25)	-0.37 (-0.56 to -0.18)
Cuba	74.99	2827.75 (2807.44 to 2848.17)	65.78	2281.79 (2264.05 to 2299.65)	-0.41 (-0.51 to -0.3)
Cyprus	3.92	2015.83 (1953.18 to 2080)	7.13	1643.79 (1605.63 to 1682.89)	-0.99 (-1.26 to -0.71)
Czechia	18.09	632.72 (623.51 to 642.04)	21.6	602.33 (594.28 to 610.5)	0.55 (0.27 to 0.82)
Democratic People's Republic of Korea	42.03	818.11 (810.28 to 826)	37.46	510.73 (505.55 to 515.95)	-1.42 (-1.48 to -1.37)
Democratic Republic of the Congo	122.52	2058.32 (2046.63 to 2070.07)	245.73	1590.89 (1584.53 to 1597.28)	-0.73 (-0.83 to -0.63)
Denmark	30.77	2152.06 (2127.97 to 2176.37)	31.53	2213.27 (2188.65 to 2238.11)	0.15 (0.1 to 0.19)
Djibouti	1.49	2215.93 (2102.52 to 2334.19)	4.76	1600.82 (1555.26 to 1647.44)	-0.99 (-1.06 to -0.91)
Dominica	0.48	3629.21 (3309.18 to 3972.51)	0.37	2307.73 (2077.65 to 2556.66)	-1.53 (-1.57 to -1.5)
Dominican Republic	50.48	3697.5 (3664.49 to 3730.75)	74.71	2770.81 (2750.94 to 2790.79)	-0.96 (-1 to -0.92)
Ecuador	80.1	4275.48 (4245.44 to 4305.69)	103.72	2372.29 (2357.86 to 2386.79)	-2.12 (-2.33 to -1.9)
Egypt	302.86	2989.79 (2979.11 to 3000.51)	593.87	2657.3 (2650.51 to 2664.11)	-0.19 (-0.26 to -0.13)
El Salvador	19.16	2043.05 (2013.85 to 2072.57)	22.1	1322.92 (1305.51 to 1340.51)	-1.4 (-1.51 to -1.29)
Equatorial Guinea	1.56	2104.22 (1999.96 to 2212.65)	3.87	1438.46 (1392.62 to 1485.49)	-1.25 (-1.31 to -1.18)
Eritrea	12.56	2171.13 (2132.85 to 2209.94)	21.64	1628.17 (1606.34 to 1650.24)	-0.88 (-0.93 to -0.82)
Estonia	3.02	756.01 (729.28 to 783.52)	2.46	692.19 (665.01 to 720.42)	0.63 (0.28 to 0.99)
Eswatini	2.44	1888.38 (1812.64 to 1966.6)	3.62	1511.26 (1461.33 to 1562.55)	-0.58 (-0.63 to -0.54)
Ethiopia	182.44	2297.98 (2287.19 to 2308.8)	375.86	1905.7 (1899.45 to 1911.96)	-0.56 (-0.6 to -0.52)
Fiji	1.11	725.45 (682.98 to 769.94)	1.42	656.54 (622.78 to 691.69)	-0.28 (-0.31 to -0.24)
Finland	30.18	2104.25 (2080.4 to 2128.33)	25.18	1968.01 (1943.72 to 1992.55)	-0.47 (-0.7 to -0.25)
France	284.66	1994.49 (1987.1 to 2001.9)	342.07	2088.79 (2081.75 to 2095.86)	0.3 (0.2 to 0.4)
Gabon	2.8	1908.34 (1836.61 to 1982.32)	5.33	1370.97 (1334.14 to 1408.58)	-1.02 (-1.03 to -1)
Gambia	4.45	3075.16 (2981.21 to 3171.52)	9.45	2183.42 (2138.41 to 2229.2)	-1.06 (-1.14 to -0.98)
Georgia	4.65	390.98 (379.8 to 402.41)	3.71	389.72 (377.26 to 402.57)	0.77 (0.43 to 1.11)
Germany	428.21	2115.25 (2108.91 to 2121.6)	425.88	2149.91 (2143.44 to 2156.4)	-0.02 (-0.2 to 0.16)
Ghana	71	2818.41 (2797.24 to 2839.71)	152.39	2049.74 (2039.33 to 2060.19)	-1 (-1.43 to -0.57)
Greece	41.81	1621.27 (1605.75 to 1636.9)	48.18	1689.81 (1674.43 to 1705.33)	0.4 (0.3 to 0.51)
Greenland	0.09	742.96 (593.57 to 919.13)	0.08	662.22 (521.33 to 829.94)	-0.48 (-0.56 to -0.4)
Grenada	0.48	3316.22 (3019.17 to 3636.21)	0.6	2372.29 (2184.68 to 2572.34)	-1.05 (-1.11 to -1)
Guam	0.21	786.61 (680.72 to 905.27)	0.28	723.94 (641.21 to 814.95)	-0.27 (-0.35 to -0.2)
Guatemala	31.2	2402.99 (2375.98 to 2430.24)	50.11	1402.63 (1390.28 to 1415.07)	-1.79 (-1.86 to -1.73)
Guinea	32.54	3038.61 (3005.18 to 3072.32)	56.56	2347.69 (2327.9 to 2367.62)	-0.81 (-0.85 to -0.77)
Guinea-Bissau	4.88	2939.29 (2855.71 to 3024.81)	8.75	2254.3 (2205.98 to 2303.46)	-0.78 (-0.85 to -0.7)
Guyana	4.94	3294.25 (3200.66 to 3390.05)	4.36	2352.32 (2282.77 to 2423.5)	-0.98 (-1.01 to -0.96)
Haiti	46.95	3939.5 (3903.24 to 3976.03)	86.6	2742.08 (2723.67 to 2760.58)	-1.14 (-1.15 to -1.13)
Honduras	17.26	2383.55 (2347.47 to 2420.07)	33.89	1478.69 (1462.86 to 1494.65)	-1.58 (-1.68 to -1.49)
Hungary	18.52	642.97 (633.74 to 652.32)	17.31	532.9 (524.95 to 540.98)	-0.34 (-0.53 to -0.14)
Iceland	1.6	2724.69 (2592.26 to 2862.35)	2.17	2510.79 (2406 to 2619.32)	0.18 (-0.1 to 0.45)
India	7874.05	4741.95 (4738.6 to 4745.29)	8899.9	2544.11 (2542.43 to 2545.78)	-2.47 (-2.66 to -2.27)
Indonesia	260	725.03 (722.21 to 727.85)	455.88	584.97 (583.27 to 586.67)	-0.65 (-0.7 to -0.6)
Iran (Islamic Republic of)	287.52	3533.34 (3520.23 to 3546.49)	765.64	2962.98 (2956.29 to 2969.68)	-0.07 (-0.63 to 0.49)
Iraq	86.52	3292.47 (3270.31 to 3314.76)	222.38	2490.64 (2480.28 to 2501.02)	-0.94 (-1.03 to -0.85)
Ireland	17.03	2082.55 (2051.29 to 2114.19)	28.83	2031.87 (2008.2 to 2055.77)	0.13 (0.04 to 0.22)
Israel	26.2	2405.1 (2375.56 to 2434.93)	58.32	2529.46 (2508.94 to 2550.11)	0.26 (0.23 to 0.29)

Italy	213.18	1463.71 (1457.5 to 1469.95)	215.07	1362.91 (1356.98 to 1368.86)	-0.38 (-1.18 to 0.44)
Jamaica	13.94	3209.89 (3155.53 to 3265.01)	15.78	2148.61 (2115.15 to 2182.47)	-1.28 (-1.31 to -1.24)
Japan	106.91	286.2 (284.47 to 287.94)	76.04	222.95 (221.32 to 224.59)	-0.87 (-0.93 to -0.8)
Jordan	12.97	2574.43 (2529.9 to 2619.57)	52.52	1968.21 (1951.38 to 1985.15)	-0.77 (-0.81 to -0.74)
Kazakhstan	14.07	480.14 (472.18 to 488.21)	22.03	441.94 (436.12 to 447.82)	0.42 (0.12 to 0.72)
Kenya	73.2	2245.22 (2228.5 to 2262.05)	155.18	1570.61 (1562.66 to 1578.6)	-1.03 (-1.08 to -0.98)
Kiribati	0.11	821.55 (673.68 to 993.39)	0.18	665.06 (570.39 to 771.45)	-0.72 (-0.74 to -0.69)
Kuwait	6.56	2350.92 (2291.85 to 2411.25)	33.22	1932.77 (1911.85 to 1953.93)	-0.78 (-1 to -0.56)
Kyrgyzstan	3.11	534.24 (515.28 to 553.74)	6.56	436.44 (425.93 to 447.15)	-0.03 (-0.31 to 0.25)
Lao People's Democratic Republic	6.22	892.03 (869.78 to 914.72)	11.34	683 (670.4 to 695.78)	-0.97 (-1.01 to -0.92)
Latvia	5.02	703.63 (684.27 to 723.43)	3.37	648.52 (626.67 to 671.13)	0.51 (0.24 to 0.77)
Lebanon	18.42	2967.91 (2925.08 to 3011.21)	36.08	2370.46 (2345.99 to 2395.14)	-0.62 (-0.66 to -0.59)
Lesotho	5.47	1793.73 (1746.26 to 1842.19)	5.45	1429.86 (1391.5 to 1469.05)	-0.69 (-0.73 to -0.66)
Liberia	11.41	3032.71 (2975.18 to 3091.15)	23.88	2134.53 (2107.29 to 2162.03)	-1.06 (-1.13 to -0.99)
Libya	16.39	3014.35 (2967.89 to 3061.38)	40.25	1892.41 (1873.95 to 1911.01)	-1.35 (-1.42 to -1.29)
Lithuania	7.14	759 (741.48 to 776.83)	5.15	691.52 (672.62 to 710.94)	0.42 (0.12 to 0.71)
Luxembourg	1.81	1799.67 (1717.67 to 1885.08)	3.21	1743.69 (1683.71 to 1805.71)	-0.47 (-0.67 to -0.27)
Madagascar	39.49	2151.6 (2129.9 to 2173.48)	92.29	1702.44 (1691.34 to 1713.6)	-0.66 (-0.73 to -0.59)
Malawi	34.36	2255.43 (2231.04 to 2280.04)	59.98	1759.52 (1745.08 to 1774.06)	-0.73 (-0.81 to -0.65)
Malaysia	24.87	751.99 (742.48 to 761.6)	46.19	608.24 (602.68 to 613.84)	-0.76 (-0.82 to -0.69)
Maldives	0.27	914.32 (807.55 to 1031.65)	0.66	588.89 (544.35 to 636.34)	-1.35 (-1.44 to -1.26)
Mali	45.37	3164.97 (3135.46 to 3194.7)	98.99	2687.89 (2670.75 to 2705.11)	-0.5 (-0.53 to -0.47)
Malta	1.97	1896.43 (1813.1 to 1982.92)	1.97	1729.35 (1653.45 to 1808.62)	-0.46 (-0.96 to 0.04)
Marshall Islands	0.05	854.94 (627.12 to 1144.61)	0.09	680.33 (547.25 to 836.63)	-0.62 (-0.67 to -0.58)
Mauritania	9.74	2899.23 (2840.68 to 2958.74)	17.01	2114.33 (2082.28 to 2146.77)	-0.92 (-0.99 to -0.85)
Mauritius	1.53	649.23 (616.51 to 683.31)	1.82	522.22 (498.4 to 546.95)	-0.73 (-0.77 to -0.7)
Mexico	286.02	1849.76 (1842.87 to 1856.67)	562.11	1587.16 (1583.01 to 1591.32)	-1.83 (-2.31 to -1.35)
Micronesia (Federated States of)	0.13	877.47 (730.29 to 1047.05)	0.15	636.78 (537.23 to 749.62)	-1.03 (-1.06 to -1)
Monaco	0.17	1969.5 (1683.72 to 2305.91)	0.17	1909.16 (1631.17 to 2229.08)	-0.04 (-0.08 to 0)
Mongolia	1.73	588.11 (560.51 to 616.73)	4.18	480.49 (466.03 to 495.31)	0.01 (-0.32 to 0.34)
Montenegro	0.86	636.79 (594.98 to 680.78)	0.96	538.72 (505.09 to 574.3)	-0.46 (-0.58 to -0.34)
Morocco	125.29	2793.33 (2777.77 to 2808.95)	224.33	2332.33 (2322.69 to 2342.01)	-0.46 (-0.57 to -0.34)
Mozambique	52.27	2171.55 (2152.76 to 2190.47)	99.7	1885.29 (1873.41 to 1897.23)	-0.33 (-0.38 to -0.29)
Myanmar	57.7	787.38 (780.86 to 793.94)	91.91	629.74 (625.68 to 633.83)	-0.69 (-0.72 to -0.67)
Namibia	4.17	1840.06 (1783.55 to 1897.96)	8.52	1571.09 (1537.65 to 1605.1)	-0.41 (-0.44 to -0.38)
Nauru	0.02	910.11 (520.32 to 1490.01)	0.02	717.52 (411.76 to 1168.46)	-0.82 (-0.86 to -0.78)
Nepal	285.57	8007.29 (7977.59 to 8037.08)	298.91	3801.39 (3787.68 to 3815.13)	-2.82 (-3.31 to -2.32)
Netherlands	77.46	1935.38 (1921.72 to 1949.11)	81.44	1969.41 (1955.82 to 1983.09)	0.24 (0.16 to 0.31)
New Zealand	8.29	994.23 (972.86 to 1015.96)	12.78	976.82 (959.93 to 993.96)	0.14 (-0.11 to 0.38)
Nicaragua	13.23	2262.05 (2222.63 to 2302.03)	23.05	1426.55 (1408.12 to 1445.17)	-1.35 (-1.47 to -1.23)
Niger	39.64	3237.15 (3204.74 to 3269.82)	103.23	2976.76 (2958.27 to 2995.35)	-0.26 (-0.28 to -0.23)
Nigeria	411.55	3079.17 (3069.54 to 3088.83)	982.55	2359.13 (2354.4 to 2363.86)	-0.77 (-0.83 to -0.71)
Niue	0	808.58 (207.75 to 2159.86)	0	648.57 (118.19 to 2134.44)	-0.69 (-0.7 to -0.67)
North Macedonia	2.95	633.55 (610.86 to 656.88)	3.27	501.43 (484.37 to 519.06)	-0.64 (-0.83 to -0.45)
Northern Mariana Islands	0.07	733.47 (564.61 to 940.56)	0.09	611.64 (486.24 to 764.09)	-0.56 (-0.57 to -0.54)
Norway	27.52	2543.17 (2513.13 to 2573.48)	31.89	2330.48 (2304.86 to 2356.34)	-0.54 (-0.75 to -0.32)

Oman	7.39	3191.2 (3117.73 to 3266.03)	21.38	2256.73 (2225.88 to 2287.93)	-1.1 (-1.12 to -1.07)
Pakistan	1261.48	6878.58 (6866.51 to 6890.67)	2237.31	4322.75 (4317.03 to 4328.47)	-1.52 (-1.61 to -1.42)
Palau	0.02	700.84 (441.56 to 1062.4)	0.03	587.02 (390.03 to 871.81)	-0.57 (-0.59 to -0.54)
Palestine	9.48	3446.43 (3376.96 to 3516.99)	26.29	2696.13 (2663.48 to 2729.1)	-0.88 (-1.04 to -0.72)
Panama	8.93	1921.74 (1881.6 to 1962.55)	14.86	1430.89 (1407.97 to 1454.11)	-0.89 (-0.97 to -0.82)
Papua New Guinea	5.77	848.35 (826.27 to 870.89)	16.81	772.89 (761.18 to 784.75)	-0.31 (-0.33 to -0.29)
Paraguay	48.34	6319.67 (6262.59 to 6377.17)	78.56	4480.37 (4448.92 to 4512)	-1 (-1.05 to -0.95)
Peru	145.78	3601.92 (3583.21 to 3620.71)	243.59	2603.63 (2593.29 to 2614)	-1.01 (-1.05 to -0.97)
Philippines	88.52	797.29 (791.97 to 802.63)	172.41	676.06 (672.87 to 679.27)	-0.49 (-0.52 to -0.47)
Poland	55.46	680.1 (674.38 to 685.85)	86.81	758.37 (753.33 to 763.45)	0.86 (0.31 to 1.41)
Portugal	35.55	1417.89 (1403.18 to 1432.72)	36.86	1217.27 (1204.53 to 1230.13)	-0.56 (-0.66 to -0.46)
Puerto Rico	28.92	3144.8 (3108.61 to 3181.31)	16.92	2017.13 (1986.61 to 2048.02)	-1.54 (-1.63 to -1.44)
Qatar	1.66	2734.65 (2596.66 to 2878.76)	11.4	2015.64 (1977.61 to 2054.35)	-0.98 (-1.04 to -0.93)
Republic of Korea	32.81	326.55 (322.99 to 330.13)	42.02	272.54 (269.9 to 275.2)	-0.57 (-0.7 to -0.43)
Republic of Moldova	8.16	805.77 (788.3 to 823.53)	6.53	591.84 (577.54 to 606.52)	-0.3 (-0.64 to 0.03)
Romania	26.48	500.11 (494.1 to 506.18)	28.56	487.25 (481.59 to 492.98)	0.26 (0.07 to 0.45)
Russian Federation	232.65	706.19 (703.28 to 709.1)	294.71	681.7 (679.23 to 684.18)	0.88 (0.45 to 1.31)
Rwanda	24.05	2199.79 (2171.16 to 2228.73)	46.3	1675.94 (1660.48 to 1691.52)	-0.88 (-0.9 to -0.85)
Saint Kitts and Nevis	0.23	3205.58 (2779.06 to 3685.61)	0.37	2222.86 (2002.77 to 2462.25)	-1.17 (-1.19 to -1.15)
Saint Lucia	0.84	3392.53 (3161.88 to 3636.44)	1.01	2000.07 (1878.07 to 2128.63)	-1.7 (-1.75 to -1.65)
Saint Vincent and the Grenadines	0.63	3441.9 (3169.88 to 3732.46)	0.7	2405.14 (2229.3 to 2591.76)	-1.15 (-1.18 to -1.13)
Samoa	0.17	683.01 (583.03 to 795.61)	0.24	613.93 (538.95 to 696.54)	-0.19 (-0.26 to -0.12)
San Marino	0.11	1866.02 (1535.25 to 2247.89)	0.18	1879.79 (1603.57 to 2197.54)	0.12 (0.08 to 0.15)
Sao Tome and Principe	0.51	2962.5 (2705.93 to 3237.67)	0.91	2007.84 (1878.86 to 2143.63)	-1.21 (-1.3 to -1.13)
Saudi Arabia	63.37	3015.26 (2991.44 to 3039.24)	186.71	1784.06 (1775.92 to 1792.24)	-1.84 (-1.95 to -1.72)
Senegal	35.22	2972.8 (2941.15 to 3004.73)	68.35	2302.18 (2284.71 to 2319.75)	-0.7 (-0.75 to -0.66)
Serbia	12.96	538.49 (529.26 to 547.85)	10.82	427.25 (419.23 to 435.41)	-0.62 (-0.85 to -0.39)
Seychelles	0.09	718.48 (574.38 to 888.53)	0.17	634.81 (543.86 to 737.66)	-0.28 (-0.35 to -0.22)
Sierra Leone	20.11	2880.81 (2839.64 to 2922.47)	36.56	2274.43 (2250.54 to 2298.53)	-0.69 (-0.77 to -0.61)
Singapore	2.67	324.83 (312.55 to 337.49)	4.99	270.67 (263.08 to 278.51)	-0.58 (-0.7 to -0.46)
Slovakia	8.86	714.89 (700.06 to 729.96)	10.09	587.71 (576.27 to 599.38)	-0.17 (-0.35 to 0.02)
Slovenia	3.24	655.94 (633.55 to 678.95)	3.81	643.73 (623.4 to 664.75)	0.37 (0.18 to 0.57)
Solomon Islands	0.46	957.8 (871.07 to 1051.05)	1.12	775.87 (730.88 to 822.96)	-0.68 (-0.7 to -0.65)
Somalia	30.14	2282.85 (2256.74 to 2309.2)	70.46	1990.76 (1975.84 to 2005.77)	-0.4 (-0.43 to -0.36)
South Africa	116.58	1649.01 (1639.41 to 1658.67)	195.46	1328.05 (1322.14 to 1333.97)	-0.66 (-0.68 to -0.64)
South Sudan	17.88	2109.32 (2077.57 to 2141.48)	34.83	1883.33 (1863.48 to 1903.34)	-0.31 (-0.34 to -0.28)
Spain	157.03	1703.56 (1695.14 to 1712.02)	233.13	1731.07 (1723.84 to 1738.34)	0.29 (0.2 to 0.38)
Sri Lanka	25.17	673.39 (665.02 to 681.85)	35.09	588.73 (582.58 to 594.94)	-0.36 (-0.43 to -0.28)
Sudan	107.01	3353.26 (3333.03 to 3373.58)	227.94	2674.88 (2663.78 to 2686.02)	-0.72 (-0.86 to -0.59)
Suriname	2.55	3265.63 (3139.24 to 3395.88)	3.6	2445.37 (2366.13 to 2526.64)	-0.87 (-0.93 to -0.81)
Sweden	51.12	2266.47 (2246.7 to 2286.39)	27.93	1115.42 (1102.33 to 1128.64)	-2.59 (-2.89 to -2.29)
Switzerland	38.23	2000.39 (1980.35 to 2020.59)	46.51	1981.61 (1963.56 to 1999.81)	-0.18 (-0.68 to 0.33)
Syrian Arab Republic	55.25	3240 (3212.78 to 3267.4)	92.61	2297.98 (2283.05 to 2313.01)	-1.07 (-1.13 to -1.01)
Taiwan (Province of China)	26.98	619.54 (612.02 to 627.13)	34	462.14 (457.18 to 467.15)	-1.03 (-1.24 to -0.82)
Tajikistan	3.62	623.53 (603.01 to 644.58)	9	457.94 (448.49 to 467.54)	-0.6 (-0.81 to -0.39)
Thailand	82.78	659.08 (654.55 to 663.64)	105.97	506.01 (502.92 to 509.12)	-0.85 (-0.88 to -0.82)

Timor-Leste	1.27	940.81 (889.06 to 994.9)	1.82	737.08 (703.29 to 772.08)	-0.82 (-0.87 to -0.77)
Togo	17.52	2947.22 (2902.71 to 2992.28)	39.4	2185.14 (2163.42 to 2207.03)	-0.86 (-0.94 to -0.78)
Tokelau	0	948.41 (175.21 to 3017.01)	0	795.06 (136.54 to 2615.15)	-0.55 (-0.57 to -0.53)
Tonga	0.13	777.28 (648.47 to 924.43)	0.15	682.43 (577.43 to 801.19)	-0.39 (-0.41 to -0.37)
Trinidad and Tobago	7.61	2919.54 (2853.52 to 2986.77)	7.76	2064.23 (2018.38 to 2110.95)	-0.87 (-0.98 to -0.76)
Tunisia	38.62	2710.11 (2682.92 to 2737.52)	74.23	2153.89 (2138.4 to 2169.49)	-0.62 (-0.65 to -0.59)
Turkey	264.3	2501.22 (2491.63 to 2510.84)	472.33	1997.27 (1991.57 to 2002.98)	-0.86 (-0.94 to -0.78)
Turkmenistan	2.85	593.7 (571.81 to 616.22)	5.51	474.14 (461.69 to 486.83)	-0.22 (-0.5 to 0.06)
Tuvalu	0.02	774.63 (449.09 to 1249.61)	0.02	620.82 (349.6 to 1021.53)	-0.7 (-0.72 to -0.68)
Uganda	56.7	2334.02 (2314.32 to 2353.85)	135.02	1925.44 (1914.93 to 1935.99)	-0.03 (-0.46 to 0.39)
Ukraine	94.83	735.58 (730.9 to 740.28)	80.27	604.2 (600.01 to 608.43)	0.18 (-0.19 to 0.55)
United Arab Emirates	5.91	2698.29 (2624.75 to 2773.57)	42.4	1742.17 (1724.66 to 1759.88)	-1.33 (-1.35 to -1.3)
United Kingdom	308.35	2102.35 (2094.91 to 2109.81)	433.03	2531.47 (2523.92 to 2539.04)	0.53 (0.27 to 0.79)
United Republic of Tanzania	88.18	2225.78 (2210.76 to 2240.88)	206.41	1850.11 (1842.04 to 1858.21)	-0.47 (-0.54 to -0.41)
United States of America	636.5	982.66 (980.24 to 985.09)	538.95	653.93 (652.19 to 655.68)	-2.85 (-3.78 to -1.91)
United States Virgin Islands	1.03	3396.38 (3191.11 to 3612.01)	0.51	2522.27 (2304.88 to 2757.05)	-0.97 (-1.02 to -0.91)
Uruguay	11.54	1591.72 (1562.81 to 1621.04)	12.66	1402.45 (1378.08 to 1427.16)	-0.39 (-0.45 to -0.33)
Uzbekistan	15.77	589.84 (580.53 to 599.26)	37.93	453.27 (448.72 to 457.86)	-0.48 (-0.71 to -0.26)
Vanuatu	0.21	876.2 (759.51 to 1006.42)	0.45	706.91 (643.13 to 775.43)	-0.7 (-0.72 to -0.68)
Venezuela (Bolivarian Republic of)	71.06	2021.29 (2006.08 to 2036.6)	102.9	1375.82 (1367.41 to 1384.28)	-1.09 (-1.16 to -1.02)
Vietnam	81.13	737.84 (732.6 to 743.1)	155.3	567.15 (564.33 to 569.98)	-0.79 (-0.83 to -0.75)
Yemen	64.14	3560.02 (3532.04 to 3588.19)	177.12	2821.5 (2808.15 to 2834.9)	-0.69 (-0.85 to -0.54)
Zambia	26.35	2254.42 (2226.7 to 2282.41)	62.42	1809 (1794.47 to 1823.63)	-0.64 (-0.69 to -0.58)
Zimbabwe	29.46	1933.46 (1910.85 to 1956.28)	50.62	1620.91 (1606.62 to 1635.29)	-0.35 (-0.42 to -0.29)

S17 Table. The global prevalence of premenstrual syndrome among women aged 15-49 years in 1990 and 2021, along with the trends and changes observed between these years, by country and territories

Countries	Number of cases (Thousands) in 1990	Age-standardized rate per 100,000 population (95% UI) in 2021	Number of cases (Thousands) in 2021	Age-standardized rate per 100,000 population (95% UI) in 2021	Estimated annual percentage changes (95% CI) from 1990 to 2021
Afghanistan	795.01	38528.36 (38436.94 to 38619.99)	2607.97	39327.29 (39276.68 to 39377.96)	0 (-0.02 to 0.02)
Albania	368.49	45295.03 (45143.55 to 45446.95)	283.39	46123.76 (45953.47 to 46294.55)	0.06 (0.04 to 0.07)
Algeria	2199.36	40142.87 (40086.52 to 40199.28)	4646.85	40748.99 (40711.78 to 40786.22)	-0.04 (-0.09 to 0.01)
American Samoa	4.7	40432.11 (39236.44 to 41659.96)	4.87	41823.38 (40644.76 to 43029.1)	0.13 (0.11 to 0.14)
Andorra	6.57	43847.42 (42783.22 to 44932.98)	8.75	42597.13 (41661.32 to 43551.18)	-0.07 (-0.08 to -0.05)
Angola	873.78	39737.52 (39650.62 to 39824.58)	3045.16	41114.23 (41066.54 to 41161.97)	0.1 (0.07 to 0.13)
Antigua and Barbuda	7.45	46366.27 (45300.62 to 47453.62)	11.34	46577.41 (45719.88 to 47447.86)	0.04 (0.03 to 0.05)
Argentina	3438.69	43150.45 (43104.74 to 43196.19)	5097.21	42657.14 (42620.08 to 42694.22)	-0.02 (-0.04 to -0.01)
Armenia	390.55	45572.98 (45425.64 to 45720.74)	345.53	45805.23 (45648.88 to 45962.03)	-0.03 (-0.05 to -0.01)
Australia	1941.2	43309.75 (43248.73 to 43370.83)	2562.94	41995.4 (41943.49 to 42047.35)	-0.07 (-0.09 to -0.06)
Austria	869.99	43833.29 (43740.72 to 43926.01)	852.13	42483.17 (42391.45 to 42575.07)	-0.08 (-0.08 to -0.07)
Azerbaijan	822.15	45242.86 (45139.18 to 45346.75)	1275.96	45854.98 (45774.46 to 45935.61)	-0.02 (-0.04 to 0.01)
Bahamas	32.56	45679.98 (45172.87 to 46191.98)	49.86	46259.28 (45853.61 to 46667.71)	0.01 (0 to 0.03)
Bahrain	45.31	40583.64 (40184.69 to 40986.41)	137.57	41783.74 (41562.84 to 42005.56)	0.07 (0.04 to 0.1)
Bangladesh	11115.47	47519.33 (47489.65 to 47549.03)	22218.74	48709.08 (48688.74 to 48729.43)	0.05 (0.03 to 0.07)
Barbados	31.46	46087.4 (45574.52 to 46605.11)	33.12	46246.48 (45745.7 to 46751.63)	0.03 (0.01 to 0.04)
Belarus	1189.87	46718.89 (46634.56 to 46803.34)	1005.03	46205 (46110.98 to 46299.2)	-0.07 (-0.1 to -0.05)
Belgium	1066.73	43500.75 (43417.91 to 43583.72)	1061.58	42279.33 (42198.09 to 42360.7)	-0.08 (-0.09 to -0.07)
Belize	17.35	43212.76 (42525.17 to 43910.64)	54.26	45475.39 (45089.79 to 45863.64)	0.15 (0.13 to 0.16)
Benin	388.16	37442.07 (37318.17 to 37566.34)	1121.49	36363.13 (36292.96 to 36433.43)	-0.12 (-0.15 to -0.09)
Bermuda	7.89	45571.17 (44559.97 to 46601.74)	6.28	45878.96 (44714.69 to 47068.62)	0.03 (0.01 to 0.04)
Bhutan	62.04	45708.22 (45324.64 to 46094.72)	97.07	47039.42 (46742.02 to 47338.39)	0.1 (0.09 to 0.12)
Bolivia (Plurinational State of)	636.22	42619.08 (42511.48 to 42726.92)	1401.07	45132.12 (45057.07 to 45207.27)	0.18 (0.16 to 0.19)
Bosnia and Herzegovina	549.51	47304.82 (47179.35 to 47430.55)	346.27	46472.34 (46314.56 to 46630.56)	-0.06 (-0.07 to -0.05)
Botswana	129.24	42055.47 (41812.49 to 42299.72)	293.87	43162.84 (43006.16 to 43319.98)	0.08 (0.07 to 0.1)
Brazil	18691.07	48542.2 (48519.76 to 48564.66)	28735.96	48418.25 (48400.47 to 48436.03)	0 (-0.02 to 0.03)
Brunei Darussalam	25.76	39033.18 (38535.95 to 39536.45)	48.71	38731.57 (38386.95 to 39078.7)	-0.05 (-0.06 to -0.04)
Bulgaria	968.81	46194.47 (46101.95 to 46287.13)	666.02	45772.06 (45657.52 to 45886.83)	-0.07 (-0.08 to -0.05)
Burkina Faso	740.32	37176.58 (37088.83 to 37264.5)	1902.16	36423.47 (36369.29 to 36477.73)	-0.04 (-0.07 to -0.02)
Burundi	497.03	40982.82 (40863.06 to 41102.9)	1306.3	43143.25 (43065.2 to 43221.42)	0.15 (0.13 to 0.18)
Cabo Verde	29.66	39800.65 (39311.25 to 40295.51)	59.47	39963.8 (39640.44 to 40289.35)	0.03 (-0.01 to 0.06)
Cambodia	1076.04	44225.76 (44139.16 to 44312.52)	2064.65	45943.83 (45880.76 to 46006.96)	0.1 (0.06 to 0.13)
Cameroon	829.48	37005.91 (36922.38 to 37089.61)	2805.56	37455.2 (37409.73 to 37500.72)	0.03 (-0.01 to 0.06)
Canada	3185.86	42913.85 (42866.4 to 42961.34)	3591.52	42254.43 (42210.25 to 42298.65)	-0.11 (-0.14 to -0.09)
Central African Republic	244.88	39987.73 (39822.79 to 40153.26)	563.05	42128.24 (42014.87 to 42241.87)	0.17 (0.15 to 0.2)
Chad	469.31	37417.84 (37306.42 to 37529.56)	1309	36179.47 (36113.76 to 36245.29)	-0.08 (-0.12 to -0.04)
Chile	1539.96	43363 (43293.7 to 43432.38)	2011.88	42440.17 (42381.3 to 42499.11)	-0.05 (-0.06 to -0.04)
China	141444.6	44521.95 (44514.42 to 44529.48)	140527.3	43716.46 (43709.02 to 43723.91)	-0.19 (-0.27 to -0.11)
Colombia	3864.62	45523.61 (45476.76 to 45570.5)	6052.94	46097.31 (46060.53 to 46134.12)	0.05 (0.04 to 0.07)
Comoros	40.33	39996.38 (39586.89 to 40409.53)	85.07	44256.75 (43957.13 to 44558)	0.34 (0.33 to 0.35)
Congo	222.07	41396.91 (41214.48 to 41580.04)	605.23	42872.09 (42763.17 to 42981.24)	0.09 (0.06 to 0.13)
Cook Islands	1.87	42002.17 (40078.16 to 44001.42)	1.83	42567.59 (40628.78 to 44578.83)	0.06 (0.05 to 0.07)

Costa Rica	342.45	44875.45 (44720.64 to 45030.75)	600.82	46001.61 (45885.06 to 46118.4)	0.08 (0.06 to 0.09)
Côte d'Ivoire	973.96	37852.68 (37772.41 to 37933.1)	2429.19	37713.33 (37664.53 to 37762.18)	-0.01 (-0.05 to 0.02)
Croatia	567.42	46780.82 (46658.84 to 46903.04)	420.57	46041.59 (45899.77 to 46183.78)	-0.05 (-0.06 to -0.04)
Cuba	1414.47	46842.77 (46764.38 to 46921.25)	1150.48	46205.1 (46119.55 to 46290.78)	-0.06 (-0.07 to -0.05)
Cyprus	85.26	43144.2 (42854.79 to 43435.11)	155.77	42584.71 (42364.41 to 42806.07)	-0.04 (-0.06 to -0.02)
Czechia	1209.56	46293.75 (46210.4 to 46377.22)	1070.77	45721.13 (45630.24 to 45812.18)	-0.07 (-0.09 to -0.06)
Democratic People's Republic of Korea	2237.86	40317.56 (40264.03 to 40371.14)	2798.63	42302.32 (42252.58 to 42352.1)	0.18 (0.17 to 0.18)
Democratic Republic of the Congo	3268.18	39998.96 (39953.55 to 40044.42)	8580.42	41654.81 (41625.89 to 41683.74)	0.11 (0.07 to 0.15)
Denmark	574.98	43539.1 (43425.98 to 43652.45)	535.67	42067.04 (41953.49 to 42180.85)	-0.09 (-0.1 to -0.07)
Djibouti	39.1	41489 (41054.84 to 41927.29)	138.27	43000.34 (42773.02 to 43228.66)	0.11 (0.09 to 0.13)
Dominica	7.33	44400.16 (43356.35 to 45464.66)	7.55	46127.21 (45091.59 to 47181.1)	0.15 (0.13 to 0.17)
Dominican Republic	816.81	44956.45 (44854.21 to 45058.9)	1298.09	45062.5 (44984.76 to 45140.34)	0.03 (0.02 to 0.04)
Ecuador	1080.21	44015.86 (43929.9 to 44101.97)	2129.35	45269.54 (45208.57 to 45330.57)	0.1 (0.09 to 0.11)
Egypt	4937.86	39377.96 (39342.52 to 39413.43)	10391.82	40746.96 (40722.03 to 40771.92)	0.05 (0.03 to 0.08)
El Salvador	565.06	44722.96 (44601.54 to 44844.67)	814.33	46003.38 (45903 to 46103.93)	0.12 (0.1 to 0.13)
Equatorial Guinea	37.93	40112.43 (39695.64 to 40532.89)	147.86	41787.06 (41567.74 to 42007.4)	0.14 (0.1 to 0.18)
Eritrea	318.15	41804.4 (41653.18 to 41956.08)	705.9	43633.04 (43529.28 to 43737.02)	0.14 (0.13 to 0.15)
Estonia	177.9	46211.46 (45996.23 to 46427.48)	128.94	45625.61 (45370.04 to 45882.41)	-0.1 (-0.12 to -0.08)
Eswatini	74.76	40451.9 (40144.99 to 40760.91)	130.36	42270.1 (42035.11 to 42506.26)	0.15 (0.14 to 0.17)
Ethiopia	4763.83	43695.14 (43653.91 to 43736.4)	12468.01	46049.8 (46023.07 to 46076.54)	0.19 (0.18 to 0.2)
Fiji	82.14	42975.83 (42676.9 to 43276.52)	95.75	41968.32 (41702.57 to 42235.39)	-0.06 (-0.07 to -0.05)
Finland	558.59	43339.79 (43224.54 to 43455.3)	488.54	42475.5 (42355.49 to 42595.78)	-0.05 (-0.06 to -0.04)
France	6370.63	43773.68 (43739.56 to 43807.82)	6050.5	42162.4 (42128.52 to 42196.31)	-0.09 (-0.11 to -0.08)
Gabon	85.91	40983.13 (40690.22 to 41277.96)	201.92	42334.84 (42146.58 to 42523.81)	0.09 (0.06 to 0.12)
Gambia	80.46	37898.63 (37616.44 to 38182.84)	224.41	38095.47 (37930.31 to 38261.29)	0.04 (0 to 0.07)
Georgia	628.09	45845.18 (45731.22 to 45959.37)	362	45109.08 (44959.52 to 45259.07)	-0.1 (-0.12 to -0.09)
Germany	8619.79	43954.48 (43924.83 to 43984.15)	7326.91	42333.52 (42302.38 to 42364.68)	-0.1 (-0.11 to -0.1)
Ghana	1306.08	38757.44 (38688 to 38826.98)	3468.65	38934.44 (38892.77 to 38976.14)	0.04 (0.01 to 0.07)
Greece	1110.56	43978.83 (43897.02 to 44060.76)	944.9	42557.41 (42468.39 to 42646.59)	-0.09 (-0.11 to -0.07)
Greenland	5.99	41695.54 (40613.21 to 42801.27)	5.23	41262.87 (40146.62 to 42403.53)	-0.03 (-0.04 to -0.02)
Grenada	8.57	45420.42 (44423.18 to 46437.43)	11.79	46340.9 (45503.5 to 47190.49)	0.1 (0.08 to 0.11)
Guam	14.24	41357.01 (40665.51 to 42059.14)	14.81	41184.59 (40522.07 to 41855.55)	-0.02 (-0.04 to -0.01)
Guatemala	743.78	41952.16 (41852.81 to 42051.71)	1971.04	45492.42 (45427.86 to 45557.06)	0.28 (0.25 to 0.31)
Guinea	496.71	37804.48 (37696.99 to 37912.24)	1208.24	38081.35 (38010.73 to 38152.08)	0.05 (0.03 to 0.07)
Guinea-Bissau	83.84	37868.66 (37601.01 to 38137.98)	193.78	38251.01 (38074.72 to 38428.05)	0.06 (0.02 to 0.1)
Guyana	88.46	44884 (44575.37 to 45194.55)	92	45654.47 (45357.1 to 45953.4)	0.07 (0.06 to 0.08)
Haiti	649.18	43198.61 (43090.13 to 43307.33)	1604.78	45538.97 (45468.03 to 45610.01)	0.16 (0.14 to 0.17)
Honduras	435.57	42913.67 (42780.12 to 43047.59)	1285.43	45883.38 (45802.79 to 45964.09)	0.23 (0.21 to 0.25)
Hungary	1178.65	45867.09 (45783.56 to 45950.74)	999.66	45890.82 (45797.39 to 45984.41)	-0.03 (-0.04 to -0.01)
Iceland	27.36	42408.59 (41905.22 to 42916.86)	33.42	41631.04 (41184.05 to 42081.9)	-0.05 (-0.06 to -0.04)
India	98764.69	49831.72 (49821.71 to 49841.73)	190490.8	50521.98 (50514.78 to 50529.18)	0.04 (0.03 to 0.04)
Indonesia	23432.58	49916.82 (49896.03 to 49937.62)	37051.14	49035.76 (49019.95 to 49051.56)	-0.09 (-0.11 to -0.08)
Iran (Islamic Republic of)	5328.95	44122.42 (44083.01 to 44161.86)	11012.24	45628.05 (45600.5 to 45655.61)	0.03 (0 to 0.06)
Iraq	1429	37777.67 (37712.42 to 37843.03)	4205.23	41052.83 (41013.21 to 41092.49)	0.23 (0.22 to 0.25)
Ireland	375.61	42850.24 (42712.68 to 42988.15)	498.52	42002.74 (41883.75 to 42122.01)	-0.06 (-0.07 to -0.04)
Israel	513.43	42338.36 (42221.41 to 42455.58)	907.98	40672.91 (40589.16 to 40756.79)	-0.1 (-0.11 to -0.09)

Italy	6743.9	47086.5 (47050.9 to 47122.13)	5618.29	45908.04 (45868.75 to 45947.35)	-0.07 (-0.08 to -0.06)
Jamaica	263.96	45766.3 (45583.53 to 45949.72)	361.45	46775.39 (46622.51 to 46928.67)	0.09 (0.08 to 0.1)
Japan	14456.46	44606.72 (44583.41 to 44630.04)	11114.61	44319.21 (44292.26 to 44346.17)	-0.06 (-0.09 to -0.02)
Jordan	301.41	39062.25 (38912.07 to 39212.94)	1247.83	41343.56 (41270.34 to 41416.89)	0.14 (0.12 to 0.15)
Kazakhstan	1838.12	45150.37 (45083.98 to 45216.85)	2123.27	44433.96 (44373.77 to 44494.23)	-0.11 (-0.14 to -0.08)
Kenya	2163.51	43928.97 (43865.56 to 43992.46)	6089.69	47043.09 (47004.45 to 47081.77)	0.2 (0.19 to 0.22)
Kiribati	7.53	41469.67 (40507.82 to 42451.4)	13.41	42338.94 (41619.26 to 43068.96)	0.07 (0.05 to 0.09)
Kuwait	167.51	41331.22 (41123.63 to 41539.83)	653.23	42070.02 (41962.6 to 42177.7)	0.07 (0.05 to 0.1)
Kyrgyzstan	447.54	44052.77 (43916.87 to 44189.07)	771.54	45056.61 (44955.63 to 45157.77)	0.01 (-0.02 to 0.04)
Lao People's Democratic Republic	409.71	43860.9 (43721.81 to 44000.37)	894.5	45594.58 (45499.26 to 45690.07)	0.15 (0.14 to 0.17)
Latvia	301.46	46302.87 (46137.21 to 46469)	181.66	45914.6 (45696.64 to 46133.47)	-0.08 (-0.1 to -0.06)
Lebanon	296.01	40793.54 (40644.62 to 40942.9)	639.86	42081.4 (41977 to 42186.02)	0.1 (0.09 to 0.11)
Lesotho	154.53	42018.31 (41805.07 to 42232.44)	213.35	43237.59 (43049.14 to 43426.77)	0.1 (0.08 to 0.11)
Liberia	195.16	37234.68 (37059.46 to 37410.65)	522.19	38610.53 (38503.66 to 38717.64)	0.1 (0.07 to 0.13)
Libya	326.05	38902.33 (38758.66 to 39046.47)	855.09	42488.16 (42397.94 to 42578.53)	0.22 (0.19 to 0.26)
Lithuania	428.41	46509.14 (46369.78 to 46648.82)	267.14	46186.56 (46008.26 to 46365.46)	-0.08 (-0.1 to -0.05)
Luxembourg	42.57	43338.47 (42923.45 to 43756.93)	66.87	42208.04 (41881.7 to 42536.62)	-0.05 (-0.06 to -0.04)
Madagascar	1066.09	41604.61 (41521.13 to 41688.25)	3119.66	44588.42 (44537.24 to 44639.66)	0.22 (0.2 to 0.24)
Malawi	853.05	39723.22 (39633.8 to 39812.83)	2076.79	43245.17 (43183 to 43307.42)	0.28 (0.25 to 0.31)
Malaysia	1966.57	44700.74 (44636.65 to 44764.92)	3756.87	44611.45 (44566.18 to 44656.77)	0 (-0.01 to 0)
Maldives	19.23	42741.04 (42089.77 to 43401.05)	52.83	45198.91 (44808.68 to 45591.94)	0.05 (-0.02 to 0.13)
Mali	675.43	36970.76 (36879.64 to 37062.07)	1865.6	36479.65 (36424.18 to 36535.2)	-0.03 (-0.06 to 0)
Malta	41.31	42956.32 (42539.09 to 43376.85)	40.49	42083.56 (41664.31 to 42506.51)	-0.04 (-0.05 to -0.04)
Marshall Islands	3.92	41960.71 (40568.28 to 43399.94)	6.3	42838.64 (41782.33 to 43916.12)	0.07 (0.06 to 0.07)
Mauritania	168.25	37530.6 (37343.45 to 37718.58)	394.8	38315.49 (38191.94 to 38439.38)	0.06 (0.02 to 0.09)
Mauritius	136.97	46292.11 (46043.07 to 46542.33)	146.14	45811.75 (45575.83 to 46048.63)	-0.04 (-0.05 to -0.04)
Mexico	10006.14	46857.68 (46827.41 to 46887.97)	16934.32	48320.97 (48297.95 to 48344)	0.11 (0.1 to 0.11)
Micronesia (Federated States of)	9.22	41172.8 (40290.34 to 42073.08)	10.99	42750.64 (41945.22 to 43568.22)	0.12 (0.11 to 0.13)
Monaco	3.09	43146.5 (41585.08 to 44761.58)	3.06	41992.03 (40476.97 to 43553.12)	-0.05 (-0.06 to -0.05)
Mongolia	212.33	43569.93 (43371.59 to 43769.08)	375.35	44080.82 (43939.19 to 44222.81)	-0.05 (-0.1 to 0)
Montenegro	71.7	46006.16 (45669.31 to 46345)	66.61	45596.19 (45246.73 to 45947.83)	-0.03 (-0.04 to -0.02)
Morocco	2417.44	40269.87 (40217.42 to 40322.38)	4059.96	41871.37 (41830.65 to 41912.13)	0.11 (0.1 to 0.11)
Mozambique	1260.32	41456.03 (41381.52 to 41530.66)	3098.39	42735.6 (42685.47 to 42785.79)	0.09 (0.06 to 0.11)
Myanmar	4752.53	46779.02 (46735.33 to 46822.75)	6949.37	46150.45 (46116.08 to 46184.85)	-0.09 (-0.11 to -0.07)
Namibia	134.8	41548 (41313.4 to 41783.79)	278.32	42875.22 (42713.89 to 43037.06)	0.07 (0.05 to 0.08)
Nauru	0.96	40404.66 (37826.32 to 43134.6)	1.16	41344.7 (38963.21 to 43846.33)	0.08 (0.07 to 0.08)
Nepal	2006.32	45585.3 (45520.35 to 45650.33)	4263.04	47709.97 (47664.16 to 47755.82)	0.17 (0.12 to 0.21)
Netherlands	1749.54	43843.17 (43778 to 43908.42)	1570.27	42428.12 (42361.36 to 42494.97)	-0.09 (-0.1 to -0.07)
New Zealand	416.77	46109.15 (45969.01 to 46249.63)	540.59	45098.03 (44977.39 to 45218.92)	-0.05 (-0.06 to -0.03)
Nicaragua	377.7	43793.54 (43645.39 to 43942.16)	826.1	45754.11 (45655.07 to 45853.32)	0.13 (0.11 to 0.15)
Niger	594.58	36458.63 (36361.42 to 36556.07)	1759.49	35579.07 (35522.54 to 35635.68)	-0.07 (-0.1 to -0.05)
Nigeria	7665.47	39932.02 (39901.76 to 39962.31)	22427.91	40636.25 (40618.68 to 40653.82)	0.06 (-0.02 to 0.14)
Niue	0.21	42236.48 (36635.72 to 48476.61)	0.16	42152.87 (35883.08 to 49244.23)	-0.01 (-0.02 to 0)
North Macedonia	234.95	46229.86 (46042.9 to 46417.41)	251.05	46441.39 (46256.32 to 46627.1)	0.02 (0.01 to 0.02)
Northern Mariana Islands	5.78	42478.81 (41342.32 to 43645.09)	4.81	42417.51 (41196.16 to 43667.7)	0.02 (0.01 to 0.03)
Norway	495.52	46616.13 (46486.16 to 46746.38)	554.69	45581.12 (45460.36 to 45702.13)	-0.06 (-0.07 to -0.04)

Oman	126.03	38954.43 (38731.06 to 39178.94)	428.46	41266.98 (41141.31 to 41392.98)	0.15 (0.13 to 0.17)
Pakistan	10958.78	47717.91 (47688.67 to 47747.16)	30864.33	51547.67 (51529.23 to 51566.12)	0.24 (0.2 to 0.28)
Palau	1.73	42901.5 (40876.73 to 45010.79)	1.61	42509.83 (40390.21 to 44720.19)	-0.04 (-0.05 to -0.02)
Palestine	152	37586.24 (37384.85 to 37788.55)	512.77	41212.18 (41096.63 to 41328)	0.33 (0.31 to 0.35)
Panama	276.61	46256.51 (46079.69 to 46433.91)	485.71	45554.87 (45426.62 to 45683.4)	-0.04 (-0.06 to -0.03)
Papua New Guinea	388.09	41606.37 (41470.56 to 41742.57)	1087.71	42117.67 (42037.77 to 42197.69)	0.05 (0.04 to 0.07)
Paraguay	397.8	43379.16 (43240.37 to 43518.34)	846.71	44832.59 (44736.59 to 44928.76)	0.07 (0.06 to 0.09)
Peru	2329.09	43988.13 (43929.69 to 44046.63)	4366.48	45261.02 (45218.51 to 45303.56)	0.08 (0.07 to 0.09)
Philippines	7274.38	47944.58 (47908.48 to 47980.7)	14052.11	48325.45 (48300 to 48350.91)	0 (-0.01 to 0.01)
Poland	4727.63	49265.01 (49220.1 to 49309.94)	4449.22	49283.28 (49235.7 to 49330.9)	-0.02 (-0.03 to 0)
Portugal	1113.02	44073.41 (43991.49 to 44155.45)	1015.79	42648.18 (42562.28 to 42734.23)	-0.06 (-0.08 to -0.04)
Puerto Rico	429.3	44971.91 (44837.27 to 45106.87)	346.46	45937.5 (45783.53 to 46091.88)	0.07 (0.06 to 0.08)
Qatar	31.62	39958.66 (39501.19 to 40421.27)	244.3	41767.69 (41591.98 to 41944.08)	0.12 (0.12 to 0.13)
Republic of Korea	4955.88	39835.76 (39800.12 to 39871.42)	4563.57	38487.91 (38451.35 to 38524.49)	-0.27 (-0.61 to 0.08)
Republic of Moldova	527.98	46736.27 (46609.35 to 46863.46)	425.23	46964.49 (46817.52 to 47111.88)	-0.03 (-0.06 to 0)
Romania	2631.74	46768.54 (46711.75 to 46825.39)	1890.78	45711.16 (45643.99 to 45778.42)	-0.1 (-0.12 to -0.09)
Russian Federation	18605.34	49417.2 (49394.53 to 49439.88)	16908.72	48900.79 (48876.5 to 48925.09)	-0.09 (-0.11 to -0.06)
Rwanda	632.03	40375.84 (40270.04 to 40481.9)	1513.43	43845.03 (43773.52 to 43916.64)	0.33 (0.31 to 0.35)
Saint Kitts and Nevis	4.39	45248.81 (43834.76 to 46707.08)	7.28	46291.04 (45230.11 to 47371.69)	0.08 (0.08 to 0.09)
Saint Lucia	14.98	45394.11 (44634.76 to 46164.89)	21.57	46855.8 (46228.51 to 47489.95)	0.12 (0.11 to 0.13)
Saint Vincent and the Grenadines	11.63	45906.71 (45024.36 to 46804.77)	12.9	46261.42 (45464.87 to 47068.86)	0.01 (0 to 0.02)
Samoa	15.05	42523.64 (41809.3 to 43248.46)	19.67	41310.15 (40725.03 to 41902.06)	-0.13 (-0.14 to -0.11)
San Marino	2.71	43630.3 (41998.12 to 45311.1)	3.07	42388.07 (40853.8 to 43968.35)	-0.06 (-0.07 to -0.05)
Sao Tome and Principe	9.22	38313.38 (37485.12 to 39157.47)	21.73	39936.12 (39397.2 to 40481.17)	0.17 (0.12 to 0.21)
Saudi Arabia	1164	37818.87 (37746.51 to 37891.35)	4379.35	41826.54 (41786.84 to 41866.27)	0.32 (0.28 to 0.35)
Senegal	600.66	36917.67 (36819.22 to 37016.36)	1456.67	38726.57 (38661.79 to 38791.44)	0.13 (0.11 to 0.16)
Serbia	1082.93	46300.23 (46213.02 to 46387.56)	950.84	46132.86 (46038.95 to 46226.93)	-0.02 (-0.04 to -0.01)
Seychelles	7.96	45631.69 (44583.71 to 46700.86)	10.92	44708.34 (43869.34 to 45559.78)	-0.09 (-0.11 to -0.06)
Sierra Leone	364.91	38239.8 (38109.17 to 38370.84)	832.32	38401.51 (38315.13 to 38488.07)	0.04 (0 to 0.07)
Singapore	380.28	40736.86 (40606.3 to 40867.76)	591.36	39393.76 (39285.22 to 39502.61)	-0.13 (-0.15 to -0.11)
Slovakia	617.43	46184.42 (46069 to 46300.07)	594.99	46026.72 (45905.48 to 46148.24)	-0.04 (-0.06 to -0.03)
Slovenia	233.97	46732.71 (46543.2 to 46922.82)	196.72	45670.52 (45461.14 to 45880.72)	-0.1 (-0.11 to -0.09)
Solomon Islands	28.1	39573.11 (39081.05 to 40070.58)	69.91	41504.71 (41194.11 to 41817.2)	0.17 (0.16 to 0.19)
Somalia	653.52	39775.65 (39676.69 to 39874.82)	1920.52	41161.74 (41100.94 to 41222.61)	0.1 (0.09 to 0.12)
South Africa	4317.27	45888.14 (45843.38 to 45932.94)	7170.17	46055.83 (46022.03 to 46089.66)	0 (-0.01 to 0.01)
South Sudan	490.76	39939.23 (39819.66 to 40059.13)	908.14	40366.58 (40280.99 to 40452.32)	0.04 (0.03 to 0.06)
Spain	4251.26	44313.52 (44271.31 to 44355.76)	4376.68	43140.83 (43098.5 to 43183.19)	-0.06 (-0.08 to -0.04)
Sri Lanka	2129.43	46946.89 (46883.06 to 47010.79)	2595.09	45735.2 (45679.43 to 45791.01)	-0.12 (-0.13 to -0.1)
Sudan	1700.21	38774.51 (38713.79 to 38835.32)	4495.8	41315.64 (41276.6 to 41354.72)	0.21 (0.19 to 0.22)
Suriname	42.93	45381.43 (44941.75 to 45824.6)	66.24	45617.86 (45271 to 45966.74)	0.01 (0.01 to 0.02)
Sweden	895.69	43202.14 (43111.94 to 43292.5)	980.09	44471 (44382.11 to 44560.03)	-0.02 (-0.06 to 0.02)
Switzerland	776.91	43633.64 (43535.82 to 43731.65)	846.62	42512.3 (42419.71 to 42605.08)	-0.06 (-0.07 to -0.04)
Syrian Arab Republic	1004.91	39424.17 (39342.06 to 39506.43)	1599.52	42197.8 (42128.54 to 42267.16)	0.17 (0.15 to 0.19)
Taiwan (Province of China)	2181.51	40003.51 (39949.54 to 40057.55)	2302.05	39826.29 (39772.83 to 39879.83)	0 (-0.01 to 0.01)
Tajikistan	498.43	43480.69 (43348.59 to 43613.18)	1138.75	45368.74 (45284.45 to 45453.15)	0.13 (0.12 to 0.14)
Thailand	7454.44	47369.57 (47334.96 to 47404.2)	7613.27	45957.03 (45923.65 to 45990.43)	-0.14 (-0.16 to -0.11)

Timor-Leste	77.03	42606.04 (42296.27 to 42917.79)	149.63	44644.22 (44407.37 to 44882.12)	0.16 (0.14 to 0.17)
Togo	311.13	38301.02 (38159.67 to 38442.85)	825.07	39073.13 (38987.79 to 39158.63)	0.05 (0.02 to 0.08)
Tokelau	0.15	42283.96 (35584.88 to 49991.66)	0.13	42012.77 (35195.72 to 49811.99)	-0.02 (-0.03 to -0.01)
Tonga	8.33	38494.49 (37637.28 to 39367.6)	10.11	40585.38 (39789.7 to 41393.6)	0.19 (0.18 to 0.2)
Trinidad and Tobago	140.29	46169.48 (45924.72 to 46415.35)	158.82	46001.45 (45772.88 to 46230.94)	-0.04 (-0.06 to -0.02)
Tunisia	810.99	41589.85 (41496.01 to 41683.87)	1340.49	42560.75 (42488.08 to 42633.52)	0.04 (0.02 to 0.06)
Turkey	5824.21	42833.36 (42797.69 to 42869.05)	9446.68	43027.63 (43000.1 to 43055.18)	-0.05 (-0.08 to -0.02)
Turkmenistan	367.75	43355.49 (43205.03 to 43506.42)	552.96	44314.78 (44197.84 to 44431.97)	0.05 (0.03 to 0.08)
Tuvalu	0.96	39734.31 (37248.7 to 42350.38)	1.19	41623.46 (39263.83 to 44094.42)	0.16 (0.14 to 0.17)
Uganda	1426.21	39647.53 (39577.04 to 39718.13)	4232.36	42634.89 (42591.79 to 42678.04)	0.25 (0.22 to 0.28)
Ukraine	6338.77	49702.39 (49663.59 to 49741.21)	5183.39	50276.91 (50231.6 to 50322.26)	-0.02 (-0.04 to 0.01)
United Arab Emirates	131.15	39166.8 (38939.44 to 39395.53)	769.99	40849.41 (40745.61 to 40953.46)	0.16 (0.11 to 0.2)
United Kingdom	6556.07	45868.77 (45833.51 to 45904.06)	6912.05	44681.13 (44647.55 to 44714.72)	-0.07 (-0.09 to -0.06)
United Republic of Tanzania	2295.93	40346.72 (40290.96 to 40402.55)	6107.78	42556.04 (42521.14 to 42590.96)	0.14 (0.13 to 0.16)
United States of America	24922.45	36959.97 (36945.39 to 36974.56)	32930.17	43037.74 (43023.01 to 43052.48)	0.61 (0.52 to 0.71)
United States Virgin Islands	12.77	44197.88 (43431.1 to 44975.22)	7.83	45185.39 (44176.28 to 46213.13)	0.1 (0.08 to 0.11)
Uruguay	325.31	43521.42 (43371.86 to 43671.36)	355.75	42381.65 (42242.1 to 42521.56)	-0.06 (-0.07 to -0.05)
Uzbekistan	2025.12	43395.09 (43330.23 to 43460.04)	3989.42	44598.14 (44554.25 to 44642.08)	0.08 (0.06 to 0.11)
Vanuatu	13.4	39768.82 (39068.98 to 40479.78)	31.81	41419.48 (40959.12 to 41884.07)	0.15 (0.14 to 0.16)
Venezuela (Bolivarian Republic of)	2110.89	44582.94 (44520.91 to 44645.04)	3125.62	45130.49 (45080.1 to 45180.93)	0.03 (0.01 to 0.04)
Vietnam	7798.37	47341.63 (47306.35 to 47376.94)	12127.67	46944.32 (46917.69 to 46970.96)	-0.07 (-0.1 to -0.05)
Yemen	965.52	37456.65 (37378.3 to 37535.15)	3318.28	41160.93 (41115.57 to 41206.33)	0.3 (0.29 to 0.31)
Zambia	684.69	39670.71 (39568.98 to 39772.67)	2018.38	42618.41 (42556.74 to 42680.15)	0.22 (0.18 to 0.25)
Zimbabwe	935.01	41008.25 (40919.19 to 41097.49)	1670.16	42291.38 (42225.71 to 42357.15)	0.07 (0.06 to 0.08)

S18 Table. The global prevalence of other gynecological diseases among women aged 15-49 years in 1990 and 2021, along with the trends and changes observed between these years, by country and territories

Countries	Number of cases (Thousands) in 1990	Age-standardized rate per 100,000 population (95% UI) in 2021	Number of cases (Thousands) in 2021	Age-standardized rate per 100,000 population (95% UI) in 2021	Estimated annual percentage changes (95% CI) from 1990 to 2021
Afghanistan	860.5	49728.33 (49618.91 to 49837.96)	2908.17	49882.79 (49823.33 to 49942.3)	0 (-0.01 to 0.01)
Albania	95.94	12797.35 (12713.56 to 12881.6)	77.91	12372.32 (12285.33 to 12459.8)	-0.11 (-0.13 to -0.1)
Algeria	2296.71	48728.23 (48662.66 to 48793.87)	5597.13	47751.17 (47711.5 to 47790.86)	-0.07 (-0.07 to -0.06)
American Samoa	1.38	13173.62 (12465.89 to 13915.31)	1.46	13078.36 (12411.45 to 13773.08)	-0.02 (-0.02 to -0.01)
Andorra	5.08	32684.36 (31784.06 to 33605.55)	7.05	32371.17 (31586.21 to 33173.65)	-0.03 (-0.03 to -0.03)
Angola	639.28	33135.19 (33051.87 to 33218.68)	2152.21	33074.72 (33029.77 to 33119.71)	-0.01 (-0.01 to 0)
Antigua and Barbuda	2.6	17352.93 (16679.1 to 18049.5)	4.33	17031.21 (16526.19 to 17548.81)	-0.06 (-0.07 to -0.06)
Argentina	1785.59	23109.85 (23075.93 to 23143.8)	2722.53	22567.92 (22541.11 to 22594.76)	-0.1 (-0.12 to -0.08)
Armenia	176.68	21585.33 (21480.58 to 21690.53)	169.34	20738.01 (20638.38 to 20838.07)	-0.16 (-0.18 to -0.14)
Australia	936.31	20730.05 (20688.01 to 20772.16)	1288.32	20229.29 (20194.23 to 20264.41)	-0.08 (-0.09 to -0.08)
Austria	906.58	44856.12 (44763.48 to 44948.92)	851.92	40397.39 (40310.8 to 40484.15)	-0.41 (-0.43 to -0.39)
Azerbaijan	352.83	21404.57 (21329.31 to 21480.05)	603.24	20631.24 (20578.93 to 20683.66)	-0.16 (-0.18 to -0.13)
Bahamas	11.11	17087.16 (16763.71 to 17415.76)	18.38	16881.72 (16638.15 to 17128.03)	-0.05 (-0.06 to -0.04)
Bahrain	49.91	47450.51 (47001.01 to 47904.01)	156.79	47040.59 (46807.72 to 47274.36)	-0.03 (-0.04 to -0.03)
Bangladesh	4558.43	22038.09 (22016.96 to 22059.24)	9562.56	21582.92 (21569.19 to 21596.65)	-0.08 (-0.09 to -0.07)
Barbados	11.19	17062.79 (16744.22 to 17386.34)	12.79	16868.64 (16575.25 to 17166.25)	-0.04 (-0.04 to -0.03)
Belarus	752.98	29268.99 (29202.54 to 29335.57)	684.85	28489.94 (28420.88 to 28559.15)	-0.12 (-0.13 to -0.1)
Belgium	870.74	34729.09 (34656 to 34802.29)	898.32	34482.97 (34411.23 to 34554.84)	-0.05 (-0.06 to -0.03)
Belize	5.76	17289.16 (16823.72 to 17765.73)	18.68	16875.51 (16633.08 to 17120.71)	-0.08 (-0.09 to -0.07)
Benin	305.06	33045.2 (32923.38 to 33167.4)	886.28	32869.3 (32799.21 to 32939.52)	-0.02 (-0.02 to -0.01)
Bermuda	3.07	16946.31 (16348.99 to 17562.46)	2.57	16730.1 (16073.79 to 17410.13)	-0.05 (-0.06 to -0.04)
Bhutan	25.77	21795.15 (21518.09 to 22075.28)	42.67	21047.08 (20846.32 to 21249.43)	-0.13 (-0.14 to -0.12)
Bolivia (Plurinational State of)	330.45	24434.48 (24349.72 to 24519.49)	720.74	23978.26 (23922.68 to 24033.94)	-0.06 (-0.06 to -0.06)
Bosnia and Herzegovina	146.78	12906.57 (12840.29 to 12973.13)	98.6	12376.02 (12297.61 to 12454.85)	-0.14 (-0.16 to -0.13)
Botswana	85.58	33079.32 (32849.81 to 33310.2)	220.95	32837.67 (32699.94 to 32975.87)	-0.03 (-0.03 to -0.02)
Brazil	5238.74	14564.85 (14552.16 to 14577.54)	7798.02	12801.03 (12792.03 to 12810.04)	-0.84 (-1.05 to -0.63)
Brunei Darussalam	5.33	7978.29 (7756.13 to 8206.49)	10.23	7901.62 (7748.92 to 8056.78)	-0.04 (-0.04 to -0.03)
Bulgaria	268.75	12541.23 (12493.56 to 12589.05)	198.99	12347.43 (12291.37 to 12403.71)	-0.05 (-0.06 to -0.04)
Burkina Faso	583	33113.75 (33027.16 to 33200.53)	1509.53	33038.86 (32984.63 to 33093.16)	-0.01 (-0.02 to -0.01)
Burundi	323.36	30145.87 (30038 to 30254.08)	794.43	30300.43 (30231.28 to 30369.72)	0.01 (0.01 to 0.01)
Cabo Verde	20.74	33009.74 (32535.9 to 33489.44)	47.7	32841.91 (32544.67 to 33141.38)	-0.02 (-0.02 to -0.02)
Cambodia	194.46	8641.18 (8601.71 to 8680.81)	381.03	8574.51 (8547.09 to 8602)	-0.03 (-0.03 to -0.03)
Cameroon	643.72	32858.75 (32776.11 to 32941.57)	2189.7	32779.39 (32734.87 to 32823.97)	-0.01 (-0.01 to -0.01)
Canada	1056.73	13707.17 (13680.94 to 13733.45)	1163.06	13176.66 (13152.59 to 13200.77)	-0.11 (-0.13 to -0.09)
Central African Republic	178.22	33149.98 (32992.18 to 33308.4)	390.66	33221.36 (33115.65 to 33327.35)	0.01 (0.01 to 0.01)
Chad	367.57	33129.61 (33020.16 to 33239.37)	1017.83	33188.57 (33122.18 to 33255.07)	0 (0 to 0)
Chile	775.91	22889.32 (22837.86 to 22940.87)	1089.39	22197.91 (22156.19 to 22239.68)	-0.43 (-0.6 to -0.26)
China	47590.76	16513.05 (16508.26 to 16517.84)	40955.8	11682.52 (11678.87 to 11686.18)	-1.58 (-1.86 to -1.29)
Colombia	1171.96	15075.92 (15047.8 to 15104.09)	1910.56	14616.54 (14595.79 to 14637.31)	-0.1 (-0.1 to -0.09)
Comoros	25.74	30023.19 (29648.32 to 30401.96)	54.19	30020.82 (29767.24 to 30276.09)	0 (0 to 0)
Congo	148.31	33007.69 (32834.42 to 33181.73)	434.55	32896.37 (32798.15 to 32994.83)	-0.01 (-0.01 to -0.01)
Cook Islands	0.54	13271.98 (12158.82 to 14464.6)	0.55	13082.14 (12006.96 to 14231.2)	-0.04 (-0.05 to -0.04)

Costa Rica	104.3	14779.42 (14686.9 to 14872.44)	188.78	14265.08 (14200.69 to 14329.69)	-0.12 (-0.12 to -0.11)
Côte d'Ivoire	731.15	33015.42 (32936.2 to 33094.81)	1941.82	32947.13 (32899.7 to 32994.62)	-0.01 (-0.01 to -0.01)
Croatia	194	15641.98 (15572.35 to 15711.84)	151.47	15509.08 (15429.9 to 15588.62)	-0.4 (-0.68 to -0.12)
Cuba	479.04	17161.06 (17111.95 to 17210.28)	451.48	16863.27 (16813.42 to 16913.23)	-0.07 (-0.07 to -0.06)
Cyprus	76.11	38242.06 (37970.59 to 38515.01)	147.52	37501.72 (37306.26 to 37698.19)	-0.25 (-0.43 to -0.07)
Czechia	289.34	10898.79 (10858.67 to 10939.02)	276.68	10589.8 (10548.71 to 10631.04)	0.2 (0 to 0.4)
Democratic People's Republic of Korea	926.61	17404.2 (17368.39 to 17440.07)	1176.12	17362.56 (17331.11 to 17394.06)	-0.01 (-0.02 to -0.01)
Democratic Republic of the Congo	2334.1	33039.5 (32995.96 to 33083.09)	5926.16	33144.37 (33117.16 to 33171.61)	0.01 (0.01 to 0.01)
Denmark	447.11	33518.15 (33419.47 to 33617.06)	430.98	33083.77 (32984.31 to 33183.48)	-0.03 (-0.09 to 0.03)
Djibouti	23.98	30157.41 (29764.96 to 30554.27)	96.17	30106.68 (29915.55 to 30298.82)	-0.01 (-0.01 to -0.01)
Dominica	2.48	17185.84 (16504.41 to 17889.36)	2.71	16813.35 (16185.51 to 17459.7)	-0.07 (-0.08 to -0.06)
Dominican Republic	272.41	17373.29 (17305.81 to 17440.99)	474.05	16984.12 (16935.66 to 17032.68)	-0.09 (-0.1 to -0.08)
Ecuador	560.14	25171.88 (25104.21 to 25239.71)	1132.37	24564.93 (24519.58 to 24610.35)	-0.08 (-0.15 to -0.02)
Egypt	5564.43	48018.63 (47978.22 to 48059.05)	11516.77	46915.26 (46887.99 to 46942.54)	-0.07 (-0.07 to -0.07)
El Salvador	169.28	15034.4 (14960.75 to 15108.35)	250.9	14508.03 (14451.06 to 14565.19)	-0.11 (-0.12 to -0.1)
Equatorial Guinea	27.6	33116.99 (32720.5 to 33517.37)	103.18	32571.43 (32368.45 to 32775.5)	-0.07 (-0.07 to -0.06)
Eritrea	199.75	30186.13 (30050.95 to 30321.81)	447.52	30240.89 (30151.21 to 30330.78)	0 (0 to 0)
Estonia	114.94	29005.71 (28837.99 to 29174.21)	85.98	27793.15 (27604.59 to 27982.84)	-0.18 (-0.19 to -0.16)
Eswatini	50.97	32786.37 (32492.72 to 33082.26)	92.99	32731.75 (32516.61 to 32948.1)	0 (-0.01 to 0)
Ethiopia	2538.84	27769.14 (27733.93 to 27804.4)	6276.92	27816.57 (27794.2 to 27838.96)	0 (0 to 0)
Fiji	23.77	13369.69 (13197.77 to 13543.45)	29.6	13212.43 (13062.15 to 13364.05)	-0.04 (-0.04 to -0.03)
Finland	411.25	30775.01 (30680.2 to 30870.07)	363.35	30517.67 (30418.03 to 30617.58)	-0.04 (-0.36 to 0.27)
France	4826.01	32986.53 (32956.98 to 33016.1)	4733.48	32473.4 (32443.89 to 32502.93)	-0.05 (-0.05 to -0.04)
Gabon	58.5	32977.07 (32698.32 to 33257.88)	141.24	32721.39 (32548.9 to 32894.61)	-0.02 (-0.02 to -0.02)
Gambia	60.55	33055.81 (32776.97 to 33336.8)	168.54	32977.3 (32815.05 to 33140.25)	-0.01 (-0.01 to -0.01)
Georgia	285.51	21091.72 (21013.88 to 21169.79)	186.18	21624.36 (21525.25 to 21723.87)	0.1 (0.08 to 0.12)
Germany	7410.42	36420.52 (36394.2 to 36446.85)	6493.18	35751.86 (35724.14 to 35779.6)	-0.1 (-0.13 to -0.06)
Ghana	983.55	33075.13 (33007.75 to 33142.62)	2726.86	32812.87 (32773.38 to 32852.4)	-0.03 (-0.03 to -0.02)
Greece	828.67	32811.47 (32740.81 to 32882.24)	751.34	32424.28 (32348.49 to 32500.23)	-0.04 (-0.05 to -0.03)
Greenland	2.01	13760.93 (13148.33 to 14396.77)	1.71	13150.34 (12530.52 to 13794.33)	-0.13 (-0.14 to -0.11)
Grenada	2.87	17371.16 (16721.1 to 18042.39)	4.24	16979.12 (16469.24 to 17501.35)	-0.07 (-0.08 to -0.07)
Guam	4.38	13248.99 (12849.41 to 13659.52)	4.7	13118.21 (12744.11 to 13500.82)	-0.04 (-0.04 to -0.04)
Guatemala	240.02	15208 (15145.43 to 15270.79)	594.43	14770 (14731.97 to 14808.11)	-0.09 (-0.1 to -0.08)
Guinea	401.7	33047.02 (32943.12 to 33151.2)	934.48	33064.15 (32995.23 to 33133.19)	0 (0 to 0)
Guinea-Bissau	64.12	33084.36 (32821.8 to 33348.69)	151.23	33114 (32942.51 to 33286.28)	0 (0 to 0)
Guyana	29.81	17353.16 (17150.05 to 17558.33)	32.53	16992.06 (16806.52 to 17179.19)	-0.07 (-0.07 to -0.06)
Haiti	233.47	17491.75 (17419.16 to 17564.6)	583.82	17376.83 (17331.92 to 17421.83)	-0.02 (-0.03 to -0.02)
Honduras	136.66	15215.78 (15132.37 to 15299.58)	384.54	14735.15 (14687.95 to 14782.47)	-0.1 (-0.1 to -0.09)
Hungary	332.61	12572.72 (12529.64 to 12615.91)	293.45	12237.28 (12191.45 to 12283.26)	-0.09 (-0.1 to -0.08)
Iceland	19.85	30850.65 (30420.68 to 31285.55)	25.09	30512.98 (30135.73 to 30894.04)	-0.15 (-0.24 to -0.07)
India	37794	20888.45 (20881.7 to 20895.19)	72218.21	19755.37 (19750.8 to 19759.94)	-0.24 (-0.28 to -0.2)
Indonesia	3624.2	8670.01 (8660.91 to 8679.12)	4511.36	5922.44 (5916.98 to 5927.92)	-1.72 (-2.02 to -1.42)
Iran (Islamic Republic of)	4907.05	48205.03 (48160.94 to 48249.16)	12459.05	47999.27 (47972.28 to 48026.27)	-0.07 (-0.12 to -0.03)
Iraq	1581.56	47789.09 (47712.17 to 47866.13)	4624.26	47692.75 (47649.01 to 47736.52)	-0.02 (-0.03 to 0)
Ireland	278.42	32779.11 (32657.17 to 32901.41)	392.49	32350.74 (32247.39 to 32454.36)	-0.04 (-0.05 to -0.04)
Israel	385.4	32806.36 (32701.92 to 32911.08)	716.01	32456.52 (32381.25 to 32531.94)	-0.04 (-0.04 to -0.03)

Italy	4888.21	34157.06 (34126.72 to 34187.42)	2789.53	21471.06 (21445 to 21497.14)	-1.74 (-1.91 to -1.57)
Jamaica	85.83	17223.53 (17104.45 to 17343.32)	128.39	16886.16 (16793.64 to 16979.08)	-0.07 (-0.07 to -0.06)
Japan	3410.26	10549.29 (10537.9 to 10560.69)	2096.66	8186.42 (8174.99 to 8197.86)	-1.21 (-1.49 to -0.92)
Jordan	308.62	47910.45 (47734.27 to 48087.17)	1331.35	46907.69 (46827.65 to 46987.83)	-0.08 (-0.09 to -0.08)
Kazakhstan	828.78	21338.71 (21291.76 to 21385.75)	1029.35	20524.82 (20485.06 to 20564.65)	-0.14 (-0.16 to -0.13)
Kenya	1063.62	27644.6 (27589.78 to 27699.52)	3051.83	27563.07 (27531.51 to 27594.66)	0.27 (0.16 to 0.38)
Kiribati	2.24	13425.28 (12860.05 to 14011.35)	4.04	13355.26 (12943.45 to 13777.71)	-0.01 (-0.02 to -0.01)
Kuwait	178.71	46855.35 (46625.39 to 47086.37)	784.53	46128.82 (46024.53 to 46233.37)	-0.08 (-0.09 to -0.07)
Kyrgyzstan	196.01	21526.84 (21426.26 to 21627.85)	356.68	21128.89 (21059.17 to 21198.78)	-0.06 (-0.08 to -0.05)
Lao People's Democratic Republic	73.71	8637.81 (8574.08 to 8701.94)	162.69	8536.32 (8494.51 to 8578.3)	-0.04 (-0.04 to -0.04)
Latvia	183	27209.63 (27084.87 to 27334.84)	115.24	26410.98 (26255.4 to 26567.4)	-0.13 (-0.25 to 0)
Lebanon	331.37	48250.08 (48084.11 to 48416.51)	754.32	47507.62 (47399.71 to 47615.74)	-0.05 (-0.06 to -0.05)
Lesotho	109.78	33027.91 (32831.21 to 33225.55)	145.73	32907.19 (32734.97 to 33080.17)	-0.02 (-0.02 to -0.01)
Liberia	152.89	32949.72 (32776.65 to 33123.59)	401.69	32873.4 (32770.76 to 32976.29)	-0.02 (-0.02 to -0.01)
Libya	334.99	47804.53 (47636 to 47973.55)	969.67	47306.41 (47212.1 to 47400.87)	-0.04 (-0.05 to -0.03)
Lithuania	287.08	30760.08 (30647.53 to 30872.95)	187.68	30012.18 (29875.02 to 30149.91)	-0.35 (-0.54 to -0.16)
Luxembourg	36.51	35658.32 (35292.17 to 36027.8)	58.9	35133.12 (34846.87 to 35421.51)	-0.13 (-0.17 to -0.09)
Madagascar	666.15	30145.51 (30070.56 to 30220.62)	1854.99	30183.2 (30138.96 to 30227.49)	0 (0 to 0)
Malawi	552.15	30070.86 (29988.56 to 30153.36)	1217.37	30076.82 (30021.72 to 30132)	0 (-0.01 to 0)
Malaysia	354.93	8568.52 (8539.68 to 8597.44)	704.98	8424.73 (8404.98 to 8444.51)	-0.06 (-0.06 to -0.06)
Maldives	3.42	8641.13 (8337.76 to 8953.7)	10.47	8514.59 (8350.47 to 8681.43)	-0.05 (-0.06 to -0.05)
Mali	543.86	33122.98 (33033.15 to 33213.01)	1441.24	33075.07 (33019.29 to 33130.92)	-0.01 (-0.01 to -0.01)
Malta	35.63	36268.57 (35889.97 to 36650.43)	36.49	35776.11 (35405.11 to 36150.65)	-0.03 (-0.08 to 0.03)
Marshall Islands	1.1	13505.22 (12677.09 to 14383.31)	1.88	13369.55 (12769.83 to 13991.11)	-0.03 (-0.03 to -0.03)
Mauritania	129.44	32928.81 (32744.67 to 33113.83)	296.19	32784.58 (32664.55 to 32904.97)	-0.01 (-0.01 to -0.01)
Mauritius	24.71	8568.17 (8459.58 to 8677.96)	27.37	8441.36 (8341.08 to 8542.6)	-0.05 (-0.06 to -0.05)
Mexico	3559.5	19622.42 (19601.5 to 19643.36)	5588.26	15940.68 (15927.46 to 15953.91)	-0.2 (-0.5 to 0.09)
Micronesia (Federated States of)	2.6	13384.16 (12856.2 to 13931.07)	3.15	13276.64 (12814.64 to 13751.36)	-0.02 (-0.03 to -0.02)
Monaco	2.46	32524.04 (31225.71 to 33873.88)	2.43	32207.45 (30906.97 to 33553)	-0.03 (-0.04 to -0.03)
Mongolia	90.29	21570.69 (21422.38 to 21719.87)	186.27	21016.39 (20920.86 to 21112.27)	-0.12 (-0.13 to -0.1)
Montenegro	19.02	12456.06 (12279.17 to 12634.97)	18.69	12201.87 (12025.82 to 12380.03)	-0.08 (-0.1 to -0.07)
Morocco	2649.34	48843.56 (48783.39 to 48903.79)	4650.95	48068.04 (48024.35 to 48111.76)	-0.05 (-0.05 to -0.04)
Mozambique	816.29	30150.76 (30084.43 to 30217.21)	1851.41	30080.09 (30035.52 to 30124.71)	-0.02 (-0.02 to -0.01)
Myanmar	807.12	8655.76 (8636.31 to 8675.25)	1255.11	8539.13 (8524.18 to 8554.1)	-0.05 (-0.05 to -0.04)
Namibia	89.79	33019.08 (32796.34 to 33243.08)	199.67	32984.72 (32838.63 to 33131.33)	0 (-0.01 to 0)
Nauru	0.29	13341.62 (11828.93 to 15013.93)	0.35	13256.93 (11883.23 to 14756.53)	-0.02 (-0.02 to -0.01)
Nepal	917.49	22579.23 (22532.07 to 22626.47)	1878.34	21925.34 (21893.67 to 21957.06)	-0.04 (-0.09 to 0.01)
Netherlands	1331.82	32982.61 (32926.47 to 33038.82)	1230.57	32536.05 (32478.27 to 32593.9)	-0.04 (-0.04 to -0.03)
New Zealand	290.73	32460.75 (32342.59 to 32579.25)	321.77	26017.97 (25927.99 to 26108.21)	-0.63 (-0.78 to -0.47)
Nicaragua	114.17	15108.7 (15016.96 to 15200.94)	256.11	14631.68 (14574.8 to 14688.75)	-0.11 (-0.11 to -0.1)
Niger	471.88	33113.27 (33016.19 to 33210.59)	1342.33	33165.71 (33107.77 to 33223.73)	0 (0 to 0.01)
Nigeria	4670.8	30425.5 (30396.92 to 30454.1)	13852.49	30308.14 (30291.88 to 30324.41)	-0.01 (-0.02 to -0.01)
Niue	0.06	13336.65 (10214.64 to 17128.35)	0.05	13163.6 (9765.02 to 17406.77)	-0.04 (-0.04 to -0.04)
North Macedonia	63.8	12745.33 (12646.48 to 12844.78)	71.64	12330.98 (12239.95 to 12422.62)	-0.11 (-0.13 to -0.1)
Northern Mariana Islands	1.7	13164.26 (12514.3 to 13845.31)	1.53	13107.85 (12434.97 to 13809.6)	-0.01 (-0.01 to 0)
Norway	313.4	29307.72 (29205.02 to 29410.72)	373.34	29195.5 (29101.52 to 29289.74)	-0.81 (-1.38 to -0.24)

Oman	144.87	49129 (48867.97 to 49391.22)	501.45	47014.52 (46882.01 to 47147.34)	-0.15 (-0.16 to -0.14)
Pakistan	4056.91	20447.28 (20427.11 to 20467.46)	11061.43	19918.41 (19906.55 to 19930.29)	-0.08 (-0.08 to -0.07)
Palau	0.51	13297.35 (12149.53 to 14533.38)	0.52	13162.57 (12010.83 to 14404.57)	-0.03 (-0.04 to -0.03)
Palestine	171.43	48915.69 (48675.09 to 49157.27)	556.21	48370.13 (48240.69 to 48499.86)	-0.03 (-0.04 to -0.03)
Panama	83.08	15162.14 (15056.93 to 15267.96)	153.21	14614.48 (14541.3 to 14687.96)	-0.12 (-0.13 to -0.12)
Papua New Guinea	112.71	13535.34 (13454.35 to 13616.74)	331.62	13482.76 (13436.51 to 13529.14)	-0.01 (-0.01 to -0.01)
Paraguay	121.68	14459.73 (14376.82 to 14543.04)	262.04	14295.53 (14240.52 to 14350.71)	-0.04 (-0.05 to -0.04)
Peru	1161.71	24470.46 (24424.91 to 24516.08)	2307.18	23891.51 (23860.65 to 23922.4)	-0.08 (-0.08 to -0.08)
Philippines	1339.86	10035.54 (10018.14 to 10052.95)	3205.38	11634.68 (11621.88 to 11647.5)	0.7 (0.56 to 0.83)
Poland	975.58	9984.48 (9964.41 to 10004.58)	626.79	6456.11 (6439.69 to 6472.58)	-1.86 (-2.27 to -1.45)
Portugal	739.07	29696.64 (29628.92 to 29764.48)	646.35	25951.83 (25886.38 to 26017.43)	-0.6 (-0.7 to -0.5)
Puerto Rico	158.1	16984.98 (16901.26 to 17069.01)	131.61	16645.43 (16554.9 to 16736.36)	-0.07 (-0.08 to -0.06)
Qatar	35.45	44951.52 (44458.92 to 45449.35)	281.42	44251.69 (44081.97 to 44422.05)	-0.06 (-0.07 to -0.05)
Republic of Korea	990.9	7960.21 (7944.3 to 7976.15)	957.6	7854.65 (7838.57 to 7870.76)	-0.04 (-0.04 to -0.04)
Republic of Moldova	335.81	29455.45 (29355.05 to 29556.11)	285.13	28448.89 (28342.46 to 28555.7)	-0.14 (-0.16 to -0.12)
Romania	681.48	12354.76 (12325.3 to 12384.27)	531.07	11838.39 (11805.56 to 11871.29)	0.36 (0 to 0.72)
Russian Federation	10497.77	27444.92 (27428.13 to 27461.72)	10477.88	26695 (26678.44 to 26711.56)	-0.12 (-0.14 to -0.11)
Rwanda	404.4	30088.44 (29991.77 to 30185.39)	937.87	30121.01 (30059.16 to 30182.97)	0 (-0.01 to 0)
Saint Kitts and Nevis	1.46	17190.54 (16263.58 to 18165.25)	2.76	16890.79 (16265.36 to 17535.53)	-0.05 (-0.06 to -0.05)
Saint Lucia	4.88	17285.08 (16787.72 to 17794.73)	8.19	16959.94 (16592.92 to 17333.62)	-0.06 (-0.07 to -0.05)
Saint Vincent and the Grenadines	3.7	17354.51 (16775.61 to 17950.42)	4.77	16958.53 (16479.56 to 17448.37)	-0.08 (-0.09 to -0.08)
Samoa	3.98	13288.14 (12866.99 to 13720.53)	5.77	13221.96 (12880.17 to 13570.85)	-0.01 (-0.01 to -0.01)
San Marino	2.02	32645.77 (31232.22 to 34108.16)	2.41	32294.49 (30973.54 to 33660.83)	-0.04 (-0.04 to -0.03)
Sao Tome and Principe	6.65	32987.09 (32173.04 to 33818.09)	16.25	32852.11 (32344.62 to 33366.01)	-0.02 (-0.02 to -0.01)
Saudi Arabia	1299.68	48151.59 (48065.75 to 48237.56)	5053.09	46254.59 (46213.99 to 46295.22)	-0.12 (-0.13 to -0.12)
Senegal	460.21	33024.38 (32926.11 to 33122.9)	1107.59	32977.77 (32915.25 to 33040.4)	0 (-0.01 to 0)
Serbia	266.38	11260.75 (11218.01 to 11303.62)	238.5	10944.11 (10899.76 to 10988.61)	0.25 (0 to 0.5)
Seychelles	1.38	8548.43 (8083.73 to 9035.31)	2.11	8449.79 (8091.04 to 8820.86)	-0.04 (-0.04 to -0.03)
Sierra Leone	284.28	33116.71 (32989.51 to 33244.35)	629.23	33086.55 (33002.14 to 33171.15)	0 (-0.01 to 0)
Singapore	75.95	7963.19 (7906.23 to 8020.49)	127	7850.53 (7805.36 to 7895.97)	-0.05 (-0.06 to -0.05)
Slovakia	249.96	18690.39 (18616.99 to 18764.02)	256.74	17994.29 (17923.07 to 18065.78)	-0.52 (-0.76 to -0.27)
Slovenia	75.15	14819.89 (14714.02 to 14926.37)	68.07	14395.29 (14284.2 to 14507.17)	0.09 (-0.3 to 0.48)
Solomon Islands	8.04	13522.08 (13218.29 to 13831.67)	21.02	13403.49 (13221.88 to 13587.06)	-0.03 (-0.03 to -0.02)
Somalia	451.07	30187.65 (30098.3 to 30277.22)	1210.79	30341.41 (30286.28 to 30396.63)	0.01 (0.01 to 0.02)
South Africa	2424.22	30168.76 (30129.96 to 30207.61)	4686.59	30079.72 (30052.38 to 30107.07)	-0.02 (-0.02 to -0.01)
South Sudan	310.86	30003.73 (29893.79 to 30114.03)	587.39	29989.57 (29912.31 to 30066.99)	0 (0 to 0)
Spain	3094.27	32836.6 (32799.94 to 32873.28)	3446.18	32418.15 (32382.41 to 32453.91)	-0.04 (-0.05 to -0.03)
Sri Lanka	373.26	8604.7 (8576.81 to 8632.67)	482.75	8498.43 (8474.4 to 8522.51)	-0.04 (-0.04 to -0.04)
Sudan	1949.32	49584.53 (49513.05 to 49656.1)	4871.7	48419.5 (48375.74 to 48463.29)	-0.08 (-0.09 to -0.07)
Suriname	14.62	17285.32 (17001.88 to 17572.46)	24.71	16979.16 (16768 to 17192.35)	-0.06 (-0.07 to -0.06)
Sweden	572.84	26719.78 (26650.06 to 26789.66)	518.16	22106.94 (22046.47 to 22167.55)	-0.89 (-1.25 to -0.53)
Switzerland	652.37	35377.29 (35291.22 to 35463.54)	738.48	35003.54 (34922.78 to 35084.48)	-0.14 (-0.23 to -0.05)
Syrian Arab Republic	1061.37	48488.25 (48392.4 to 48584.26)	1689.27	47889.18 (47813.47 to 47965)	-0.04 (-0.04 to -0.03)
Taiwan (Province of China)	1199.5	22091.7 (22051.4 to 22132.07)	1805.69	30052.09 (30007.16 to 30097.09)	1.18 (0.87 to 1.5)
Tajikistan	213.7	21990.45 (21889.52 to 22091.79)	521.39	21649.25 (21589.7 to 21708.94)	-0.06 (-0.08 to -0.04)
Thailand	1291.01	8609.63 (8594.53 to 8624.74)	1474.79	8479.47 (8465.51 to 8493.46)	-0.05 (-0.05 to -0.05)

Timor-Leste	14.89	8658.04 (8515.57 to 8802.54)	25.51	8589.55 (8481.31 to 8698.9)	-0.03 (-0.03 to -0.03)
Togo	232.85	33078.69 (32940.15 to 33217.73)	651.91	33002.47 (32921.69 to 33083.43)	-0.01 (-0.01 to -0.01)
Tokelau	0.04	13439.54 (9675.92 to 18282.67)	0.04	13243.79 (9508.1 to 18002.23)	-0.05 (-0.05 to -0.05)
Tonga	2.46	13298.99 (12770.44 to 13844.54)	3.03	13158.29 (12692.5 to 13637.18)	-0.02 (-0.03 to -0.02)
Trinidad and Tobago	48.56	17069.5 (16915.78 to 17224.36)	61.45	16746.66 (16613.58 to 16880.63)	-0.07 (-0.08 to -0.07)
Tunisia	854.48	48627.98 (48522.09 to 48734.06)	1572.68	47791.08 (47716.02 to 47866.23)	-0.06 (-0.06 to -0.05)
Turkey	6468.14	51895.51 (51854.82 to 51936.23)	11146.99	49680.24 (49651 to 49709.5)	-0.13 (-0.14 to -0.13)
Turkmenistan	159.16	21399.41 (21287.07 to 21512.26)	249.02	20399.04 (20318.87 to 20479.46)	-0.17 (-0.19 to -0.16)
Tuvalu	0.32	13482.74 (12038.07 to 15060.75)	0.35	13244.88 (11892.09 to 14713.99)	-0.05 (-0.06 to -0.05)
Uganda	901.79	30111.4 (30046.22 to 30176.69)	2528.25	30087.88 (30049.48 to 30126.32)	-0.01 (-0.01 to -0.01)
Ukraine	3583.99	27353.23 (27324.88 to 27381.6)	3196.91	27007.16 (26976.87 to 27037.48)	-0.06 (-0.08 to -0.05)
United Arab Emirates	151.74	47245.41 (46985.64 to 47506.61)	960.07	46150.36 (46045.65 to 46255.31)	-0.09 (-0.1 to -0.08)
United Kingdom	5249.71	36221.99 (36190.9 to 36253.09)	4508.92	28272.71 (28246.49 to 28298.95)	-0.73 (-0.82 to -0.64)
United Republic of Tanzania	1438.94	30038.32 (29987.3 to 30089.42)	3795.51	30021.92 (29991.16 to 30052.71)	-0.01 (-0.01 to 0)
United States of America	10950.73	15732.93 (15723.58 to 15742.28)	9766.15	12571.85 (12563.95 to 12579.75)	-0.83 (-0.98 to -0.68)
United States Virgin Islands	4.96	17026.77 (16554.23 to 17509.85)	3.08	16621.14 (16032.18 to 17228.02)	-0.08 (-0.09 to -0.07)
Uruguay	170.2	23147.46 (23037.57 to 23257.75)	191.98	22504.29 (22403.46 to 22605.48)	-0.1 (-0.12 to -0.08)
Uzbekistan	873.56	21409.63 (21361.23 to 21458.13)	1882.23	20688.2 (20658.58 to 20717.85)	-0.12 (-0.15 to -0.1)
Vanuatu	4.08	13481.79 (13058.4 to 13916.87)	9.57	13376.83 (13107.73 to 13650.31)	-0.03 (-0.03 to -0.03)
Venezuela (Bolivarian Republic of)	646.89	14917.26 (14879.79 to 14954.82)	1039.97	14604.18 (14575.96 to 14632.45)	-0.08 (-0.09 to -0.06)
Vietnam	1362.05	8934.55 (8918.71 to 8950.41)	2447.02	9064.1 (9052.7 to 9075.51)	0.08 (0.06 to 0.1)
Yemen	1158.86	49843.53 (49749.46 to 49937.76)	3713.01	49967.45 (49915.67 to 50019.28)	0.01 (0 to 0.01)
Zambia	421.12	30013.74 (29919.41 to 30108.32)	1232.52	29958.82 (29904.22 to 30013.5)	0 (-0.01 to 0)
Zimbabwe	619.96	32980.84 (32895.5 to 33066.37)	1174.63	33072.19 (33011.52 to 33132.96)	0.01 (0.01 to 0.01)
